# Intramolecular thiomaleimide [2 + 2] photocycloadditions: stereoselective control for disulfide stapling and observation of excited state intermediates by transient absorption spectroscopy†

**DOI:** 10.1039/d1sc06804k

**Published:** 2022-01-28

**Authors:** Roshni Malde, Michael A. Parkes, Michael Staniforth, Jack M. Woolley, Vasilios G. Stavros, Vijay Chudasama, Helen H. Fielding, James R. Baker

**Affiliations:** Department of Chemistry, University College London 20 Gordon Street London WC1H 0AJ UK j.r.baker@ucl.ac.uk h.h.fielding@ucl.ac.uk v.chudasama@ucl.ac.uk; Department of Chemistry, University of Warwick Gibbet Hill Road Coventry CV4 7AL UK

## Abstract

Thiomaleimides undergo efficient intermolecular [2 + 2] photocycloaddition reactions and offer applications from photochemical peptide stapling to polymer crosslinking; however, the reactions are limited to the formation of the *exo* head-to-head isomers. Herein, we present an intramolecular variation which completely reverses the stereochemical outcome of this photoreaction, quantitatively generating *endo* adducts which minimise the structural disturbance of the disulfide staple and afford a 10-fold increase in quantum yield. We demonstrate the application of this reaction on a protein scaffold, using light to confer thiol stability to an antibody fragment conjugate. To understand more about this intriguing class of [2 + 2] photocycloadditions, we have used transient absorption spectroscopy (electronic and vibrational) to study the excited states involved. The initially formed S_2_ (π_1_π*) excited state is observed to decay to the S_1_ (n_1_π*) state before intersystem crossing to a triplet state. An accelerated intramolecular C–C bond formation provides evidence to explain the increased efficiency of the reaction, and the impact of the various excited states on the carbonyl vibrational modes is discussed.

## Abstract 2

Tethered bromomaleimides are described which undergo thiol conjugation and rapid regio- and stereoselective photocycloadditions to serve as disulfide staples; with the excited state intermediates observed by transient absorption spectroscopies.

## Graphical abstract

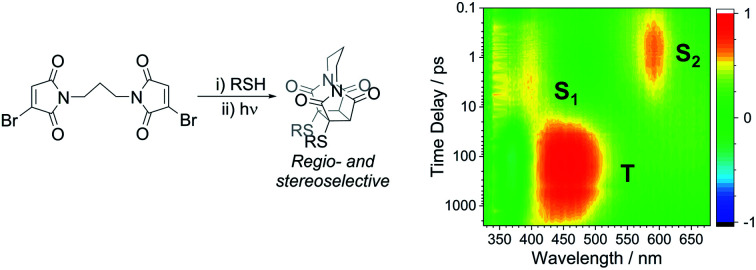

## Introduction

Maleimides are ubiquitous reagents in bioconjugations, due to their high reactivity and selectivity for reactions with thiols; in particular with cysteine residues in peptides and proteins.^1,2^ Most notably, this has been exploited for the development of eight out of the eleven approved antibody–drug conjugates (ADCs).^3,4^ Despite the prevalence of maleimides, the generated thiosuccinimide conjugates suffer from instability, due to the accessible retro-Michael pathway, with subsequent thiol exchange with cysteine-containing serum proteins such as albumin.^5–7^ A hydrolysis step serves to remove the thiol instability^6,7^ but can be competitive with the retro-Michael deconjugation.^8^ To further resolve this limitation, we previously reported on the use of monobromomaleimides as reagents for cysteine modification, forming thiomaleimide conjugates.^9^ These conjugates retain a double bond unlike the thiosuccinimide conjugates. This inhibits the retro-Michael pathway,^8^ allows controlled reversibility in thiol-rich environments^10^ and offers a second point of attachment.^11^ These reagents have been exploited in applications including the construction of antibody–drug conjugates,^12^ in fluorescence ‘turn-on’ reagents,^13^ therapeutic half-life extension^8,14^ and biotinylation.^10,15^

**Scheme 1:**
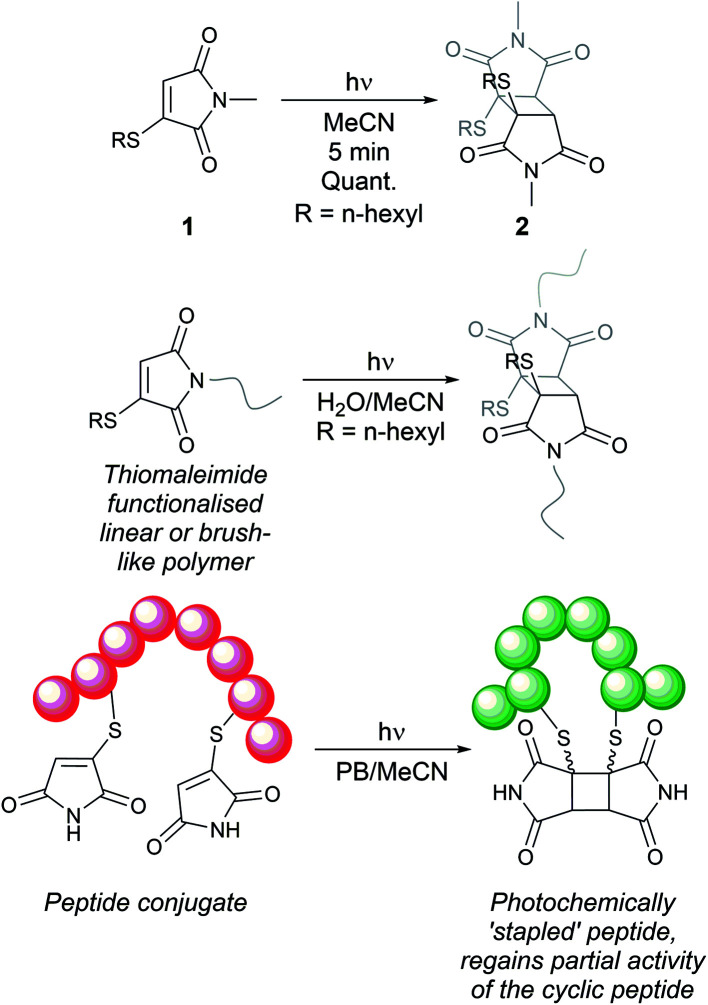
Previous work on [2 + 2] photocycloadditions of thiomaleimides.^25–27^

Notably, the conjugated double bond also introduces a chromophore in these thiomaleimide products, with potential for photochemical reactivity. Maleimides have been reported to undergo [2 + 2] photocycloadditions with alkenes and in photodimerisations,^16–19^ affording access to cyclobutanes which are challenging to synthesise *via* other routes. However, the full prospect of this maleimide photochemical reaction has not been achieved and is still underexploited with only a few reported examples in syntheses^20,21^ and applications in photo-crosslinking polymers.^22–24^ Consequently, the potential of combining the photoreactivity of maleimides with its bioconjugation capability led us to reporting on the [2 + 2] photocycloaddition of thiomaleimide 1 (Scheme 1).^25^ This occurred with complete regio- and stereo-selectivity to give the *exo* head-to-head cyclobutane product 2 in a quantitative yield. The reaction is rapid, reaching completion in just 5 min; which is notably much faster than maleimide itself under the same conditions, which requires a 1 h irradiation time. We envisaged that this highly efficient reaction could have broad utility, and have carried out initial exemplifications in photo-crosslinking polymers^26^ and in the photocyclisation and photorebridging of bioconjugates with spatiotemporal control.^27^

Encouraged by the broad potential of this ‘photoclick’ reaction of thiomaleimides, we were motivated to investigate the underlying photophysical processes. Furthermore, in the application to rebridge disulfides,^27^ we noted that the *exo* stereochemical outcome of this photocycloaddition is undesirable as the thioethers are positioned on opposite faces of the cyclobutane ring. This creates an extended distance between the sulfur atoms and an associated distortion of the peptide structure, which in turn leads to an attenuation of biological activity.^27^ Similarly, in a wider synthesis context, this photoreaction is limited to applications where only an *exo* selectivity is required. Hence our attention also turned to the challenge of inverting the stereochemical outcome of this [2 + 2] photocycloaddition to afford access to the *endo* isomer. We envisaged this would expand the scope of the photoreaction and facilitate future investigations into the improved photochemical formation of disulfide mimics.

Promisingly, De Schryver *et al.* have previously reported *endo* selective maleimide photocycloadditions through intramolecular reactions of *N*-tethered bis-maleimides.^19^ They tested a range of varying alkylene bridge lengths of 2–12 carbons and the best results were for chain lengths of 3–6, with the 3-carbon chain giving a quantitative yield in 4 h. The *endo* selective nature of this reaction can be attributed to the carbon-linker limiting the accessible conformations for the intramolecular transition state, precluding an *exo* approach. We hypothesised that a similar intramolecular reaction of a bis-thiomaleimide would selectively produce the *endo* product, with the head-to-head regioselectivity likely to be retained.

## Results and discussion

**Scheme 2:**
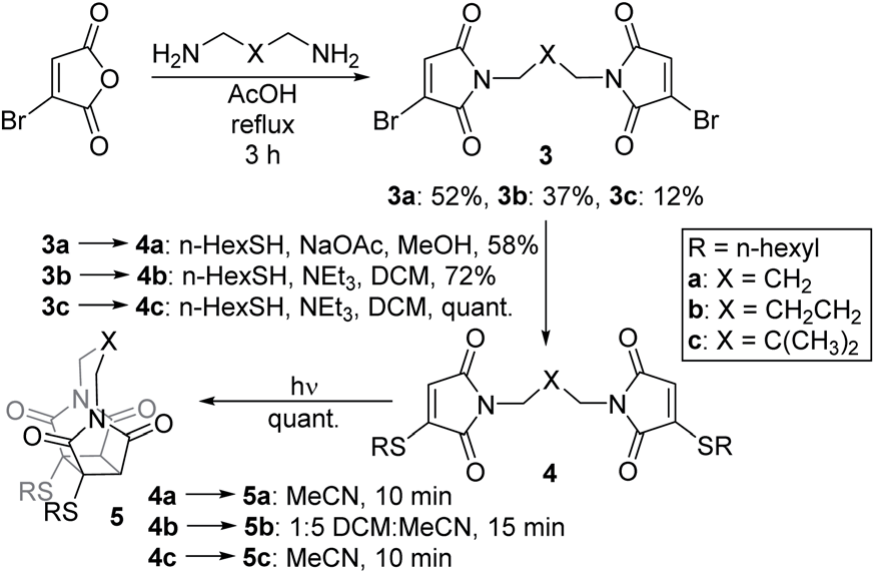
Synthesis and endo selective [2 + 2] photocycloaddition of *N*-tethered bis-thiomaleimides 4a–c.

We chose to synthesise *N*-tethered bis-thiomaleimides with 3- and 4-carbon chains (4a and 4b), and a branched chain (4c) as substrates for testing the intramolecular *endo* selective [2 + 2] photocycloaddition. These were synthesised *via* the corresponding bis-bromomaleimides 3a–c (Scheme 2).

Upon irradiation, it was found that the 3-carbon linker bis-thiomaleimide 4a gave a quantitative yield of the cyclobutane product 5a within 10 min. The 4-carbon linker bis-thiomaleimide 4b was insoluble in acetonitrile and hence was irradiated in a 1 : 5 DCM : MeCN solvent system, taking slightly longer, 15 min, to reach completion. Molecule 4c was designed to demonstrate further conformational control through the Thorpe–Ingold effect,^28^ however no further rate acceleration was observed with the reaction also reaching completion in 10 min. All three molecules displayed greatly accelerated reaction times compared to the reaction of bis-maleimides (4 h for the 3-carbon tether)^19^ and bis-dimethylmaleimides which take 20 h to reach completion.^29^

All three of the photoproducts formed were confirmed to be the *endo* isomers by considering the ^3^*J* coupling constants of the ^13^C satellite peaks in the ^1^H NMR spectra. The presence of ^13^C breaks the symmetry of the cyclobutane products causing the satellite peaks to split further into a doublet due to ^3^*J*_HH_ coupling (see ESI Fig. 2†).^30^ The presence of these doublets served as an initial confirmation of the head-to-head regioselectivity, as it proved that the protons are 3 bonds apart. The value of the ^3^*J*_HH_ coupling constant was then key to determining the stereoselectivity. In the *endo* product, the two coupling protons will be *cis* to each other, and have a dihedral angle of ∼25°. According to the Karplus equation, this would give a ^3^*J* value of ∼11 Hz.^31^ The cyclobutane satellite peak for photoproducts 5a–c is seen to have a coupling constant of 11 Hz and hence confirms that these intramolecular reactions are *endo* selective. In contrast, the *exo* product with the protons *trans* to one another will have a dihedral angle of ∼100°, giving a much smaller ^3^*J* value of 3 Hz;^31^ and this was indeed seen for the intermolecular *exo* photoproduct 2 (see ESI Fig. 1†). Thus, we have shown that the stereochemistry of the [2 + 2] photocycloaddition reaction of thiomaleimides can be inverted by utilising an intramolecular variation of the reaction.

**Scheme 3:**
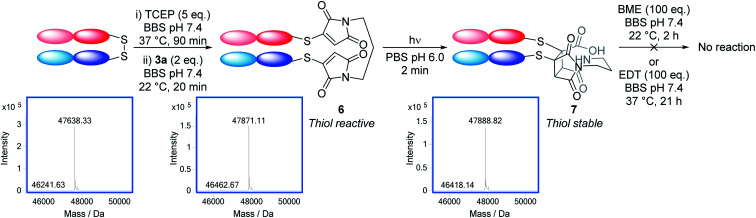
Photochemical rebridging of a trastuzumab Fab fragment to regain thiol stability along with the deconvoluted LCMS data. Native Fab observed 47 638 Da; conjugate 6 expected 47 870 Da, observed 47 871 Da; conjugate 7 expected 47 888 Da, observed 47 889 Da.

To serve as a proof-of-principle of the viability of this intramolecular *endo* selective [2 + 2] photocycloaddition on a protein scaffold, we conjugated bis-bromomaleimide 3a to a trastuzumab antibody Fab fragment (Scheme 3). This new bridging reaction was efficient, with just 2 equivalents of reagent required and full conversion achieved. Conjugate 6 was then irradiated at 365 nm for 2 min, with LCMS revealing the formation of the cycloaddition product 7; in which subsequent hydrolysis had taken place on one of the thiosuccinimides, presumably to relieve ring strain. Addition of β-mercaptoethanol or ethanedithiol to conjugate 6 confirmed that such thiomaleimides are reactive to thiols, affording a mixture of thiol addition and conjugate cleavage (see ESI Fig. 6 and 7†); whilst addition to conjugate 7 resulted in no reaction (see ESI Fig. 8 and 9†), proving this as an effective photochemical method to offer thiol stability to these thiomaleimide conjugates.

**Fig. 1:**
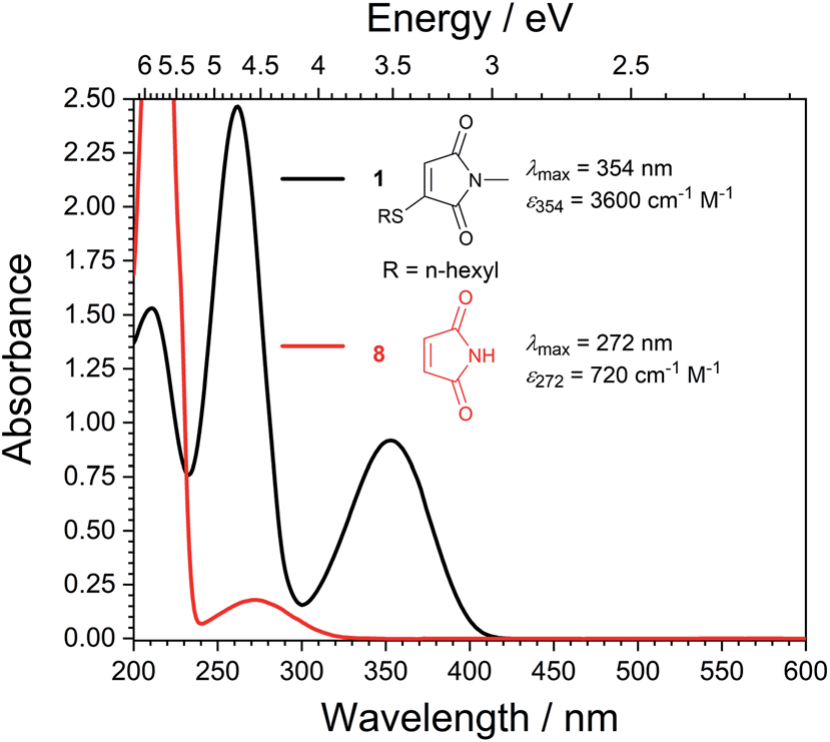
Static UV spectra for thiomaleimide 1 and maleimide 8 in MeCN (0.25 mM).

With highly efficient *endo* and *exo* stereoselective thiomaleimide photocycloaddition reactions in hand, we moved on to explore the underlying photochemical processes in more detail. Both thiomaleimide 1 and bis-thiomaleimides 4a–c undergo photocycloadditions much faster than classical maleimides, with one likely factor made apparent by analysing the differences in their absorption spectra (Fig. 1). For both thiomaleimide 1 and maleimide 8, the first band in the spectra with a significant absorption is associated with a 
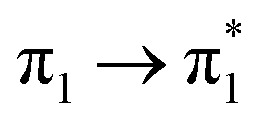
 transition to form the lowest 
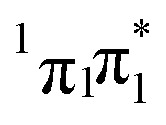
 state (from hereon, 
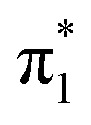
 will be referred to as π* for convenience), confirmed by *ab initio* calculations performed on maleimide 8 (ref. 32–35) and a model thiomaleimide chromophore (see ESI Table 4 and ESI Fig. 10†). For maleimide 8, this absorption peak is at 272 nm with an extinction coefficient of 720 cm^−1^ M^−1^, whilst contrastingly for thiomaleimide 1, the peak is at a longer wavelength of 354 nm with a much higher extinction coefficient of 3600 cm^−1^ M^−1^. The observed bathochromic shift suggests that thiomaleimides can be exploited for photochemical reactions using longer wavelength UV light. This would be particularly beneficial for biological systems where shorter wavelength light can be more damaging. Moreover, the increased extinction coefficient can potentially provide an explanation for the faster reaction times of thiomaleimides compared to classical maleimides. These changes in the lowest π_1_ → π* transitions from classical maleimides to thiomaleimides arise from the contribution of electron density on the sulfur atom to the maleimide π-system. This is supported by examination of the π_1_ molecular orbital (MO) involved in formation of the S_2_ (π_1_π*) state which shows electron density situated on the sulfur atom (see ESI Fig. 11†).

To further compare the efficiencies of the different [2 + 2] photocycloadditions, the quantum yields and initial rates of photocycloaddition of maleimides and thiomaleimides were measured upon irradiation using a 365 nm LED torch (Table 1). It should be noted that this wavelength was selected to be close to the dominant absorption band for the thiomaleimides (*λ*_max_ = 354 nm, see Fig. 1).

**Table 1:** The measured quantum yield *Φ* of [2 + 2] photocycloaddition for the different maleimides at a range of concentrations in MeCN (unless otherwise stated), along with their initial rates of reactant use. The power input and photon flux were calculated using the known *Φ* (0.43 ± 0.02) of the photochemical conversion of *o*-nitrobenzaldehyde into *o*-nitrosobenzoic acid (see ESI Fig. 12).^36^ The initial rates for the maleimides were calculated from the gradient of the concentration *vs.* time graph up to ∼10% conversion (see ESI Fig. 13)^34^

Molecule	Structure	Concentration/mM	*Φ*_cycloaddition_	Initial rate of reactant use/×10^−7^ M s^−1^
*N*-butylmaleimide **9**	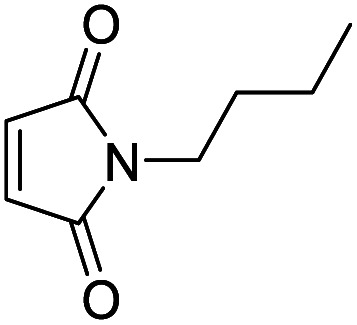	1 (DCM)	0.060 ± 0.004	2.46 ± 0.08
		1	0.019 ± 0.001	0.515 ± 0.007
Bis-maleimide **10**	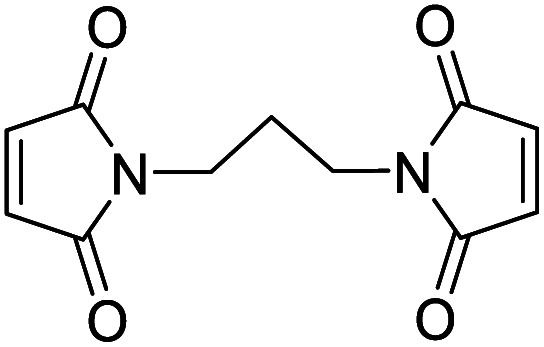	0.5	0.25 ± 0.02	3.50 ± 0.09
Thiomaleimide **1**	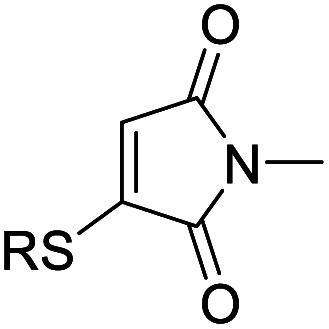	1	0.05 ± 0.02	27 ± 8
		0.5	0.022 ± 0.002	11 ± 1
		0.1	0.0056 ± 0.0004	1.36 ± 0.08
Bis-thiomaleimide **4a**	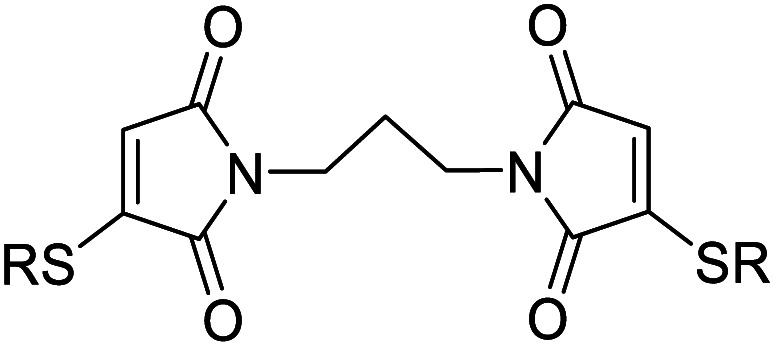	0.5	0.24 ± 0.07	67 ± 19
		0.25	0.23 ± 0.06	61 ± 15
		0.05	0.17 ± 0.04	22 ± 5
Bis-thiomaleimide **4c**	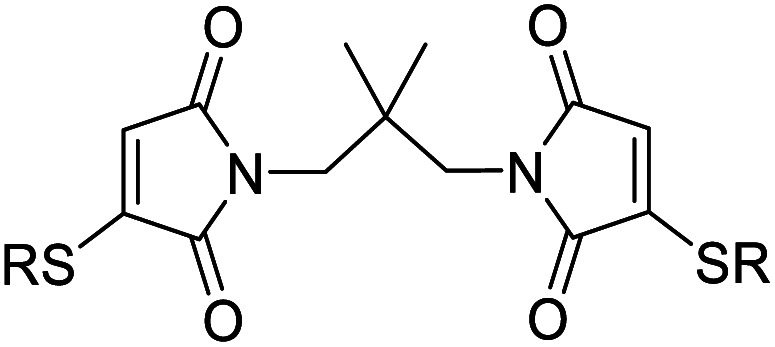	0.5	0.26 ± 0.06	73 ± 17

The cycloaddition quantum yield *Φ* of 1 mM *N*-butylmaleimide 9 in DCM was measured as a control, and at 0.060 ± 0.004 is consistent with the literature value (0.06, measured at a 300 nm irradiation wavelength^19^). The quantum yield *Φ* for 1 mM thiomaleimide 1 in MeCN was determined to be 0.05 which is on the same order of magnitude as for *N*-butylmaleimide 9 in MeCN (0.019). The initial rate proves that thiomaleimide 1 reacts at a ∼50-fold faster rate (2.7 × 10^−6^ compared to 5.2 × 10^−8^ M s^−1^), likely due to the increased absorbance at this irradiation wavelength, and is a useful improvement, especially for biological applications where longer wavelengths are preferable. Notably at lower concentrations, both the initial rate of reaction and the quantum yield *Φ* were observed to decrease proportionally, demonstrating the expected concentration dependence of a bimolecular reaction.

Bis-thiomaleimide 4a had a ∼20-fold increase in initial rate compared to the corresponding bis-maleimide 10 (6.7 × 10^−6^ compared to 3.5 × 10^−7^ M s^−1^), and the cycloaddition quantum yield *Φ* was calculated to be the same in both reactions (0.24 ± 0.07 compared to 0.25 ± 0.02). In addition, various concentrations led to the same quantum yield within experimental error indicating concentration independence, as expected for an intramolecular reaction. Compound 4c has the same rate of reaction and quantum yield as 4a within experimental error, thus it can be concluded that the *gem*-dimethyl group does not significantly increase the efficiency of the [2 + 2] photocycloaddition reaction.

Nonetheless, the key finding can be sought in the comparison of thiomaleimide 1 and bis-thiomaleimide 4a at equivalent chromophore concentrations to keep the absorbance similar, *i.e.* 0.5 mM thiomaleimide 1 and 0.25 mM bis-thiomaleimide 4a. These concentrations were chosen as these are maximum concentrations for use in a biological context. From thiomaleimide 1 to bis-thiomaleimide 4a, there was an obvious increase in the initial rate by ∼6-fold (6.1 × 10^−6^ compared to 1.1 × 10^−6^ M s^−1^) and the quantum yield *Φ* has also increased by a whole order of magnitude (0.23 compared to 0.022). This demonstrates that the intramolecular reaction offers the prospect of enhanced efficiencies in biological contexts.

**Scheme 4:**
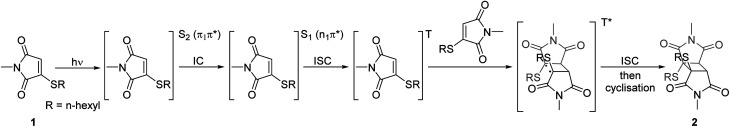
Hypothesised mechanism for the [2 + 2] photocycloaddition reaction.^35,37–39^

To further explore these thiomaleimide photocycloadditions, an investigation of the excited state species was carried out using time-resolved spectroscopic methods. Femtosecond transient electronic and vibrational absorption spectroscopy (TEAS and TVAS) enable the observation of short-lived excited state intermediates, allowing us to follow the mechanism following photoexcitation. Based on literature precedent and *ab initio* calculations, we proposed a likely mechanism upon photoexcitation at 354 nm, chosen as it is the first dominant absorption band in the reactants' UV spectra (Scheme 4, shown as a suggested mechanism for the dimerisation of thiomaleimide 1).^35,37–39^*Ab initio* calculations confirmed the 354 nm band belongs to S_2_ (π_1_π*) (see ESI Table 4†), thus we anticipated an initial π → π* electronic transition to form the S_2_ (π_1_π*) state. A recent report by Worth and co-workers indicates that the lowest ππ* excited state in maleimide undergoes rapid internal conversion (IC) to the lowest nπ* excited state,^35^ and thus a similar S_2_ (π_1_π*) → S_1_ (n_1_π*) IC was expected for thiomaleimides. The subsequent mechanism for [2 + 2] photocycloaddition is based on the widely described reaction mechanism for α,β-unsaturated carbonyls.^37–39^ This involves a fast intersystem crossing (ISC) from the S_1_ (n_1_π*) state to afford a triplet state (T).^37,38^*Ab initio* calculations (see ESI Table 4†) show that there are triplet states lower in energy than the S_1_ (n_1_π*) state, supporting this ISC pathway. The triplet state then has a sufficiently long lifetime to undergo a reaction with a ground state alkene leading to formation of the triplet 1,4-diradical (T*).^39^ This is followed by ISC back to a singlet state and a second C–C bond formation to give photoproduct 2.

**Fig. 2:**
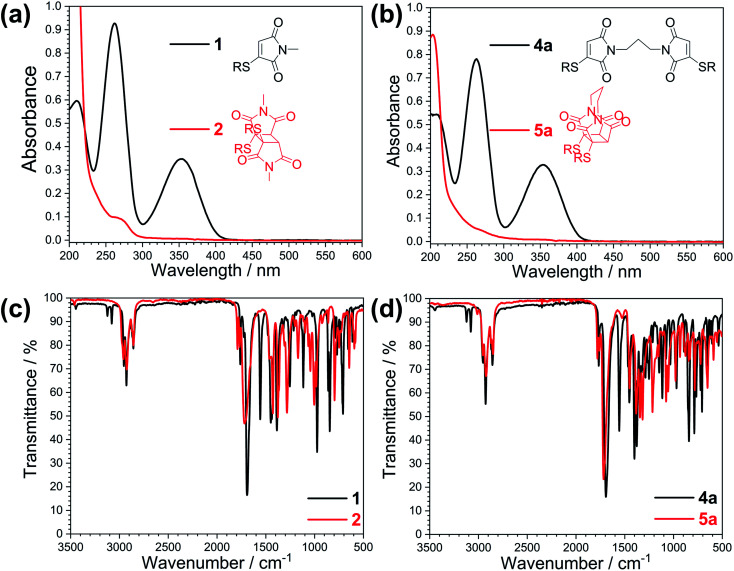
Static UV spectra for (a) thiomaleimide 1 and photoproduct 2 (0.1 mM) and (b) bis-thiomaleimide 4a and photoproduct 5a (0.05 mM). Static IR spectra for (c) thiomaleimide 1 and photoproduct 2 and (d) bis-thiomaleimide 4a and photoproduct 5a.

TEAS allows observation of ground and excited state electronic absorptions (ESAs) and hence it is important to consider the electronic absorption spectra of both the starting chromophore and the photoproduct. The UV spectra of these molecules are shown in Fig. 2. Upon irradiation of thiomaleimide 1 and bis-thiomaleimide 4a to form photoproducts 2 and 5a, respectively, the key difference is the loss of conjugation causing a full reduction of the absorbance at 354 nm to <0.01. TVAS experiments were also conducted to allow observation of ground and excited state vibrational spectra. Fig. 2 also shows the static IR spectra for thiomaleimides 1 and 4a and their respective photoproducts 2 and 5a. Thiomaleimides 1 and bis-thiomaleimide 4a show alkenyl C–H stretches at ∼3100 cm^−1^ and C=C stretches at ∼1556 cm^−1^. This is a notable difference in comparison to the spectra of the cyclobutane products 2 and 5a, in which these peaks are no longer present. Another key difference is the shift of the major C=O stretch from ∼1690 cm^−1^ in thiomaleimides 1 and 4a to ∼1720 cm^−1^ in the cyclobutane products 2 and 5a.

**Fig. 3:**
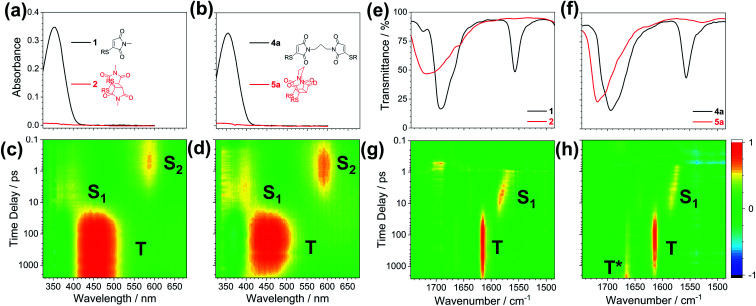
Static state UV spectra for (a) thiomaleimide 1 and its photoproduct 2 and (b) bis-thiomaleimide 4a and its photoproduct 5a. Independently normalised chirp-corrected TEAS spectra for (c) 20 mM thiomaleimide 1 (OD range: −7.2 × 10^−3^ to 7.2 × 10^−3^) and (d) 5 mM bis-thiomaleimide 4a (OD range: −2.45 × 10^−3^ to 2.45 × 10^−3^) taken following photoexcitation at 354 nm in MeCN. Note for bis-thiomaleimide 4a, the original chirp-corrected TEAS plot showed a major residual pump signal at 354 nm, causing the other signals in the region to be masked (see ESI Fig. 14a†), thus this was removed for analysis. Static state IR spectra for (e) thiomaleimide 1 and its photoproduct 2 and (f) bis-thiomaleimide 4a and its photoproduct 5a. Independently normalised TVAS spectra for (g) 20 mM thiomaleimide 1 (OD range: −8.8 × 10^−4^ to 8.8 × 10^−4^) and (h) 5 mM bis-thiomaleimide 4a (OD range: −1.81 × 10^−3^ to 1.81 × 10^−3^) taken following photoexcitation at 354 nm in MeCN. Note for bis-thiomaleimide 4a, the original TVAS plot showed a major ground state bleach (GSB) at ∼1690 cm^−1^ which obscures signals in this region (see ESI Fig. 14b†), thus this was removed for analysis.

Fig. 3 displays the TEAS data following the 354 nm photoexcitation of thiomaleimide 1 and bis-thiomaleimide 4a. The false colour plots (Fig. 3c and d) show chirp-corrected (using the KOALA package)^40^ excited state absorption (ESA) spectra arising from the photoexcited molecule and any further intermediates. Warm colours (red) represent a positive change in optical density (ΔOD) and cold colours (blue) represent a negative ΔOD. The false colour plots of the TVAS data are also shown (Fig. 3g and h). Due to strong absorption of acetonitrile in the higher wavenumber region, it was not possible to study the dynamics of the feature at 3100 cm^−1^. Instead, the experiments were conducted in the C=O stretching region (1750–1490 cm^−1^) to gain insight into thiomaleimide excited state C=O vibrations.

From the false colour plots of the TEAS experiments, three key absorption bands were observed sequentially. Specifically, the absorption band at 585 nm can be seen to decay as another band grows in at 385 nm. This in turn decays, followed by growth of the band at 455 nm. For bis-thiomaleimide 4a, this band at 455 nm is observed to decay towards the end of the experimental time window. Thus global fitting with three lifetimes was conducted using the Glotaran software^41^ to obtain timescales and decay associated spectra (DAS) for both the TEAS and TVAS experiments (see ESI Fig. 15†). The lifetimes are summarised in Table 2.

**Table 2:** Summary of lifetimes generated from the global fittings using the Glotaran software.^41^ Errors are half the FWHM of the instrument response function (TEAS) (see ESI Table 5) or standard errors obtained from the Glotaran fits (TVAS)

Lifetime	TEAS		TVAS	
	Thiomaleimide 1	Bis-thiomaleimide 4a	Thiomaleimide 1	Bis-thiomaleimide 4a
*τ*_1_ S_2_ (π_1_π*) → S_1_ (n_1_π*)	2.9 ± 0.1 ps	2.7 ± 0.1 ps	2.7 ± 0.1 ps	2.9 ± 0.2 ps
*τ*_2_ S_1_ (n_1_π*) → T	20.5 ± 0.1 ps	23.0 ± 0.1 ps	18.9 ± 0.5 ps	23.7 ± 0.9 ps
*τ*_3_ T → T*	≫2.5 ns^a^	>2.5 ns^b^	>2.5 ns^c^	2.4 ± 0.1 ns

a The lifetime extracted from the global fit is 80 ns; however, this is significantly greater than the experimental time window of 2.5 ns and thus cannot be quoted with confidence.

b The lifetime extracted from the global fit is 4 ns; this is just outside the experimental time window of 2.5 ns and hence cannot be reported with confidence.

c The lifetime extracted from the global fit is 5 ns; this is just outside the experimental time window of 2.5 ns and hence cannot be reported with confidence.

In the TEAS false colour plot for thiomaleimide 1 (Fig. 3c), we first observe a band at 585 nm which we attribute to an excited state absorption (ESA) from the initially formed S_2_ (π_1_π*) state to higher lying excited states. The disappearance of this band represents relaxation from the S_2_ (π_1_π*) excited state. The global fitting reveals a lifetime of 2.9 ± 0.1 ps for this decay, with the DAS indicating growth of the 385 nm absorption band on this same timescale. For the simple maleimide, IC from the lowest ππ* excited state to the lowest nπ* excited state occurs on a femtosecond timescale.^35^ This IC process takes place along a coordinate that involves a vibrational motion of the maleimide double bond carbon. Thus, in thiomaleimide 1, where this carbon is now bound to a heavy sulfur atom, the IC process is likely to take longer and can explain the slightly longer 2.9 ± 0.1 ps lifetime that is observed experimentally. This supports our proposition that the 385 nm absorption band represents ESA of the S_1_ (n_1_π*) state. The band at 385 nm then itself decays on the same timescale as the appearance of the 455 nm absorption band in the false colour plot (Fig. 3c). Global analysis reveals this lifetime to be 20.5 ± 0.1 ps. Previous literature reports have implied that the rate of ISC from the S_1_ (n_1_π*) to a triplet state for maleimide is >1 × 10^10^ s^−1^.^42^ This corresponds to the S_1_ (n_1_π*) state having a lifetime of <100 ps before decaying to the triplet state. Our TEAS data is consistent with this lifetime and supports our suggestion that the band at 455 nm is associated with ESA of a triplet excited state (T) from the proposed mechanism (Scheme 4). Global analysis suggests a long decay lifetime of the band at 455 nm, considerably greater than the experimental time window of 2.5 ns. Visually, we observe that this band remains until the end of the experiment, suggesting that this triplet state is a reasonably long-lived intermediate. Previous laser flash photolysis experiments by Jonsson *et al.* on *N*-methylmaleimide found that its triplet state had a self-quenching rate of 1.3 × 10^9^ M^−1^ s^−1^, consistent with an approximately diffusion-controlled rate.^43^ This is also consistent with our experimental data and so we can conclude that the triplet state will eventually evolve *via* a self-quenching pathway to form the triplet 1,4-diradical intermediate (T*), through the first C–C bond formation in the [2 + 2] photocycloaddition reaction, as shown in our hypothesised mechanism (Scheme 4).

For bis-thiomaleimide 4a, the overall dynamics are very similar to thiomaleimide 1, with slightly different lifetimes obtained. The initially populated S_2_ (π_1_π*) state has an absorption band at 585 nm, which disappears on a timescale of 2.7 ± 0.1 ps, again feeding into the S_1_ (n_1_π*) state, giving rise to the band at 385 nm. This S_1_ (n_1_π*) state decays with a lifetime of 23.0 ± 0.1 ps on the same timescale as the growth of the band at 455 nm. The 455 nm band represents ESA of a triplet state and its growth lifetime is comparable to the ISC process in the intermolecular variation. Curiously, Jonsson *et al.* concluded that the intramolecular reaction of bis-maleimide 10 proceeds *via* the singlet state and forms the corresponding singlet 1,4-diradical.^43^ However, our femtosecond TEAS experiment suggests that this is not the case, at least for thiomaleimides. The reason for this is that we clearly observe a band at 455 nm for both thiomaleimide 1 and bis-thiomaleimide 4a, which we attribute to their associated triplet states. Interestingly, for bis-thiomaleimide 4a, the 585 nm band does not decay back to zero (see ESI Fig. 16b†), with one explanation that some population remains trapped in the S_2_ (π_1_π*) state. Although this may appear unlikely, there are examples of molecules with non-Kasha rule behaviour, *e.g.* azulene.^44^ An alternative rationale could be that the long-lived absorption at 585 nm is an additional ESA feature of the triplet state, supported by the similar kinetics observed with the 455 nm band.

As before, the global fitting reveals a third lifetime >2.5 ns (outside the experimental time window) corresponding to decay of the band at 455 nm. The significance of this lifetime is that it is much shorter than seen for the intermolecular case and the decay towards the end of the experiment is clearly visible in the false colour plot (Fig. 3d). This represents the decay of the triplet excited state, whilst no decay for this band is observed for the intermolecular reaction. It is likely that this decay of the triplet state (T) is occurring as it begins to form the triplet 1,4-diradical (T*), although the ESA of T* is not observed either due to the experimental time window or the absorption of T* being weak (see below). This faster decay is consistent with the intramolecular nature of the first C–C bond forming step, where the likelihood of two reacting thiomaleimide partners reaching a favourable conformation is much higher.

**Scheme 5:**
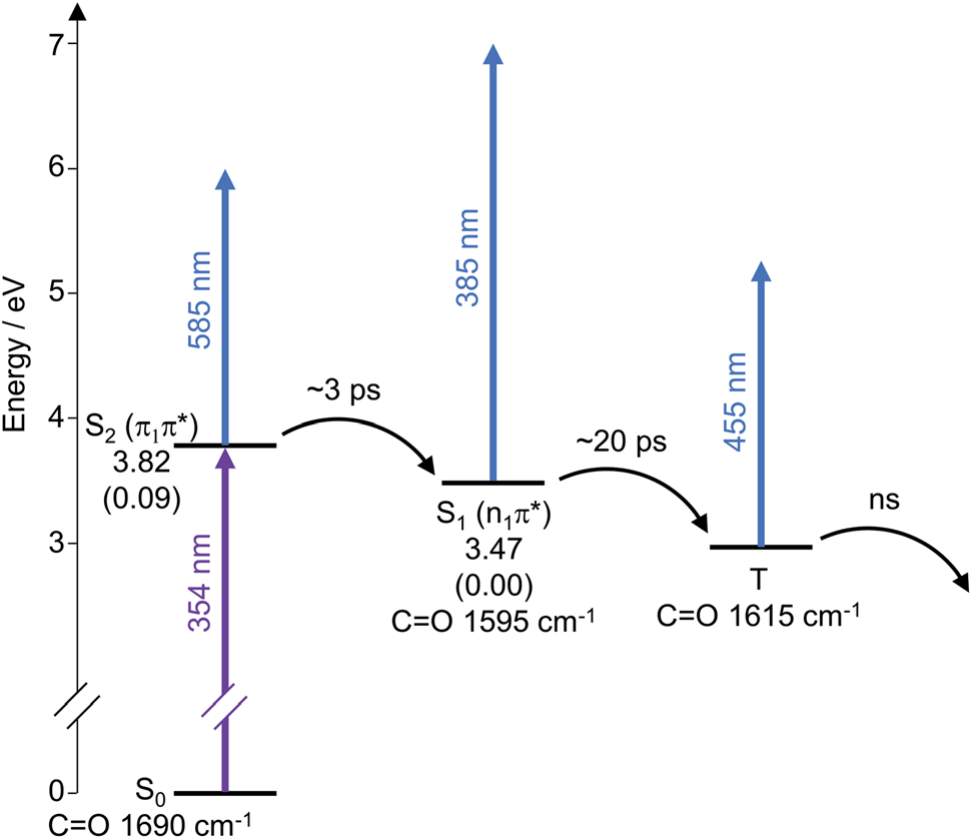
Schematic energy level diagram illustrating the electronic relaxation pathway following photoexcitation at 354 nm deduced from our TEAS and TVAS experiments. The singlet excited state energies shown are the calculated CAS-PT2 energies in eV for a model thiomaleimide (see ESI Fig. 10†) in *C*_s_ symmetry. The oscillator strengths f shown in parentheses are the calculated CAS-SCF values. These CAS calculations were performed using MolPro^45,46^ with the 6-311++G** basis set and an active space with 12 electrons and 8 orbitals. The C=O vibrational energies shown are the experimental values from the TVAS experiments.

The analysis of the TEAS data can be summarised with a dynamic scheme to show the flow of energy as the reaction proceeds after initial photoexcitation at 354 nm, showing the different processes involved (Scheme 5). The experimental data reinforces our predicted mechanism (Scheme 4) to proceed from S_2_ (π_1_π*) to S_1_ (n_1_π*) to T. The dynamic scheme also includes the experimental vibrational stretches of the C=O bond of the different excited states as inferred from the TVAS experiments (see analysis below), which show similar lifetimes to that of the TEAS experiments, supporting our characterisations.

For the TVAS experiments, it should be noted that for thiomaleimide 1, the first band observed after photoexcitation at 354 nm is at 1695 cm^−1^, however, the reason for this band is uncertain and as such, we did not consider it in our analysis. This band does not appear for bis-thiomaleimide 4a, although this region was obscured by a major ground state bleach (GSB) (see ESI Fig. 14b†). We note that for both molecules 1 and 4a, GSBs for the 1690 cm^−1^ and 1556 cm^−1^ bands are present and persist throughout the entire experimental time window, although only faintly visible in false colour plots. We chose to carry out the global fitting in the range 1560–1630 cm^−1^ as this is where the key bands appear, and no new dynamical information would be gained by including the bleaches in the global fitting.

The first new band observed for both thiomaleimide 1 and bis-thiomaleimide 4a is at 1565 cm^−1^. This does not appear straight after photoexcitation. Therefore, it is likely that the C=O stretch after initial excitement to S_2_ (π_1_π*) does not absorb in the wavenumber window explored in our experiment. The S_2_ (π_1_π*) state is formed upon promotion of a π-bonding electron into an antibonding orbital which weakens the C=O bond. This is anticipated to cause a large shift in the C=O stretch to below 1490 cm^−1^. Global fitting analysis reveals the growth of the band at 1565 cm^−1^ on timescales of 2.7 ± 0.1 ps and 2.9 ± 0.2 ps for thiomaleimide 1 and bis-thiomaleimide 4a, respectively. This indicates that the S_2_ (π_1_π*) state quickly decays to the S_1_ (n_1_π*) state, supporting the TEAS data. Therefore, the band at 1565 cm^−1^ can be attributed to the C=O stretch of the S_1_ (n_1_π*) state. This S_1_ (n_1_π*) state has a strengthened C=O stretch compared to the S_2_ (π_1_π*) state, consistent with removal of an electron from a non-bonding orbital rather than a π-bonding orbital. The false colour plots (Fig. 3g and h) show a gradual blue-shift of the band from 1565 to 1595 cm^−1^. This suggests that the S_1_ (n_1_π*) state is formed vibrationally hot and subsequently undergoes vibrational cooling to the bottom of the well. This is followed by electronic relaxation with lifetimes of 18.9 ± 0.5 ps and 23.7 ± 0.9 ps for thiomaleimide 1 and bis-thiomaleimide 4a, respectively, feeding into the state giving rise to the band at 1615 cm^−1^. These timescales are consistent with ISC from the S_1_ (n_1_π*) state to a triplet state in the TEAS experiments. Additionally, the increased wavenumber fits with a relaxation from a singlet state to a triplet state as there will be strengthening of the C=O bond as the repulsion between parallel electronic spins is lower. Together, this all supports assignment of the 1615 cm^−1^ vibrational band to the C=O stretch of a triplet state (T). Interestingly, in contrast to the S_1_ (n_1_π*), it appears that the triplet state does not undergo a vibrational cooling shift. One possibility is the formation of the triplet state towards the bottom of the well, which would occur if the triplet state was nearly degenerate with the S_1_ (n_1_π*) state. On the other hand, the lack of observable shift may be indicative of a faster vibrational cooling than ISC.

For bis-thiomaleimide 4a, a third lifetime of 2.4 ± 0.1 ns is observed. This lifetime represents decay of the 1615 cm^−1^ band and can be seen visually in the false colour plot (Fig. 3h). This is in broad agreement with the decay lifetime of the triplet state extracted from the TEAS experiment. Interestingly, a new band at 1665 cm^−1^ can be seen to grow in towards the end of the experiment. We believe that this new band likely belongs to the C=O stretch of the associated *endo* triplet 1,4-diradical (T*) as a new C–C bond is formed. The C=O has strengthened in comparison to the C=O in the triplet state (T) and is moving towards the product C=O stretch at 1720 cm^−1^. Intriguingly, the band at 1615 cm^−1^ in thiomaleimide 1 also begins to decay towards the end of the experiment (Fig. 3g), with a slightly longer lifetime >2.5 ns (outside the experimental time window). This decay could be associated with a change in the C=O vibrational mode as the molecule rearranges, but interestingly, is not accompanied by a concurrent change in the electronic absorption in the TEAS. Furthermore, no new band is seen to grow in at 1665 cm^−1^, thus there is no evidence of the formation of the triplet 1,4-diradical (T*) in the intermolecular reaction. This confirms that the intramolecular reaction involves a faster C–C bond formation in comparison to the intermolecular reaction.

## Conclusion

In conclusion, we have shown that the stereochemical outcome of thiomaleimide [2 + 2] photocycloadditions can be switched from *exo* to *endo* by incorporation of an *N*-tether between the two thiomaleimide moieties. This is desirable as it places the sulfur atoms in closer proximity and has the potential to allow these reagents to be employed to introduce disulfide staples photochemically, amongst other prospective applications. The initial biological capability has been demonstrated through applying this reaction on an antibody Fab fragment, which resulted in converting a thiomaleimide conjugate from thiol cleavable to thiol stable, using light. The intramolecular [2 + 2] photocycloaddition reaction is extremely efficient, taking place in just 10 min, in a quantitative yield, with a high quantum yield of 0.23; compared to 0.022 for the intermolecular reaction. Femtosecond time-resolved spectroscopy studies, TEAS and TVAS, were employed to observe the flow of energy between the electronically excited states and its impact on the C=O stretching frequency in both the *exo* and *endo* photocycloaddition reactions. They reveal that an initial S_2_ (π_1_π*) excited state is formed, which then decays to S_1_ (n_1_π*), before ISC to a triplet state (T) in ∼20 ps. The timescales observed in the TVAS support this mechanism and reveal strengthening of the carbonyl bond as the S_1_ (n_1_π*) state evolves into a triplet state, interpreted as reduced repulsion of the parallel electron spins. Finally, the triplet state appears to be a long-lived electronic state for thiomaleimide 1 whereas it is observed to decay for bis-thiomaleimide 4a, proposed to be by C–C bond formation to produce the triplet 1,4-diradical (T*). This accelerated decay is consistent with the conformational control experienced in the intramolecular reaction of bis-thiomaleimide 4a. We propose that such substituted maleimides, which can be readily synthesised and diversified in properties, will offer broad opportunities as photochemically mediated reactive handles; for example, for the formation of disulfide bond staples in peptides and proteins and for controlled crosslinking in polymers.

## Data availability

All experimental and characterisation data in this article are available in the ESI.†

## Author contributions

R. M., V. C., H. H. F. and J. R. B. conceived and designed the project; R. M., V. C. and J. R. B. conceived and designed the synthesis and chemical biology experiments; R. M. performed the synthesis and chemical biology experiments and R. M., V. C. and J. R. B. analysed this data; R. M., M. A. P., V. G. S. and H. H. F. conceived and designed the TEAS and TVAS experiments; M. S. and J. M. W. performed the TEAS and TVAS experiments and R. M., M. A. P., J. M. W., V. G. S. and H. H. F. analysed this data; R. M., M. A. P. and H. H. F. conceived and designed the computational calculations; M. A. P. performed the computational calculations and R. M., M. A. P. and H. H. F. analysed this data; R. M., M. A. P., H. H. F. and J. R. B. co-wrote the paper.

## Conflicts of interest

The authors declare no competing financial interests.

## Supplementary Material

SC-013-D1SC06804K-s001
