# Supplementary material for: Intramolecular thiomaleimide [2 + 2] photocycloadditions: stereoselective control for disulfide stapling and observation of excited state intermediates by transient absorption spectroscopy
Source: Chem Sci. 2022 Jan 28;13(10):2909–18. doi: 10.1039/d1sc06804k (PMC8905992; doi:10.1039/d1sc06804k)
Supplement: SC-013-D1SC06804K-s001 [file SC-013-D1SC06804K-s001.pdf]

Electronic Supplementary Material (ESI) for Chemical Science.
This journal is © The Royal Society of Chemistry 2022

Electronic Supporting Information

Intramolecular thiomaleimide [2+2] photocycloadditions: stereoselective control for disulfide stapling and observation of excited state intermediates by transient absorption spectroscopy

Roshni Malde,[†] Michael A. Parkes,[†] Michael Staniforth,[‡] Jack M. Woolley,[‡] Vasilios G. Stavros,[‡] Vijay Chudasama,[†,] Helen H. Fielding,[†,*] and James R. Baker[†,*]*

† Department of Chemistry, University College London, 20 Gordon Street, London, WC1H 0AJ, UK

‡ Department of Chemistry, University of Warwick, Gibbet Hill Road, Coventry, CV4 7AL, UK

1. Synthetic organic chemistry 4

1.1. General remarks 4

1.2. Synthesis and characterisation 5

Tetrahydrocyclobuta[1,2-*c*:3,4-*c'*]dipyrrole-1,3,4,6(2*H*,5*H*)-tetraone[1] 5

3-Bromo-1-methyl-1*H*-pyrrole-2,5-dione, 12[2] 7

3-(Hexylthio)-1-methyl-1*H*-pyrrole-2,5-dione, 1[3] 9

(3a*S*,3b*S*,6a*R*,6b*R*)-3a,3b-*Bis*(hexylthio)-2,5-dimethyltetrahydrocyclobuta[1,2-*c*:3,4-*c'*]dipyrrole-1,3,4,6(2*H*,5*H*)-tetraone, 2[3] 11

Figure 1: ^{1}H NMR spectrum of photoproduct 2 11

1,1'-(Propane-1,3-diyl)*bis*(3-bromo-1*H*-pyrrole-2,5-dione), 3a 13

1,1'-(Propane-1,3-diyl)*bis*(3-(hexylthio)-1*H*-pyrrole-2,5-dione), 4a 15

(3a*R*,3b*S*,6a*R*,6b*S*)-3a,3b-*Bis*(hexylthio)tetrahydro-2,5-propanocyclobuta[1,2-*c*:3,4-*c'*]dipyrrole-1,3,4,6-tetraone, 5a 17

Figure 2: ^{1}H NMR spectrum of photoproduct 5a 17

1,1'-(Butane-1,4-diyl)*bis*(3-bromo-1*H*-pyrrole-2,5-dione), 3b 19

1,1'-(Butane-1,4-diyl)*bis*(3-(hexylthio)-1*H*-pyrrole-2,5-dione), 4b 21

(3a*R*,3b*S*,6a*R*,6b*S*)-3a,3b-*Bis*(hexylthio)tetrahydro-2,5-butanocyclobuta[1,2-*c*:3,4-*c'*]dipyrrole-1,3,4,6-tetraone, 5b 23

1,1'-(2,2-Dimethylpropane-1,3-diyl)*bis*(3-bromo-1*H*-pyrrole-2,5-dione), 3c 25

1,1'-(2,2-Dimethylpropane-1,3-diyl)*bis*(3-(hexylthio)-1*H*-pyrrole-2,5-dione), 4c 27

(3a*R*,3b*S*,6a*R*,6b*S*)-3a,3b-*Bis*(hexylthio)-8,8-dimethyltetrahydro-2,5-propanocyclobuta[1,2-c:3,4-c']dipyrrole-1,3,4,6-tetraone, 5c 29

1-Butyl-1*H*-pyrrole-2,5-dione, 9[4] 31

1,1'-(Propane-1,3-diyl)*bis*(1*H*-pyrrole-2,5-dione), 10[5] 33

2. Bioconjugation 35

2.1. General remarks 35

Table 1: Components of 12% glycine SDS-PAGE 36

Table 2: LCMS gradient for trastuzumab Fab conjugates. 36

Figure 3: LCMS of trastuzumab Fab 37

2.2. Trastuzumab Fab bioconjugation 38
Preparation of Fab conjugate 6 38
Figure 4: LCMS of conjugate 6 .... *38*
Irradiation of Fab conjugate 6 39
Figure 5: LCMS of conjugate 7 .... *39*
Testing the thiol stability of conjugate 6 with BME 40
Figure 6: LCMS upon treatment of conjugate 6 with BME *40*
Testing the thiol stability of conjugate 6 with EDT 41
Figure 7: LCMS upon treatment of conjugate 6 with EDT *41*
Testing the thiol stability of conjugate 7 with BME 42
Figure 8: LCMS upon treatment of conjugate 7 with BME *42*
Testing the thiol stability of conjugate 7 with EDT 43
Figure 9: LCMS upon treatment of conjugate 7 with EDT *43*

3. *Ab initio* calculations 44
Figure 10: Model chromophore used for ab initio calculations. *44*
Table 3: Optimised structure for the thiomaleimide model chromophore *44*
Table 4: Summary of ab initio calculated energies for the excited state *45*
Figure 11: Molecular orbitals (MOs) in the active space for the model chromophore *45*

4. Quantum yields 46
Figure 12: Graphs used to determine photon flux from the photoconversion of o-nitrobenzaldehyde to o-nitrosobenzoic acid *46*
Figure 13: Graphs used to calculate the quantum yield for thiomaleimide 1 .... *47*

5. TEAS and TVAS experiments 48
Figure 14: The original independently normalised false colour plots of the TEAS and TVAS experiments for bis-thiomaleimide 4a *48*
Table 5: Summary of Instrument Response Function (IRF) values *49*
Figure 15: Decay Associated Spectra (DAS) for the TEAS and TVAS experiments of thiomaleimide 1 and bis-thiomaleimide 4a *49*
Figure 16: The TEAS spectral profile at different time delays for thiomaleimide 1 and bis-thiomaleimide 4a *50*

6. References 51

1. Synthetic organic chemistry

1.1. General remarks

All chemical reagents and solvents were purchased from chemical suppliers and used as received, without any further purification. Reactions were monitored by thin layer chromatography (TLC), using TLC plates pre-coated with silica gel 60 F_{254} on aluminium (Merck KGaA), and detected with UV light (254 nm and 365 nm) or $KMnO_4$ chemical stain. Column chromatography was carried out using a Biotage Isolera using GraceResolv or FlashPure silica flash cartridges. ^{1}H and ^{13}C NMR spectra were recorded in the stated solvent on a Bruker Avance Neo 700 instrument equipped with a 5mm helium-cooled broadband cryoprobe operating at 700 MHz for ^{1}H and 176 MHz for ^{13}C; or a Bruker Avance III 600 instrument equipped with a 5mm helium-cooled $^{1}H/^{13}C$ cryoprobe, operating at 600 MHz for ^{1}H and at 151 MHz for ^{13}C. Chemical shifts (δ) are recorded in parts per million (ppm) and coupling constants (*J*) in Hertz (Hz). The multiplicity of each signal is designated as follows: s (singlet), d (doublet), t (triplet), q (quartet), p (pentet), h (hextet), m (multiplet), or a mixture of these. DEPT, COSY, HSQC, HMBC and NOESY were used to aid the assignment. Infrared spectra were recorded on a Bruker ALPHA FTIR spectrometer with frequencies recorded in reciprocal centimetres (cm^{-1}) and absorptions were characterised as follows: s (sharp), br (broad), m (medium), w (weak). Melting points were taken on an Electrothermal IA9000 series apparatus or a Gallenkamp 5A 6797 apparatus and are uncorrected. All mass spectra were obtained at either the Department of Chemistry, University College London on the one of the following instruments: Agilent 6510 QTOF LC-MS system, Finnigan MAT 900 XP double focusing hybrid (EBqQ) mass spectrometer, Thermo Scientific Orbitrap Q Exactive Plus mass spectrometer, Thermo Scientific TRACE 1310 GC-MS connected to Thermo Scientific ISQ single quadrupole mass spectrometer, Waters LCT Premier XE QTOF mass spectrometer; or obtained at the EPSRC UK National Mass Spectrometry Facility (NMSF), Swansea on one of the following instruments: Thermo Scientific LTQ Orbitrap XL linear ion trap mass spectrometer, Waters Xevo G2-S QTOF mass spectrometer. UV-VIS absorption spectra were recorded on a Shimadzu UV-2600 UV-VIS spectrophotometer using a quartz cuvette with a path length of 1 cm and extinction coefficients are reported to 2 s.f. UV irradiation was performed with a medium pressure 125 W mercury lamp (240-600 nm), purchased from Photochemical Reactors Ltd., with the use of a pyrex immersion well. If the lamp was taken out of use, and then reinstalled, the [2+2] photocycloaddition of maleimide to give tetrahydrocyclobuta[1,2-c:3,4-c']dipyrrole-1,3,4,6(2H,5H)-tetraone was performed as a control reaction,[1] prior to irradiation of any unknown compounds.

1.2. Synthesis and characterisation

Tetrahydrocyclobuta[1,2-*c*:3,4-*c'*]dipyrrole-1,3,4,6(2*H*,5*H*)-tetraone[1]

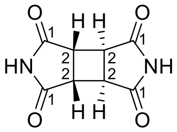

A solution of maleimide (23.5 mg, 0.232 mmol) in MeCN (30 mL) was degassed for 30 min. The solution was then irradiated in pyrex glassware for 1 h whilst stirring at room temperature. The solvent was removed *in vacuo* to afford the title compound as an off-white solid (22.5 mg, 0.116 mmol) in a quantitative yield.

m.p. >250 °C; ^{1}H NMR (700 MHz, DMSO): δ 3.33 (s, 4H, 2-C*H*); ^{13}C NMR (176 MHz, DMSO): δ 177.6 (*C*-1), 42.5 (*C*-2); IR (solid, cm^{-1}): 3073 (br) 2811 (w), 1767 (s), 1694 (s); LRMS (EI+) (m/z, %): 194.0 ([M]$^{+}$, 100), 195.0 ([M+H]$^{+}$, 10).

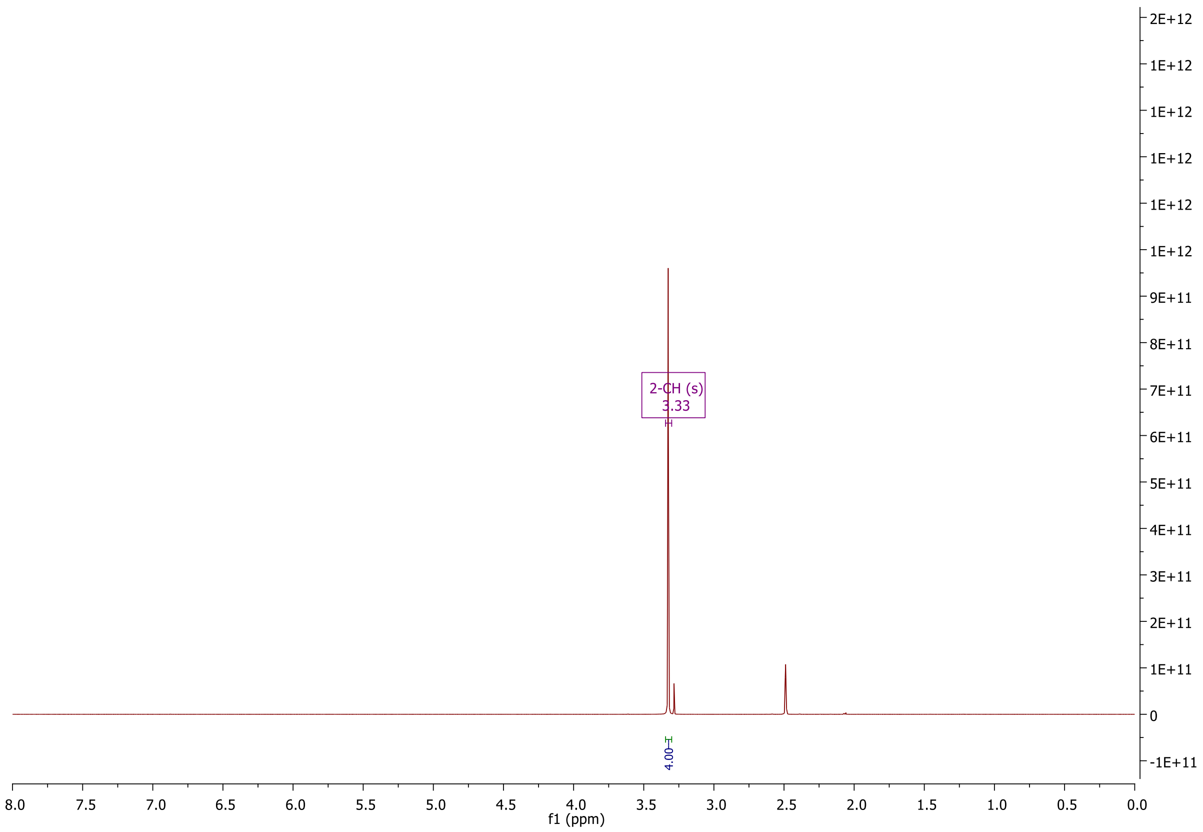
2-CH (s)
3.33
4.00
f1 (ppm)

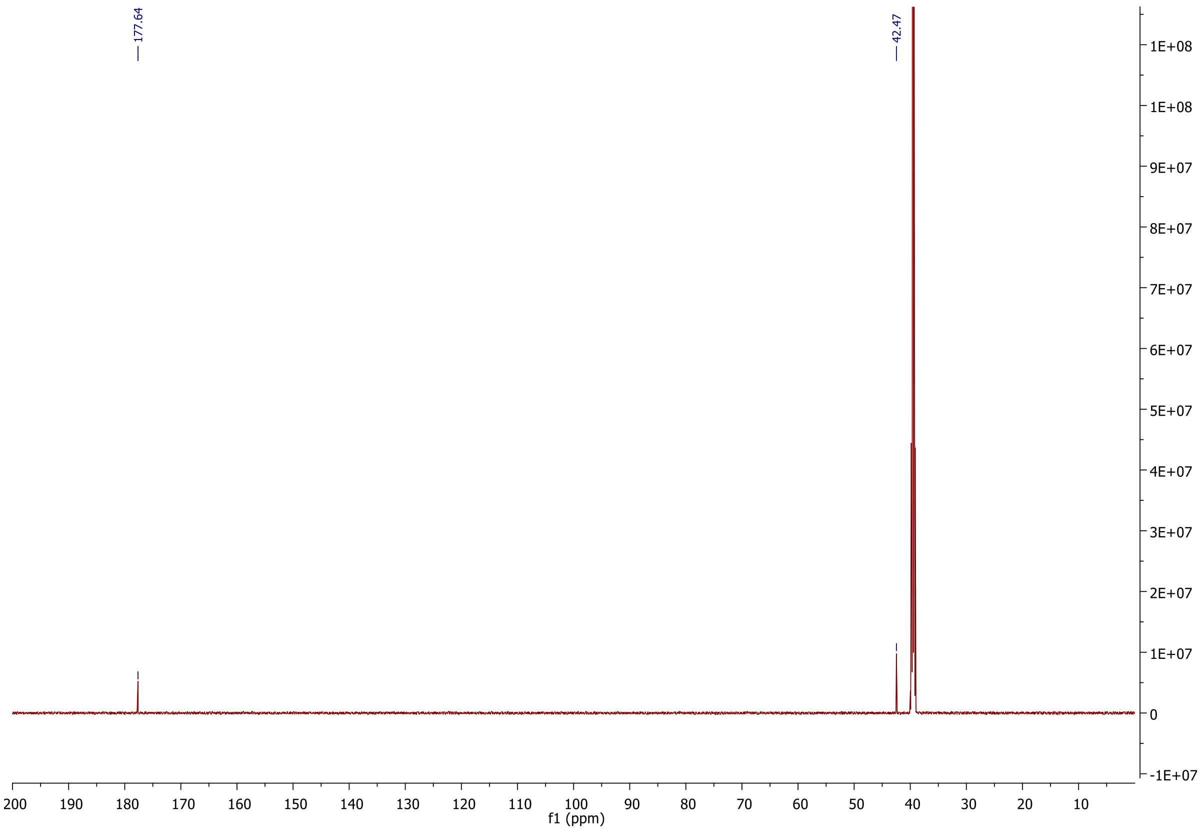
177.64
42.47
f1 (ppm)

3-Bromo-1-methyl-1*H*-pyrrole-2,5-dione, 12[2]

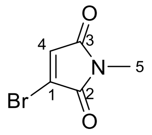

To a solution of *N*-methyl maleimide (556 mg, 5.00 mmol) in MeOH (20 mL), Br_2 (0.564 mL, 11.0 mmol) was added dropwise over 10 min. The reaction mixture was stirred for 8 h at room temperature. The solvent was removed *in vacuo* to give a dark brown oil of the dibromo-*N*-methyl succinimide. The oil was redissolved in THF (20 mL) and triethylamine (0.906 mL, 6.50 mmol) was added. The reaction mixture was further stirred for 18 h at room temperature and the solvent removed *in vacuo*. The crude product was purified by silica flash chromatography (gradient elution of 0-20% EtOAc in cyclohexane) to afford the title compound as an off-white solid (805 mg, 4.24 mmol) in an 85% yield.

m.p. 86-88 °C (lit. 77-79 °C)[2]; ^{1}H NMR (700 MHz, $CDCl_3$): δ 6.87 (s, 1H, 4-C*H*), 3.07 (s, 3H, 5-CH_3); ^{13}C NMR (176 MHz, $CDCl_3$): δ 168.7 (*C*-2/3), 165.5 (*C*-2/3), 132.1 (*C*-4), 131.5 (*C*-1), 24.8 (*C*-5); IR (solid, cm^{-1}): 3106 (w), 2945 (w), 1698 (s), 1586 (m); LRMS (ASAP+) (m/z, %): 190.0 ([^{79}BrM+H]$^+$, 95), 191.9 ([^{81}BrM+H]$^+$, 100); UV (MeCN): ε_{300} = 330 cm^{-1} M^{-1}.

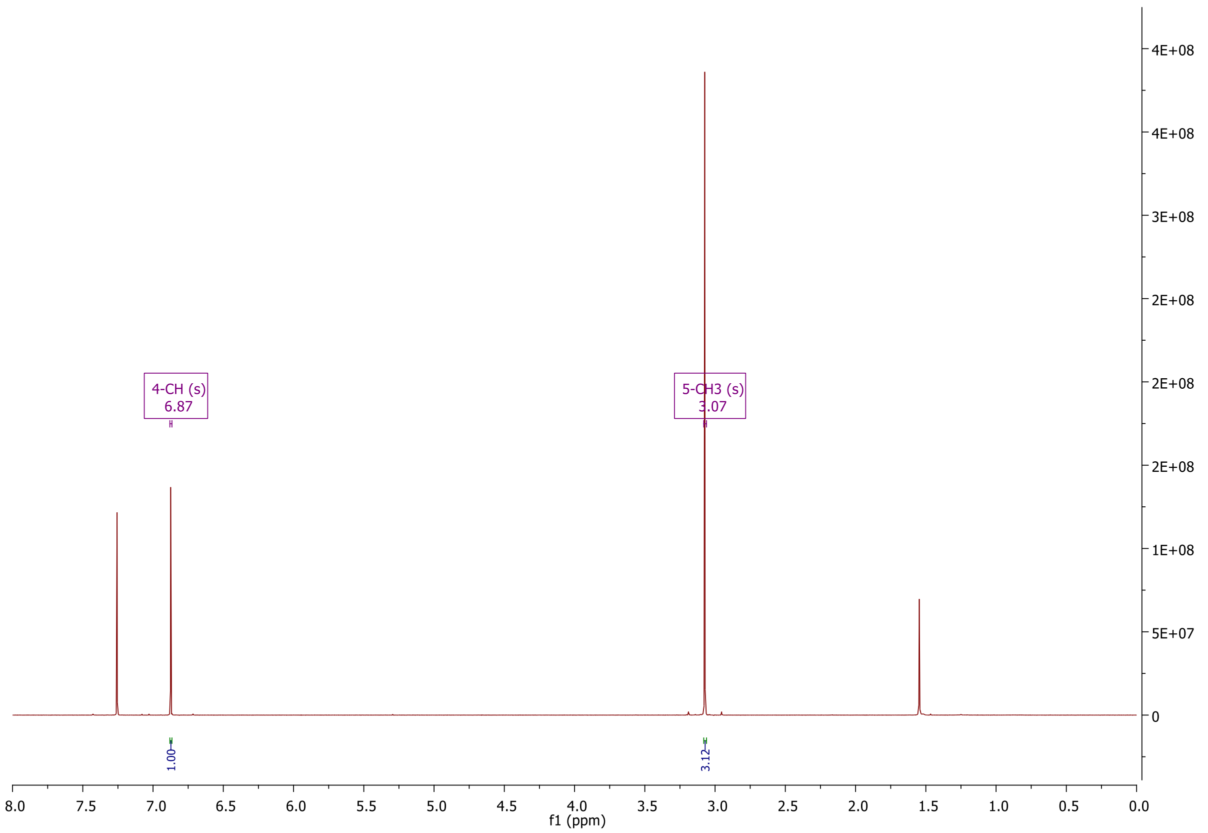
4-CH (s)
6.87
5-CH3 (s)
3.07
1.00
3.12
f1 (ppm)

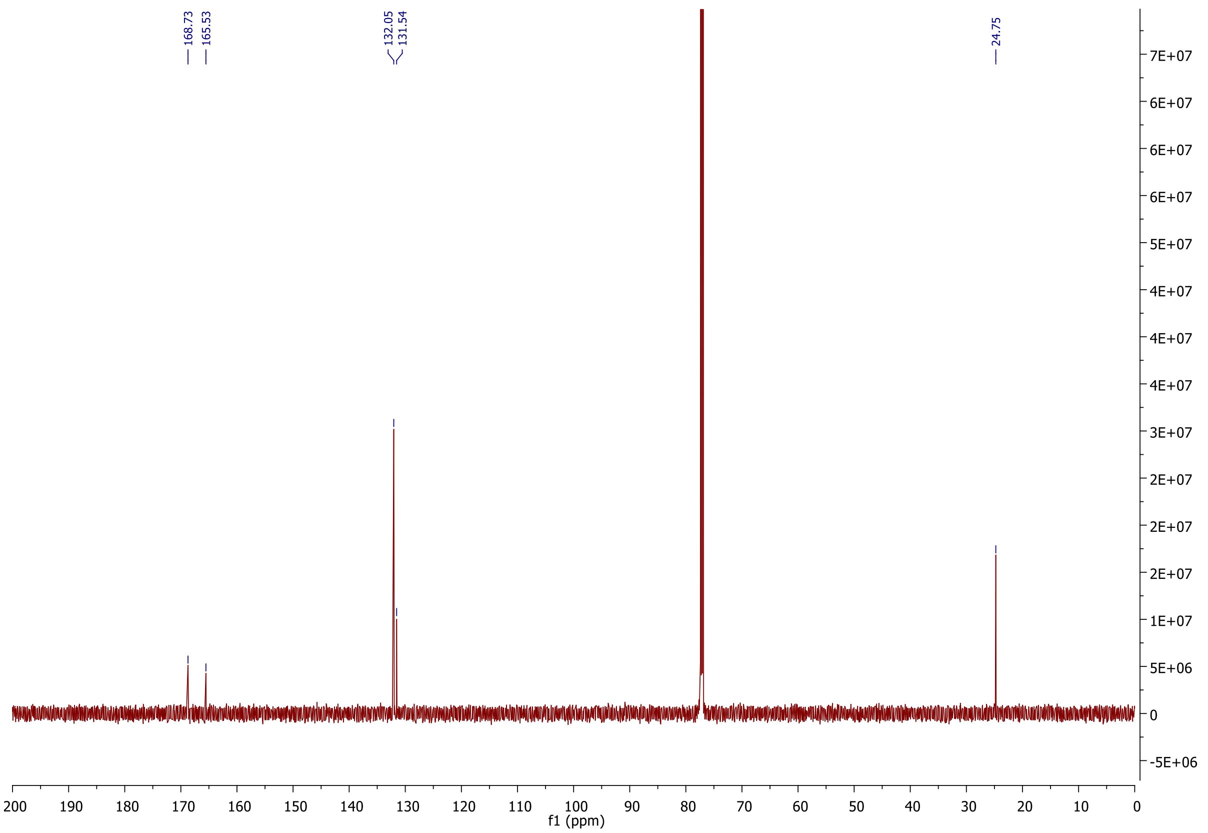
168.73
165.53
132.05
131.54
24.75
f1 (ppm)

3-(Hexylthio)-1-methyl-1*H*-pyrrole-2,5-dione, 1[3]

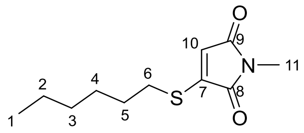

To a solution of **12** (66.5 mg, 0.350 mmol) in DCM (30 mL), triethylamine (53.6 μL, 0.385 mmol) was added, followed by hexanethiol (54.3 μL, 0.385 mmol). The reaction mixture was stirred for 30 min at room temperature. The solvent was removed *in vacuo*. The crude product was then purified by silica flash chromatography (gradient elution of 0-20% EtOAc in cyclohexane) to afford the title compound as an off-white solid (77.2 mg, 0.340 mmol) in a 97% yield.

m.p. 62-65 °C (lit. 62-65 °C)[3]; ^{1}H NMR (700 MHz, $CDCl_3$): δ 6.04 (s, 1H, 10-C*H*), 3.02 (s, 3H, 11-CH_3), 2.90 (t, *J* = 7.4 Hz, 2H, 6-CH_2), 1.74 (p, *J* = 7.4 Hz, 2H, 5-CH_2), 1.45 (p, *J* = 7.4 Hz, 2H, 4-CH_2), 1.34 – 1.28 (m, 4H, 2-CH_2 and 3-CH_2), 0.90 (t, *J* = 6.6 Hz, 3H, 1-CH_3); ^{13}C NMR (176 MHz, $CDCl_3$): δ 170.0 (*C*-8/9), 168.3 (*C*-8/9), 151.9 (*C*-7), 117.3 (*C*-10), 32.0 (*C*-6), 31.3 (*C*-3), 28.7 (*C*-4), 27.8 (*C*-5), 24.1 (*C*-11), 22.6 (*C*-2), 14.1 (*C*-1); IR (solid, cm^{-1}): 3079 (w), 2929 (m), 1692 (s), 1557 (m); LRMS (ASAP+) (m/z, %): 228.1 ($[M+H]^+$, 100); UV (MeCN): ε_{354} = 3600 cm^{-1} M^{-1}.

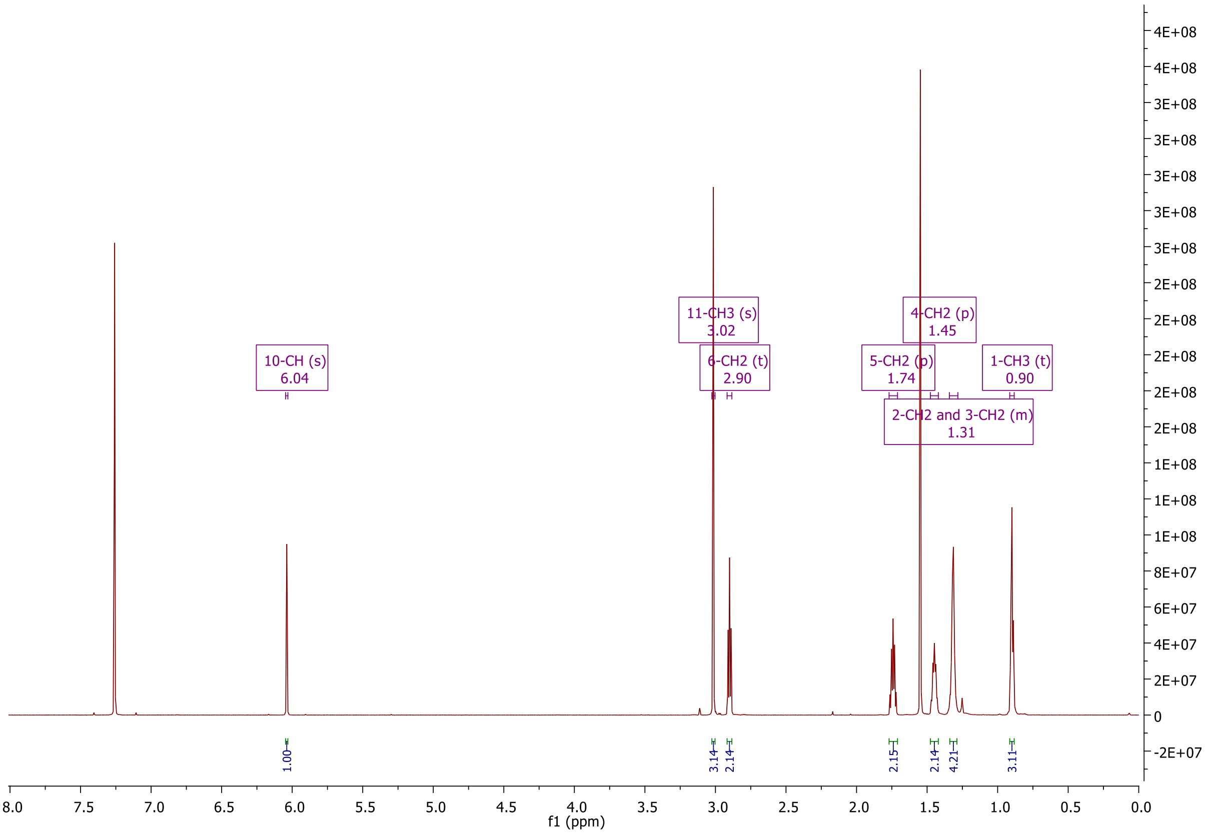
10-CH (s)
6.04
11-CH3 (s)
3.02
6-CH2 (t)
2.90
4-CH2 (p)
1.45
5-CH2 (p)
1.74
1-CH3 (t)
0.90
2-CH2 and 3-CH2 (m)
1.31
1.00
3.14
2.14
2.15
2.14
4.21
3.11
f1 (ppm)

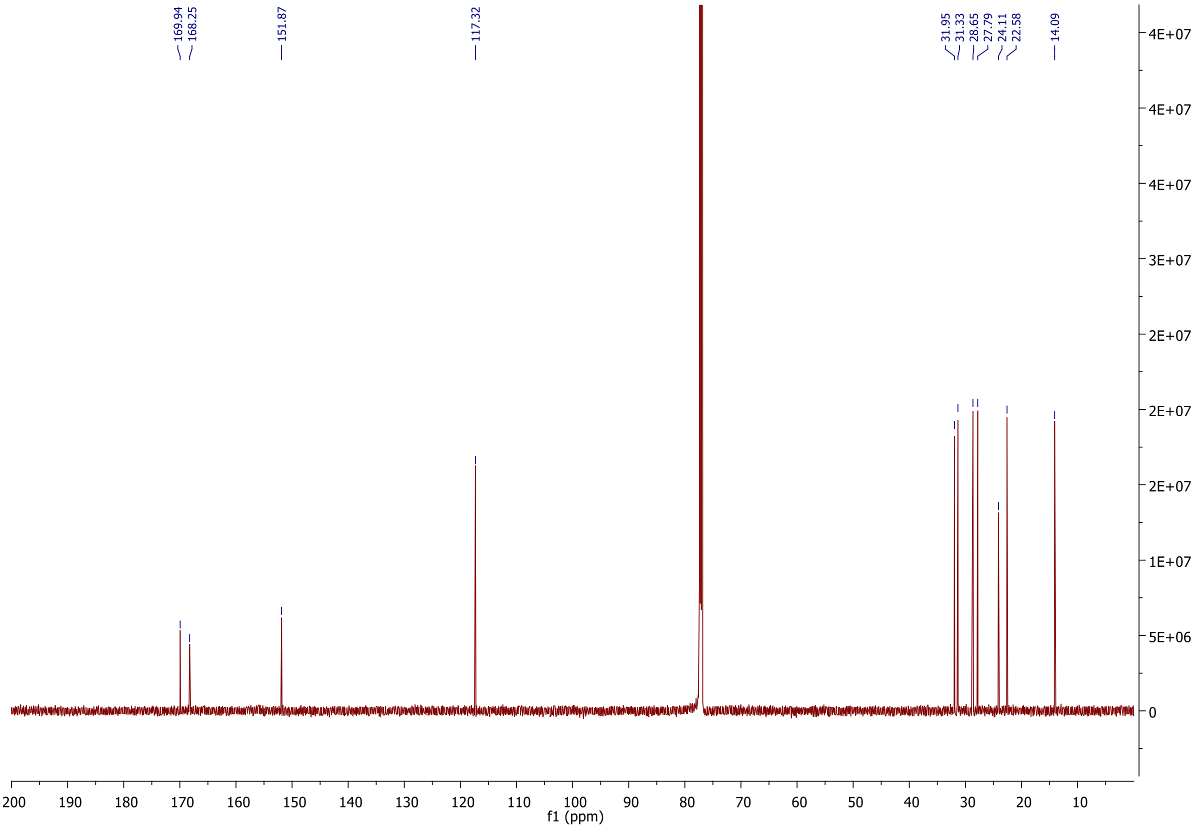
169.94
168.25
151.87
117.32
31.95
31.33
28.65
27.79
24.11
22.58
14.09
f1 (ppm)

(3a*S*,3b*S*,6a*R*,6b*R*)-3a,3b-*Bis*(hexylthio)-2,5-dimethyltetrahydrocyclobuta[1,2-*c*:3,4-*c'*]dipyrrole-1,3,4,6(2*H*,5*H*)-tetraone, 2[3]

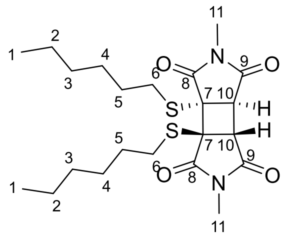

A 5 mM solution of **1** (34.1 mg, 0.150 mmol) in MeCN (30 mL) was degassed for 30 min. The solution was then irradiated in pyrex glassware for 10 min whilst stirring at room temperature. The solvent was removed *in vacuo* to afford the title compound as a yellow solid (34.1 mg, 0.0750 mmol) in a quantitative yield.

m.p. 103-106 °C (lit. 104-106 °C)[3]; ^{1}H NMR (700 MHz, $CDCl_3$): δ 3.15 (dt, *J* = 10.6, 7.4 Hz, 2H, 6-C*H*H), 3.11 (s, 6H, 11-C*H*$_3$), 2.98 (s, 2H, 10-C*H*), 2.84 (dt, *J* = 10.6, 7.4 Hz, 2H, 6-CH*H*), 1.50 (p, *J* = 7.4 Hz, 4H, 5-C*H*$_2$), 1.34 (p, *J* = 7.4 Hz, 4H, 4-C*H*$_2$), 1.30 – 1.23 (m, 8H, 2-C*H*$_2$ and 3-C*H*$_2$), 0.87 (t, *J* = 6.7 Hz, 6H, 1-C*H*$_3$); ^{13}C NMR (176 MHz, $CDCl_3$): δ 173.2 (*C*-8/9), 173.0 (*C*-8/9), 55.4 (*C*-7), 44.6 (*C*-10), 31.4 (*C*-3), 31.2 (*C*-6), 28.71 (*C*-5), 28.6 (*C*-4), 25.9 (*C*-11), 22.6 (*C*-2), 14.2 (*C*-1); IR (solid, cm^{-1}): 2926 (m), 1719 (s); LRMS (ASAP+) (m/z, %): 455.2 ($[M+H]^+$, 100).

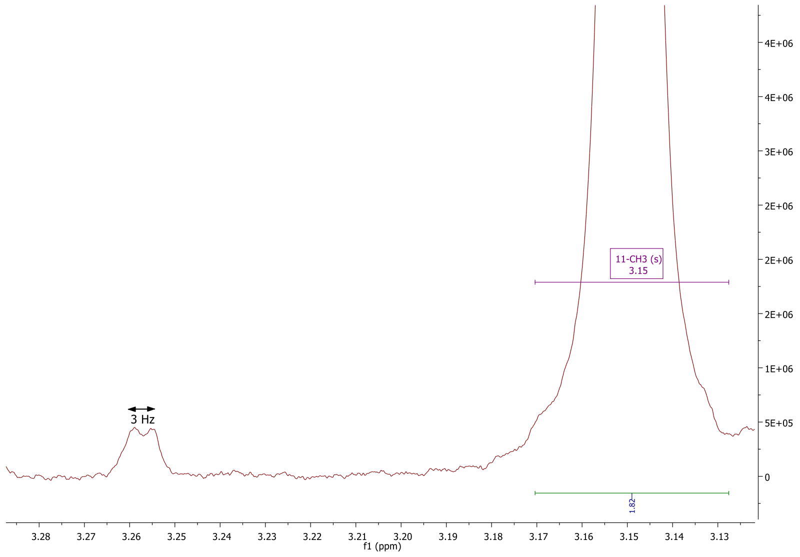

Figure 1: *^{1}H NMR spectrum of photoproduct* ***2*** *showing the cyclobutane 10-CH satellite peak.*

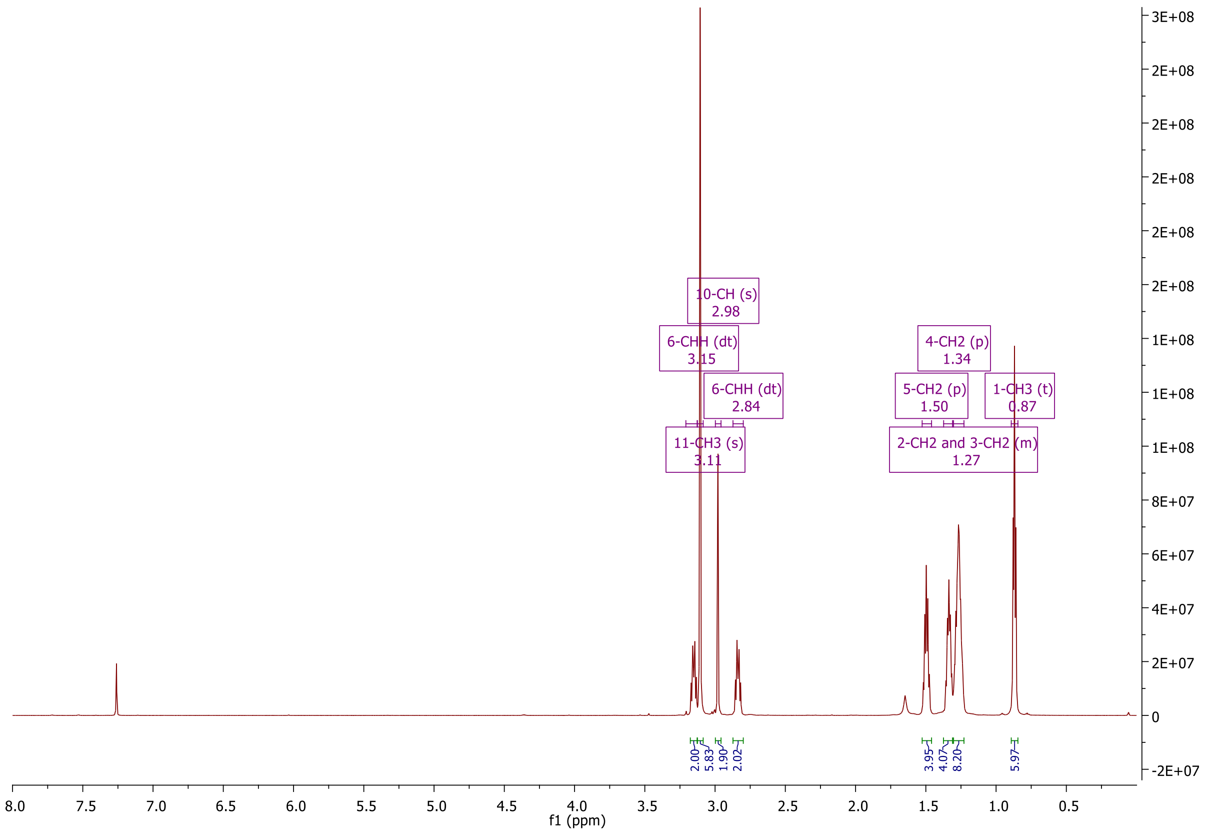
10-CH (s)
2.98
6-CHH (dt)
3.15
6-CHH (dt)
2.84
11-CH3 (s)
3.11
4-CH2 (p)
1.34
5-CH2 (p)
1.50
1-CH3 (t)
0.87
2-CH2 and 3-CH2 (m)
1.27
2.00
5.83
1.90
2.02
3.95
4.07
8.20
5.97
f1 (ppm)

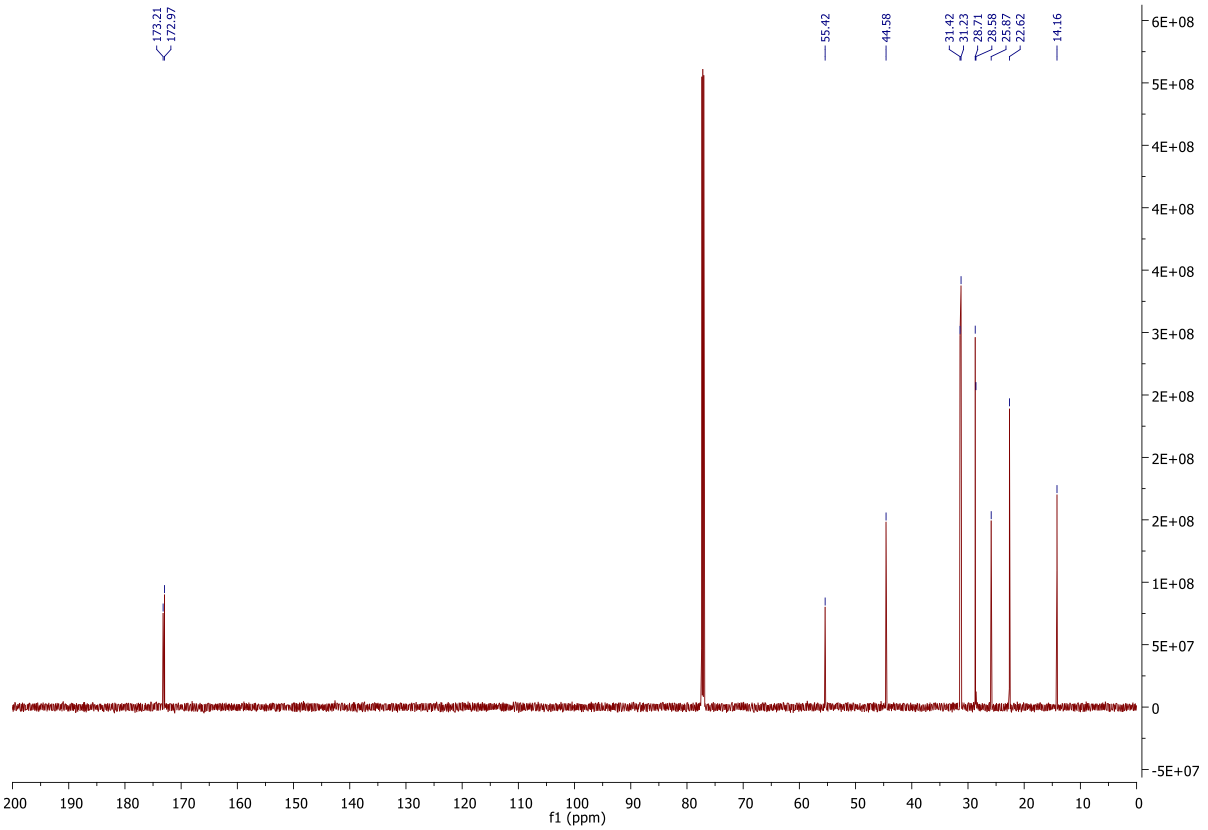
173.21
172.97
55.42
44.58
31.42
31.23
28.71
28.58
25.87
22.62
14.16
f1 (ppm)

1,1'-(Propane-1,3-diyl)*bis*(3-bromo-1*H*-pyrrole-2,5-dione), 3a

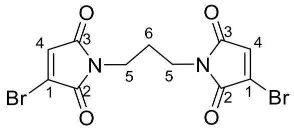

To a solution of 3-bromomaleic anhydride (0.929 mL, 10.0 mmol) in acetic acid (10 mL), 1,3-diaminopropane (0.210 mL, 2.50 mmol) was added and the reaction mixture was heated to reflux for 3 h. The reaction mixture was cooled and the solvent was then removed *in vacuo*, azeotroping with toluene (3 x 10 mL). The product was separated between EtOAc (20 mL) and saturated $NaHCO_3$ solution (20 mL). The organic layer was further washed with brine (20 mL), dried over $MgSO_4$, and the solvent removed *in vacuo*. The crude product was purified by silica flash chromatography (gradient elution of 0-60% EtOAc in cyclohexane) to afford the title compound as an off-white solid (514 mg, 1.31 mmol) in a 52% yield.

m.p. 134-137 °C; ^{1}H NMR (700 MHz, $CDCl_3$): δ 6.88 (s, 2H, 4-C*H*), 3.59 (t, *J* = 7.1 Hz, 4H, 5-C*H₂*), 1.96 (p, *J* = 7.1 Hz, 2H, 6-C*H₂*); ^{13}C NMR (151 MHz, $CDCl_3$): δ 168.5 (*C*-2/3), 165.3 (*C*-2/3), 132.0 (*C*-4), 131.6 (*C*-1), 36.3 (*C*-5), 27.4 (*C*-6) ; IR (solid, cm^{-1}): 3104 (m), 2937 (w), 1704 (s), 1581 (m); LRMS (ASAP+) (m/z, %): 390.9 ([^{79}Br^{79}BrM+H]$^+$, 50), 392.9 ([^{79}Br^{81}BrM+H]$^+$, 100), 394.9 ([^{81}Br^{81}BrM+H]$^+$, 50); HRMS: exact mass calculated for [^{79}Br^{79}BrM+H]$^+$, [$C_{11}H_9{}^{79}Br_2N_2O_4$]$^+$ 390.8929, found 390.8927.

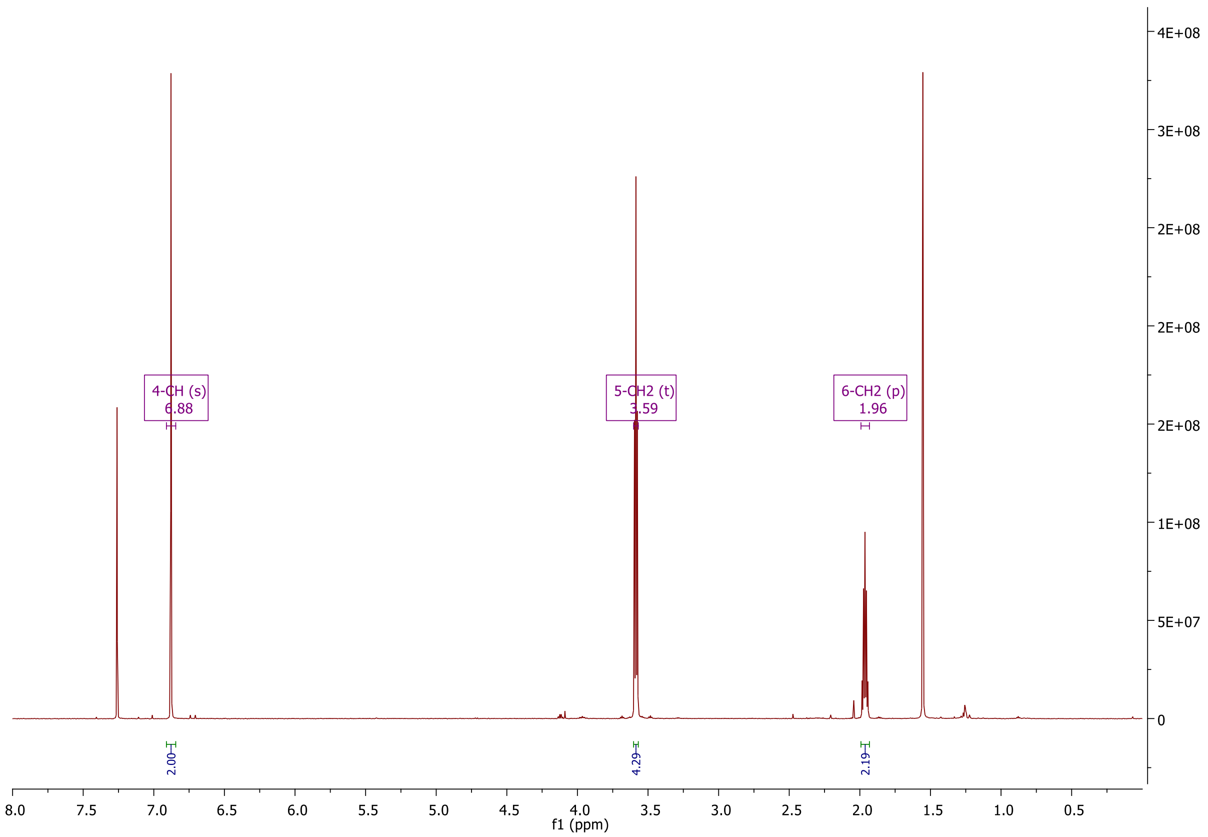
4-CH (s)
6.88
5-CH2 (t)
3.59
6-CH2 (p)
1.96
2.00
4.29
2.19
f1 (ppm)

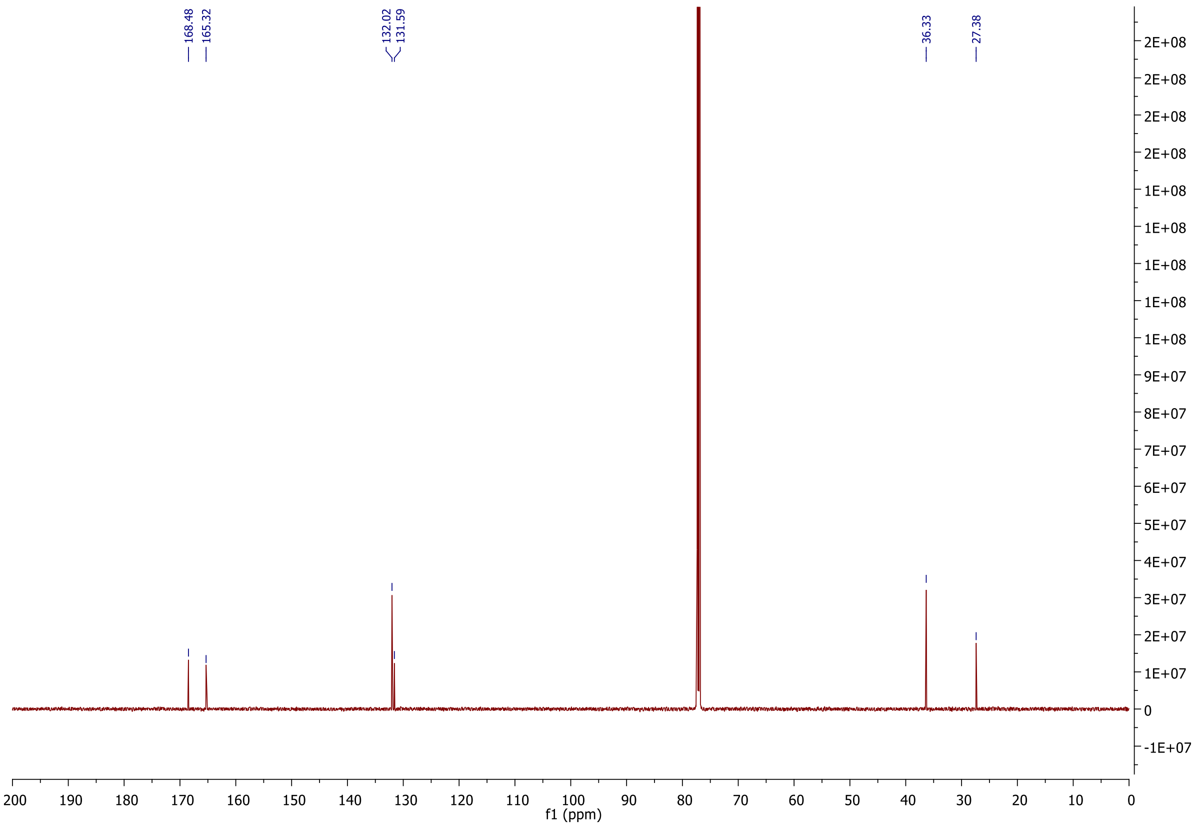
168.48
165.32
132.02
131.59
36.33
27.38
f1 (ppm)

1,1'-(Propane-1,3-diyl)*bis*(3-(hexylthio)-1*H*-pyrrole-2,5-dione), 4a

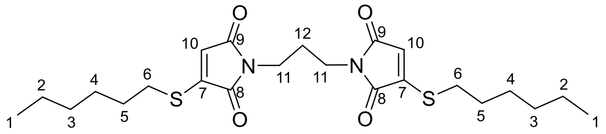

To a solution of **3a** (468 mg, 1.19 mmol) and NaOAc (195 mg, 2.38 mmol) in MeOH (50 mL), hexanethiol (0.336 mL, 2.38 mmol) was added and the reaction mixture was stirred for 30 min at room temperature. The solvent was removed *in vacuo*. The crude product was then purified by silica flash chromatography (gradient elution of 0-40% EtOAc in cyclohexane) to afford the title compound as a yellow solid (322 mg, 0.690 mmol) in a 58% yield.

m.p. 109-112 °C; ^{1}H NMR (600 MHz, $CDCl_3$): δ 6.02 (s, 2H, 10-C*H*), 3.53 (t, *J* = 7.2 Hz, 4H, 11-C*H₂*), 2.89 (t, *J* = 7.4 Hz, 4H, 6-C*H₂*), 1.91 (p, *J* = 7.2 Hz, 2H, 12-C*H₂*), 1.74 (p, *J* = 7.3, 4H, 5-C*H₂*), 1.45 (p, *J* = 7.3 Hz, 4H, 4-C*H₂*), 1.34 – 1.29 (m, 8H, 2-C*H₂* and 3-C*H₂*), 0.90 (t, *J* = 6.8 Hz, 6H, 1-C*H₃*); ^{13}C NMR (151 MHz, $CDCl_3$): δ 169.7 (*C*-8/9), 168.0 (*C*-8/9), 151.9 (*C*-7), 117.2 (*C*-10), 35.8 (*C*-11), 32.0 (*C*-6), 31.3 (*C*-3), 28.7 (*C*-4), 27.8 (*C*-5), 27.7 (*C*-12), 22.6 (*C*-2), 14.1 (*C*-1); IR (solid, cm^{-1}): 3078 (w), 2927 (m), 1693 (s), 1557 (m); LRMS (NS+) (m/z, %): 467.2 ($[M+H]^+$, 100), 484.2 ($[M+NH_4]^+$, 91), 489.2 ($[M+Na]^+$, 84), 499.2 ($[M+H+CH_3OH]^+$, 6]; HRMS: exact mass calculated for $[M+H]^+$, $[C_{23}H_{35}N_2O_4S_2]^+$ 467.2033, found 467.2025; UV (MeCN): ε_{354} = 6600 cm^{-1} M^{-1}.

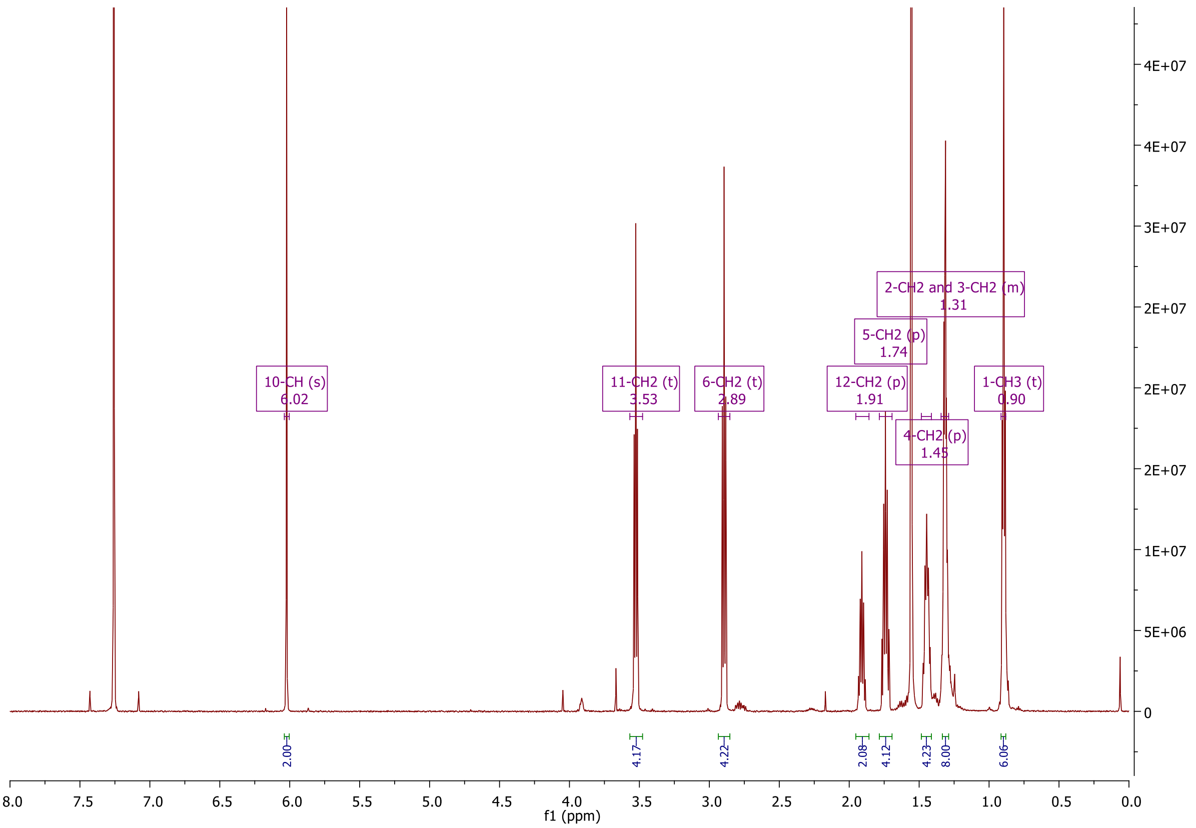
10-CH (s)
6.02
11-CH2 (t)
3.53
6-CH2 (t)
2.89
12-CH2 (p)
1.91
5-CH2 (p)
1.74
2-CH2 and 3-CH2 (m)
1.31
4-CH2 (p)
1.45
1-CH3 (t)
0.90
2.00
4.17
4.22
2.08
4.12
4.23
8.00
6.06
f1 (ppm)

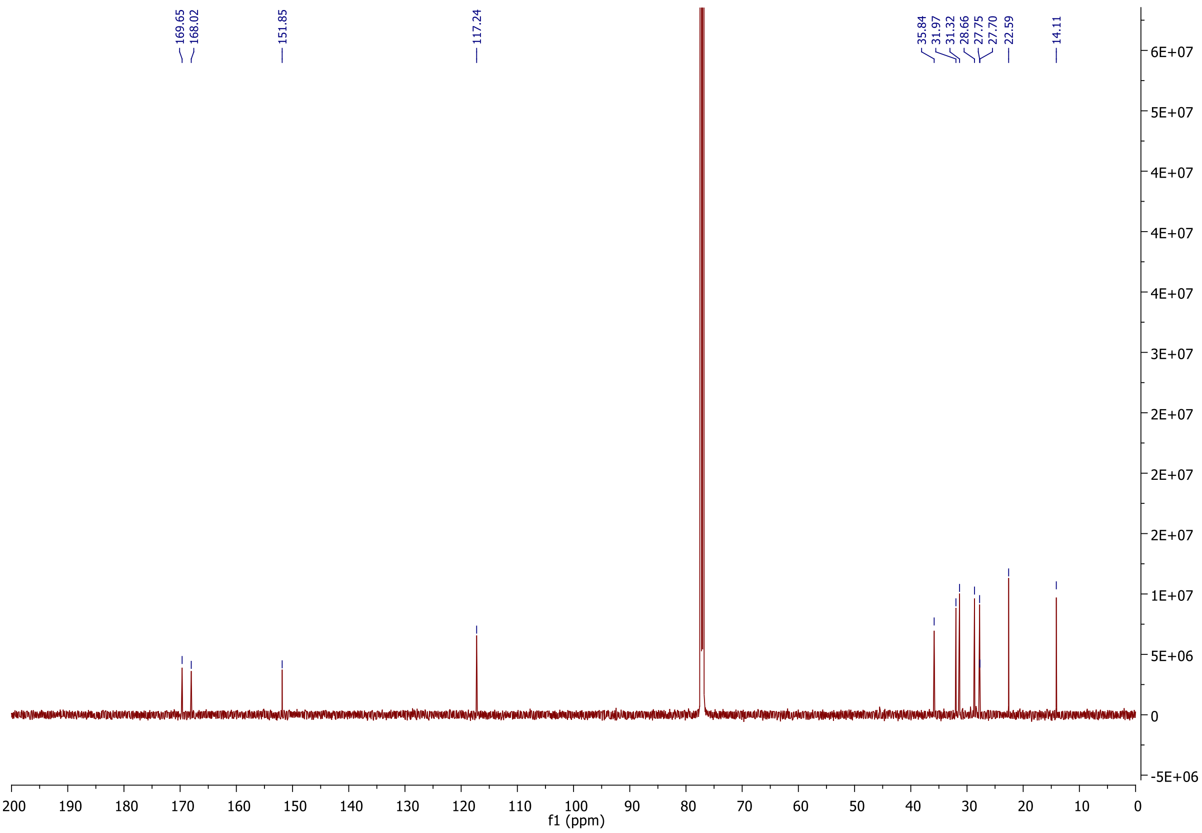
169.65
168.02
151.85
117.24
35.84
31.97
31.32
28.66
27.75
27.70
22.59
14.11
f1 (ppm)

(3a*R*,3b*S*,6a*R*,6b*S*)-3a,3b-*Bis*(hexylthio)tetrahydro-2,5-propanocyclobuta[1,2-*c*:3,4-*c'*]dipyrrole-1,3,4,6-tetraone, 5a

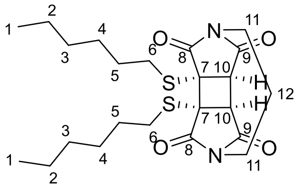

A 2.5 mM solution of **4a** (35.0 mg, 0.0750 mmol) in MeCN (30 mL) was degassed with argon for 30 min. The solution was then irradiated in pyrex glassware for 10 min whilst stirring at room temperature. The solvent was removed *in vacuo* to afford the title compound as a yellow solid (35.0 mg, 0.0750 mmol) in a quantitative yield.

m.p. 148-152 °C; ^{1}H NMR (600 MHz, $CDCl_3$): δ 3.96-3.87 (m, 4H, 11-C*H₂*), 3.67 (s, 2H, 10-C*H*), 2.95 – 2.87 (m, 2H, 6-C*H*H), 2.80 – 2.74 (m, 2H, 6-CH*H*), 2.33 – 2.21 (m, 2H, 12-CH_2), 1.64 – 1.56 (m, 4H, 5-CH_2), 1.42 – 1.36 (m, 4H, 4-CH_2), 1.32 – 1.26 (m, 8H, 2-CH_2 and 3-CH_2), 0.89 (t, *J* = 6.9 Hz, 6H, 1-CH_3); ^{13}C NMR (151 MHz, $CDCl_3$): δ 172.4 (*C*-8/9), 171.7 (*C*-8/9), 58.2 (*C*-7), 45.6 (*C*-10), 41.2 (*C*-11), 31.4 (*C*-6) , 30.8 (*C*-3), 29.4 (*C*-4), 28.6 (*C*-5), 22.6 (*C*-12), 20.6 (*C*-2), 14.1 (*C*-1); IR (solid, cm^{-1}): 2922 (m), 1718 (s); LRMS (NS+) (m/z, %): 467.2 ([M+H]$^+$, 25), 484.2 ([M+NH_4]$^+$, 92), 489.2 ([M+Na]$^+$, 90), 499.2 ([M+H+CH_3OH]$^+$, 58); HRMS: exact mass calculated for [M+H], [$C_{23}H_{35}N_2O_4S_2$] 467.2033, found 467.2030.

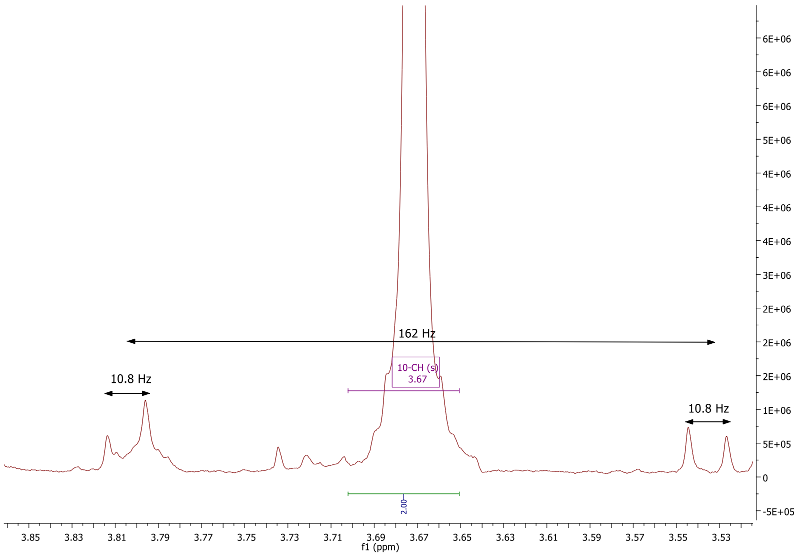

Figure 2: *^{1}H NMR spectrum of photoproduct* ***5a*** *showing the cyclobutane 10-CH proton peak and corresponding satellite peak.*

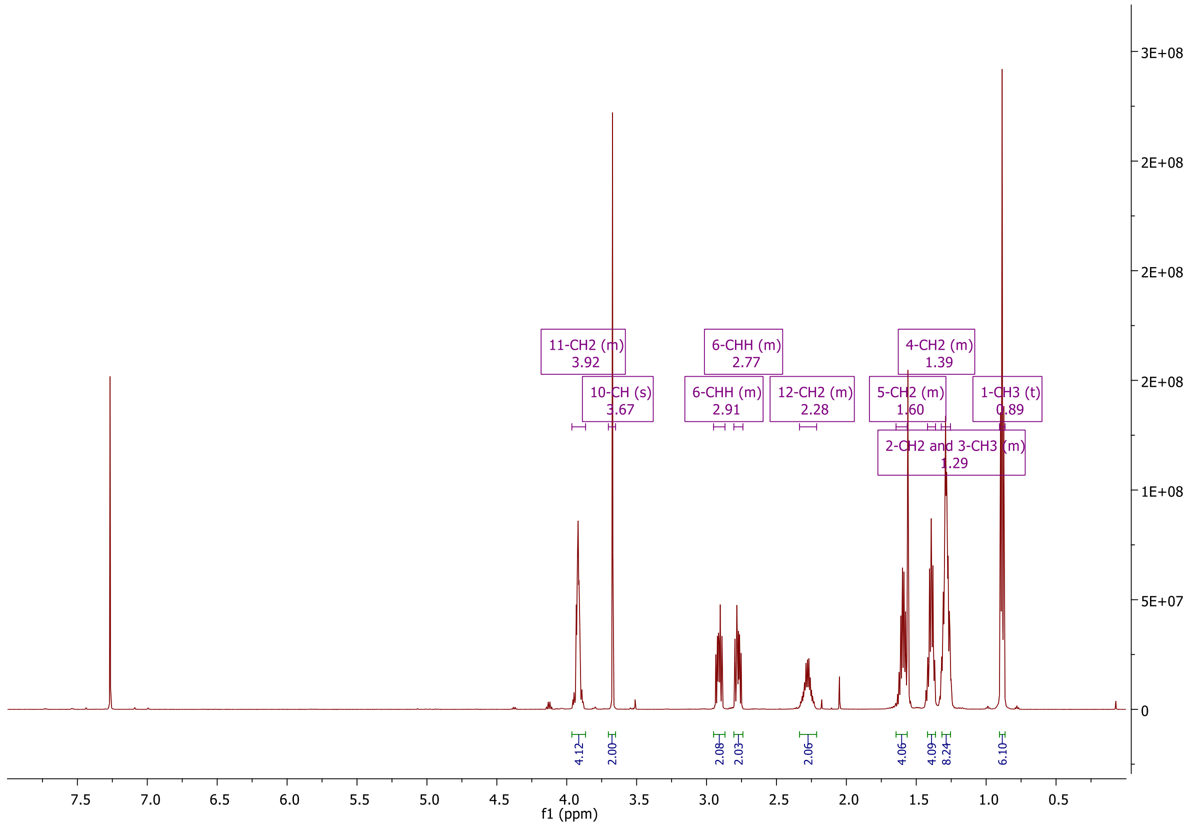
11-CH2 (m)
3.92
10-CH (s)
3.67
6-CHH (m)
2.77
6-CHH (m)
2.91
12-CH2 (m)
2.28
4-CH2 (m)
1.39
5-CH2 (m)
1.60
1-CH3 (t)
0.89
2-CH2 and 3-CH3 (m)
1.29
4.12
2.00
2.08
2.03
2.06
4.06
4.09
8.24
6.10
f1 (ppm)

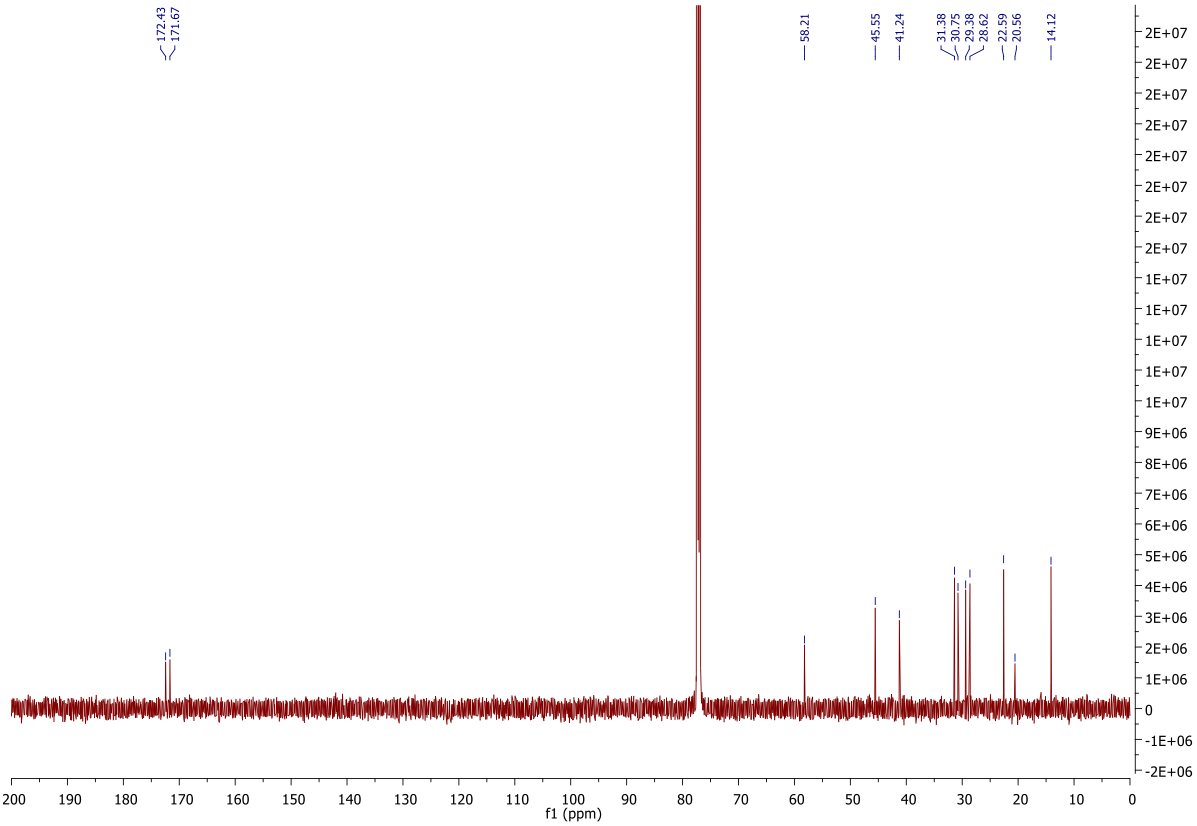
172.43
171.67
58.21
45.55
41.24
31.38
30.75
29.38
28.62
22.59
20.56
14.12
f1 (ppm)

1,1'-(Butane-1,4-diyl)*bis*(3-bromo-1*H*-pyrrole-2,5-dione), 3b

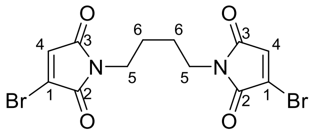

To a solution of 3-bromomaleic anhydride (0.557 mL, 6.00 mmol) in acetic acid (6 mL), 1,4-diaminobutane (0.151 mL, 1.50 mmol) was added and the reaction mixture was heated to reflux for 3 h. The reaction mixture was cooled and the solvent was then removed *in vacuo*, azeotroping with toluene (3 x 10 mL). The product was separated between EtOAc (20 mL) and saturated $NaHCO_3$ solution (20 mL). The organic layer was further washed with brine (20 mL), dried over $MgSO_4$, and the solvent removed *in vacuo*. The crude product was purified by silica flash chromatography (gradient elution of 0-60% EtOAc in cyclohexane) to afford the title compound as a white solid (227 mg, 0.559 mmol) in a 37% yield.

m.p. 174-178 °C; ^{1}H NMR (600 MHz, $CDCl_3$): δ 6.86 (s, 2H, 4-C*H*), 3.60 – 3.56 (m, 4H, 5-C*H$_2$*), 1.60 – 1.57 (m, 4H, 6-C*H$_2$*); ^{13}C NMR (151 MHz, $CDCl_3$): δ 168.6 (*C*-2/3), 165.4 (*C*-2/3), 131.9 (*C*-4), 131.5 (*C*-1), 38.2 (*C*-5), 25.8 (*C*-6); IR (solid, cm^{-1}): 3086 (w), 2921 (w), 1700 (s), 1585 (m); LRMS (NS+) (m/z, %): 404.9 ([^{79}Br^{79}BrM+H]$^+$, 10), 406.9 ([^{79}Br^{81}BrM+H]$^+$, 21), 408.9 ([^{81}Br^{81}BrM+H]$^+$, 10), 428.9 ([^{79}Br^{81}BrM+Na]$^+$, 95), 460.9 ([^{79}Br^{81}BrM+Na+CH_3OH]$^+$, 100); HRMS: exact mass calculated for [^{79}Br^{79}BrM+H]$^+$, [$C_{12}H_{11}{}^{79}Br_2N_2O_4$]$^+$ 404.9080, found 404.9083.

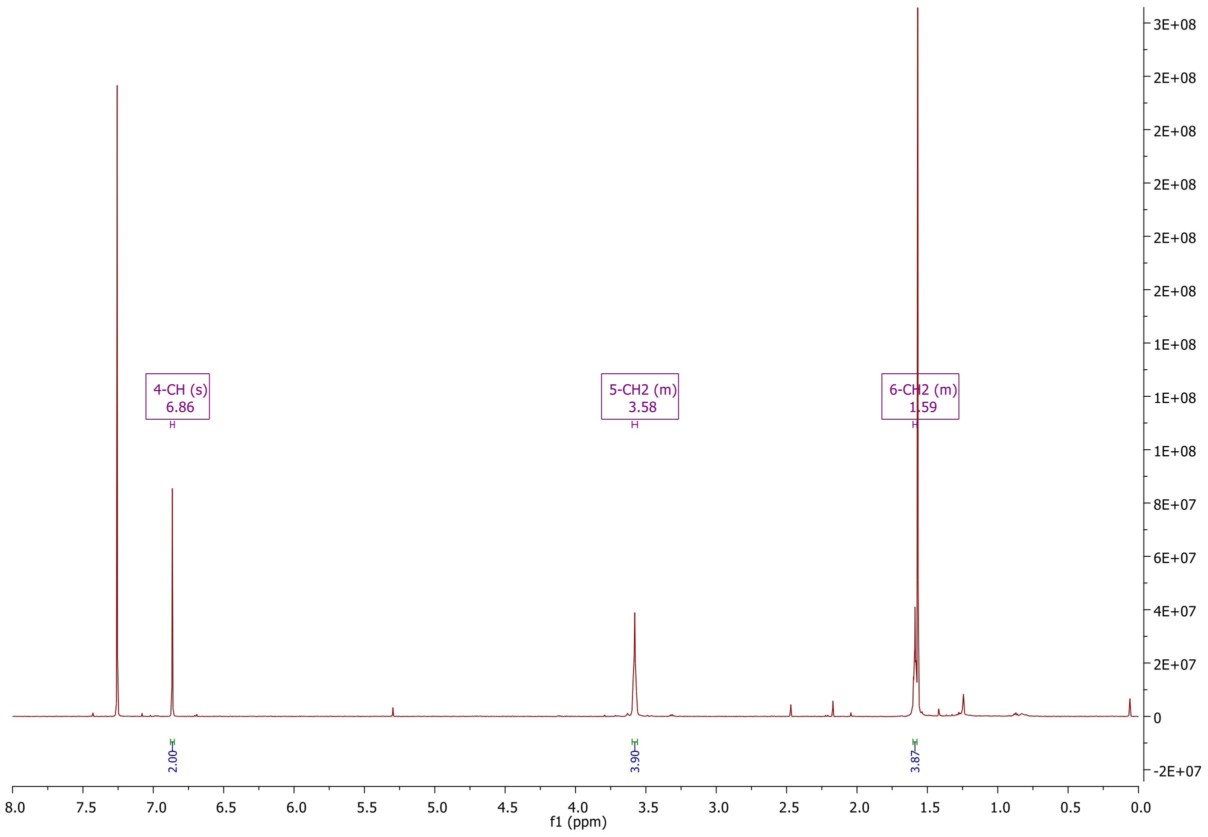
4-CH (s)
6.86
5-CH2 (m)
3.58
6-CH2 (m)
1.59
2.00
3.90
3.87
f1 (ppm)

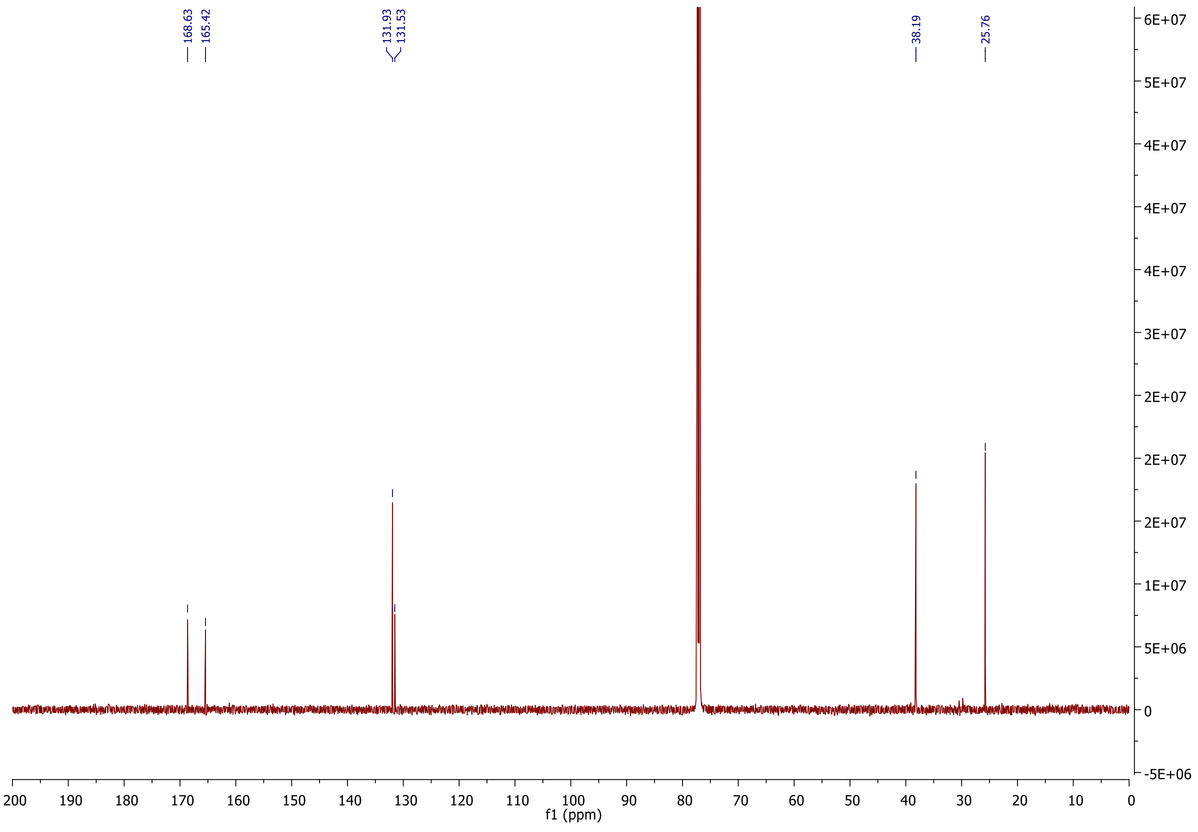
168.63
165.42
131.93
131.53
38.19
25.76
f1 (ppm)

1,1'-(Butane-1,4-diyl)*bis*(3-(hexylthio)-1*H*-pyrrole-2,5-dione), 4b

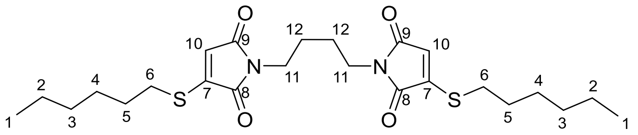

To a solution of **3b** (468 mg, 1.19 mmol) in DCM (30 mL), triethylamine (0.151 mL, 1.08 mmol) was added, followed by hexanethiol (0.152 mL, 1.08 mmol). The reaction mixture was stirred for 30 min at room temperature. The solvent was removed *in vacuo*. The crude product was then purified by silica flash chromatography (gradient elution of 0-40% EtOAc in cyclohexane) to afford the title compound as a yellow solid (186 mg, 0.388 mmol) in a 72% yield.

m.p. 138-142 °C; ^{1}H NMR: (600 MHz, $CDCl_3$) δ 6.02 (s, 2H, 10-C*H*), 3.55-3.50 (m, 4H, 11-CH_2), 2.90 (t, *J* = 7.3 Hz, 4H, 6-CH_2), 1.75 (p, *J* = 7.4 Hz, 4H, 5-CH_2), 1.60-1.57 (m, 4H, 12-CH_2), 1.46 (p, *J* = 7.4 Hz, 4H, 4-CH_2), 1.34 – 1.30 (m, 8H, 2-CH_2 and 3-CH_2), 0.91 (t, *J* = 6.8 Hz, 6H, 1-CH_3); ^{13}C NMR (151 MHz, $CDCl_3$): δ 169.8 (*C*-8/9), 168.1 (*C*-8/9), 151.7 (*C*-7), 117.2 (*C*-10), 37.6 (*C*-11), 31.9 (*C*-6), 31.3 (*C*-3), 28.6 (*C*-4), 27.8 (*C*-5), 26.0 (*C*-12), 22.6 (*C*-2), 14.1 (*C*-1); IR (solid, cm^{-1}): 3074 (w), 2923 (m), 1691 (s), 1555 (m); LRMS (NS+) (m/z, %): 481.2 ($[M+H]^+$, 100), 498.2 ($[M+NH_4]^+$, 83); HRMS: exact mass calculated for $[M+H]^+$, $[C_{24}H_{37}N_2O_4S_2]^+$ 481.2189, found 481.2181; UV (DCM): ε_{354} = 5200 cm^{-1} M^{-1}.

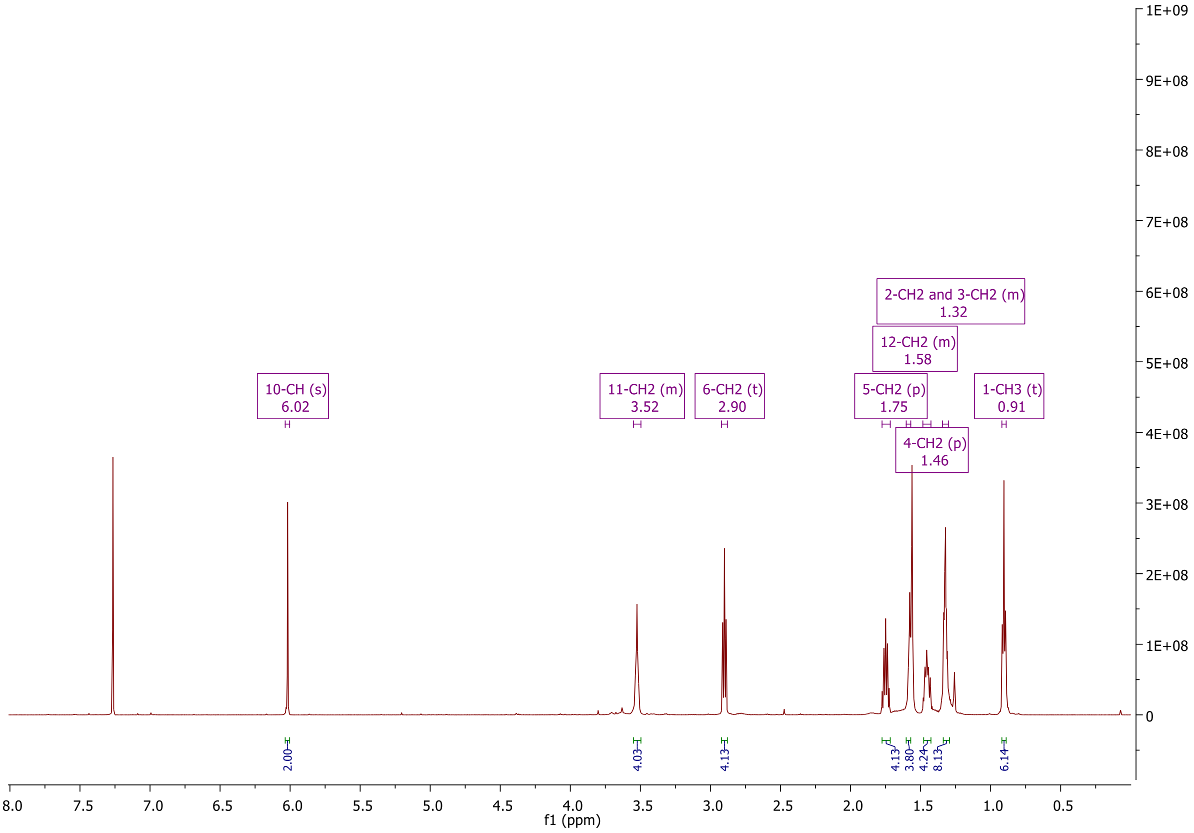

2-CH2 and 3-CH2 (m)
1.32
12-CH2 (m)
1.58
10-CH (s)
6.02
11-CH2 (m)
3.52
6-CH2 (t)
2.90
5-CH2 (p)
1.75
1-CH3 (t)
0.91
4-CH2 (p)
1.46
2.00
4.03
4.13
4.13
3.80
4.24
8.13
6.14
f1 (ppm)

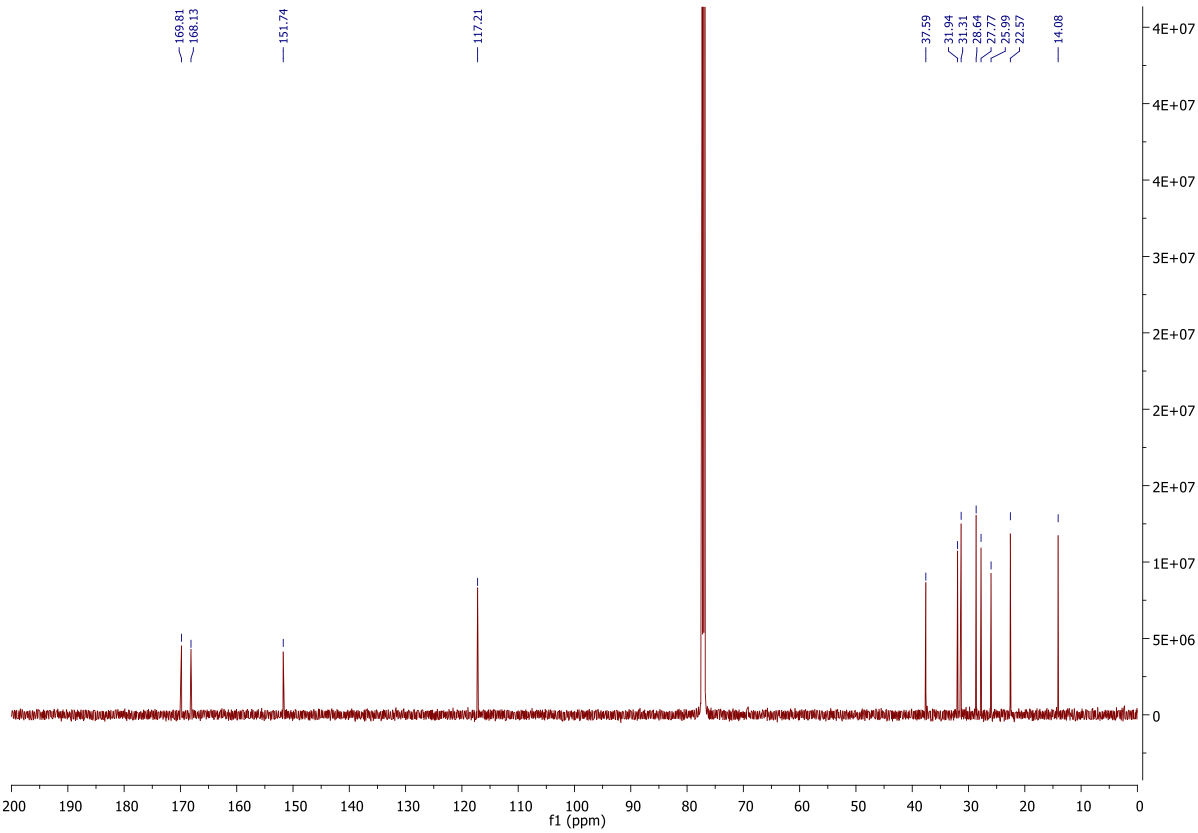

169.81
168.13
151.74
117.21
37.59
31.94
31.31
28.64
27.77
25.99
22.57
14.08
f1 (ppm)

(3a*R*,3b*S*,6a*R*,6b*S*)-3a,3b-*Bis*(hexylthio)tetrahydro-2,5-butanocyclobuta[1,2-*c*:3,4-*c'*]dipyrrole-1,3,4,6-tetraone, 5b

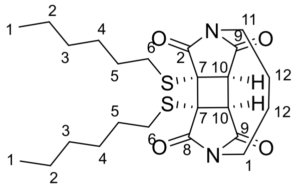

A 2.5 mM solution of **4b** (36.1 mg, 0.0750 mmol) in 1:5 DCM:MeCN (30 mL) was degassed for 30 min. The solution was then irradiated in pyrex glassware for 15 min whilst stirring at room temperature. The solvent was removed *in vacuo* to afford the title compound as a yellow solid (36.1 mg, 0.0750 mmol) in a quantitative yield.

m.p. 116-120 °C; ^{1}H NMR (700 MHz, $CDCl_3$): δ 3.67 – 3.58 (m, 6H, 10-C*H* and 11-C*H₂*), 2.90 (dt, *J* = 10.9, 7.4 Hz, 2H, 6-C*H*H), 2.78 (dt, *J* = 10.9, 7.4 Hz, 2H, 6-CH*H*), 1.94 – 1.75 (m, 4H, 12-CH_2), 1.60 (p, *J* = 7.6 Hz, 4H, 5-CH_2), 1.39 (p, *J* = 7.5 Hz, 4H, 4-CH_2), 1.31 – 1.27 (m, 8H, 2-CH_2 and 3-CH_2), 0.88 (t, *J* = 6.9 Hz, 6H, 1-CH_3); ^{13}C NMR (151 MHz, $CDCl_3$): δ 172.7 (*C*-8/9), 171.9 (*C*-8/9), 57.9 (*C*-7), 45.6 (*C*-10), 39.8 (*C*-11), 31.4 (C-3), 30.7 (*C*-6), 29.2 (*C*-5), 28.6 (*C*-4), 25.0 (*C*-12), 22.6 (*C*-2), 14.1 (*C*-1); IR (solid, cm^{-1}): 2920 (m), 1706 (s); LRMS (ASAP+) (m/z, %): 481.2 ([M+H]$^+$, 100); HRMS: exact mass calculated for [M+H]$^+$, $[C_{24}H_{37}N_2O_4S_2]^+$ 481.2195, found 481.2198.

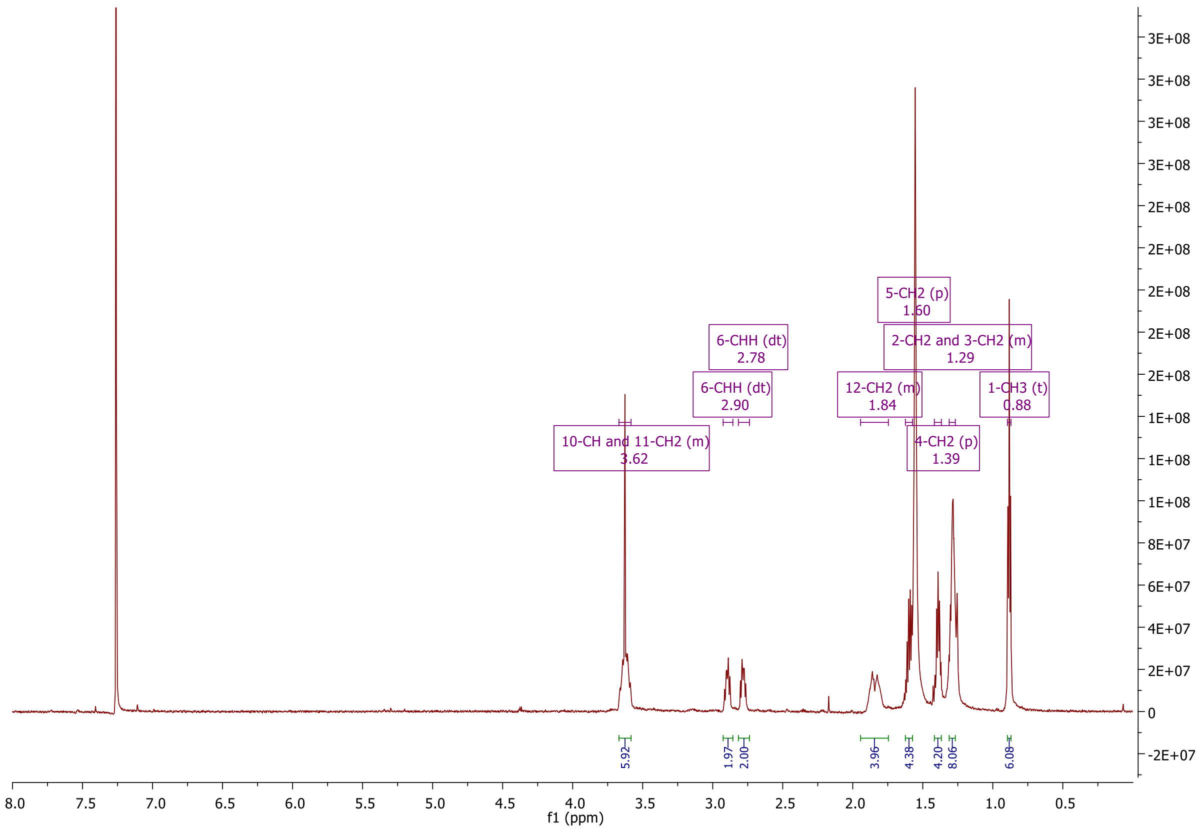
5-CH2 (p)
1.60
6-CHH (dt)
2.78
2-CH2 and 3-CH2 (m)
1.29
6-CHH (dt)
2.90
12-CH2 (m)
1.84
1-CH3 (t)
0.88
10-CH and 11-CH2 (m)
3.62
4-CH2 (p)
1.39
5.92
1.97
2.00
3.96
4.38
4.20
8.06
6.08
f1 (ppm)

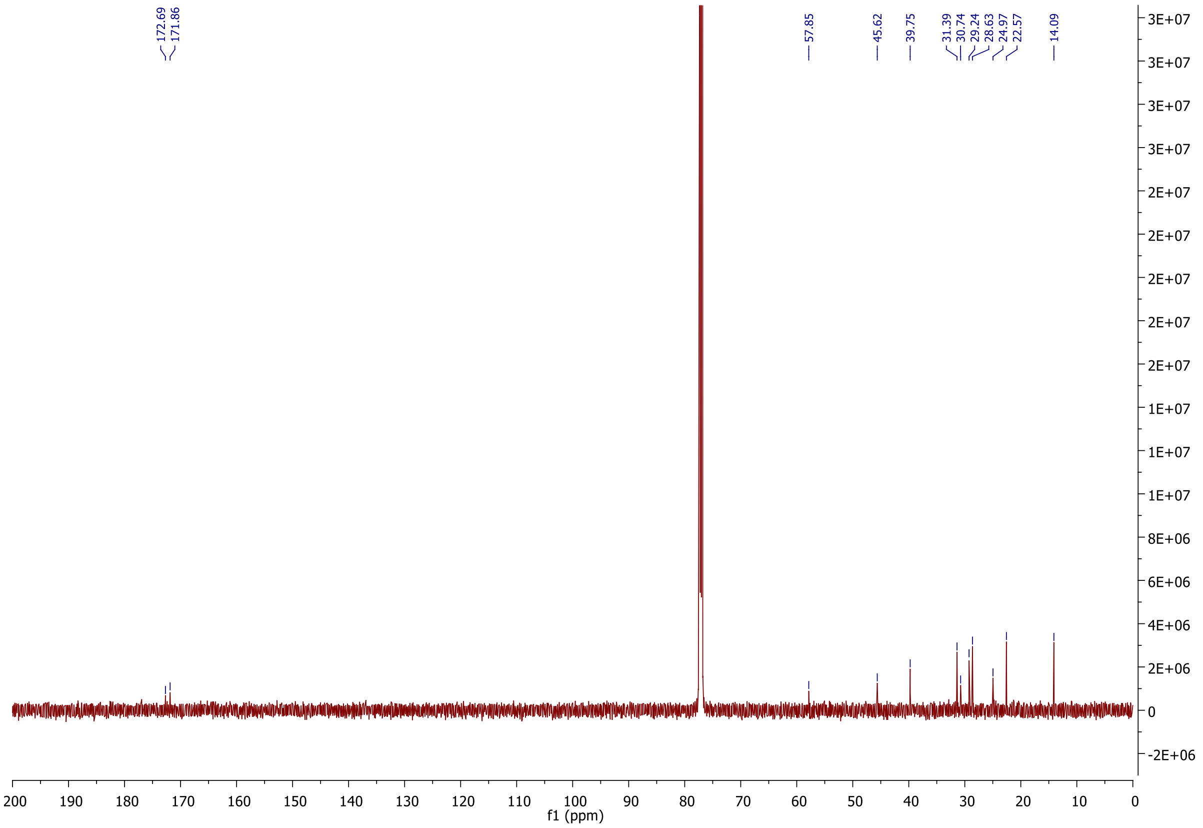
172.69
171.86
57.85
45.62
39.75
31.39
30.74
29.24
28.63
24.97
22.57
14.09
f1 (ppm)

1,1'-(2,2-Dimethylpropane-1,3-diyl)*bis*(3-bromo-1*H*-pyrrole-2,5-dione), 3c

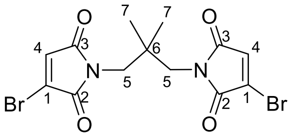

To a solution of 3-bromomaleic anhydride (0.557 mL, 6.00 mmol) in acetic acid (10 mL), 2,2-dimethyl,1,3-diaminopropane (0.180 mL, 1.50 mmol) was added and the reaction mixture was heated to reflux for 3 h. The reaction mixture was cooled and the solvent was then removed *in vacuo*, azeotroping with toluene (3 x 10 mL). The product was separated between EtOAc (20 mL) and saturated $NaHCO_3$ solution (20 mL). The organic layer was further washed with brine (20 mL), dried over $MgSO_4$, and the solvent removed *in vacuo*. The crude product was purified by silica flash chromatography (gradient elution of 0-40% EtOAc in cyclohexane) to afford the title compound as an off-white solid (74.2 mg, 0.177 mmol) in a 12% yield.

m.p. 162-166 °C; ^{1}H NMR (600 MHz, $CDCl_3$): δ 6.91 (s, 2H, 4-C*H*), 3.45 (s, 4H, 5-C*H₂*), 0.90 (s, 6H, 7-C*H₃*); ^{13}C NMR (151 MHz, $CDCl_3$): δ 169.2 (*C*-2/3), 166.1 (*C*-2/3), 132.1 (*C*-4), 131.6 (*C*-1), 47.9 (*C*-5), 39.0 (*C*-6), 24.1 (*C*-7); IR (solid, cm^{-1}): 3104 (w), 2942 (w), 1699 (s), 1589 (m); LRMS (NS+) (m/z, %): 418.9 ([^{79}Br^{79}BrM+H]$^+$, 2), 420.9 ([^{79}Br^{81}BrM+H]$^+$, 4), 422.9 ([^{81}Br^{81}BrM+H]$^+$, 2), 450.9 ([^{79}Br^{79}Br+CH_3OH+H]$^+$, 53), 452.9 ([^{79}Br^{81}BrM+ CH_3OH+H]$^+$, 100), 454.9 ([^{81}Br^{81}BrM+ CH_3OH+H]$^+$, 53); HRMS: exact mass calculated for [^{79}Br^{79}BrM+CH_3OH+H]$^+$, [$C_{14}H_{17}{}^{79}Br_2N_2O_5$]$^+$ 450.9499, found 450.9497.

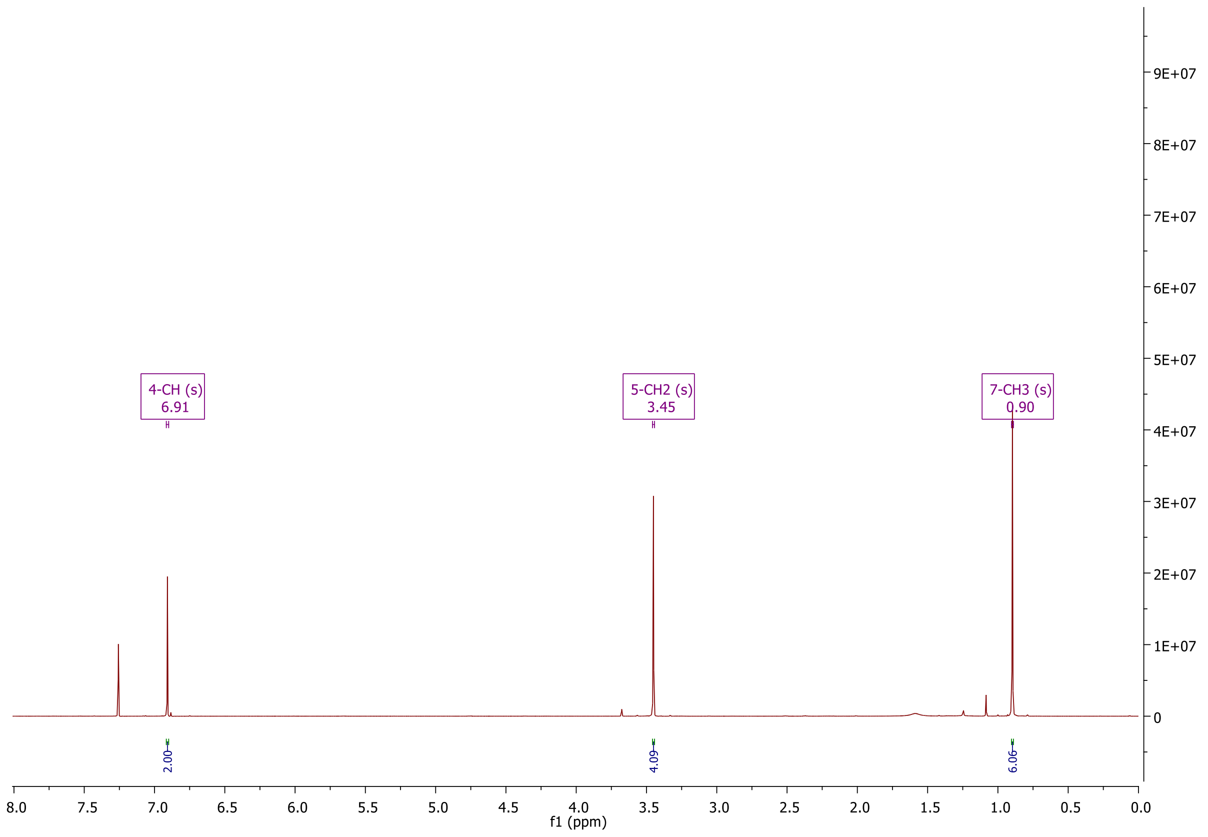
4-CH (s)
6.91
5-CH2 (s)
3.45
7-CH3 (s)
0.90
2.00
4.09
6.06
f1 (ppm)

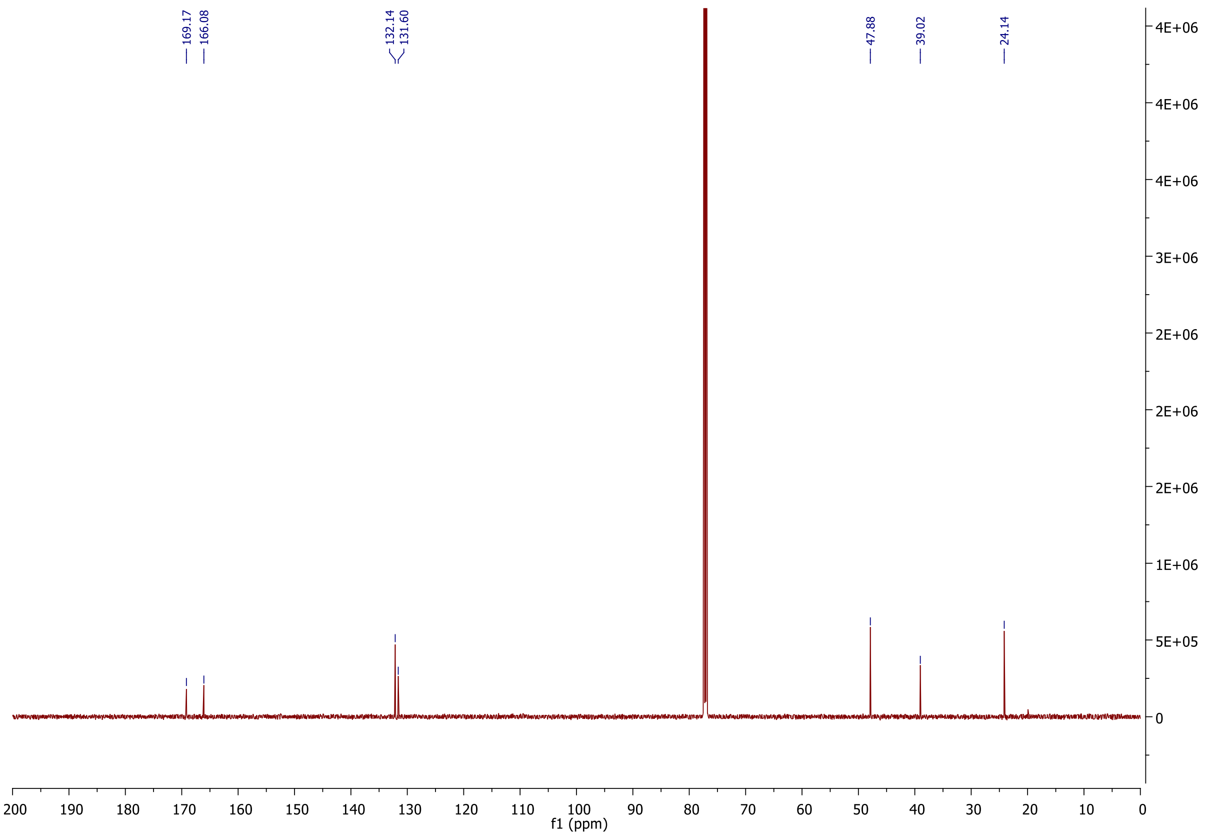
169.17
166.08
132.14
131.60
47.88
39.02
24.14
f1 (ppm)

1,1'-(2,2-Dimethylpropane-1,3-diyl)*bis*(3-(hexylthio)-1*H*-pyrrole-2,5-dione), 4c

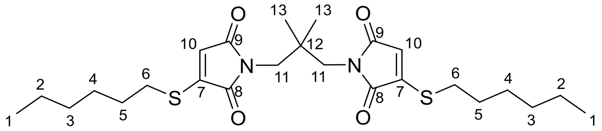

To a solution of **3c** (61.8 mg, 0.150 mmol) in DCM (30 mL), triethylamine (46.0 μL, 0.330 mmol) was added, followed by hexanethiol (46.6 μL, 0.330 mmol). The reaction mixture was stirred for 30 min at room temperature. The solvent was removed *in vacuo*. The crude product was then purified by silica flash chromatography (gradient elution of 0-40% EtOAc in cyclohexane) to afford the title compound as a yellow wax (61.8 mg, 0.150 mmol) in a quantitative yield.

^{1}H NMR (600 MHz, $CDCl_3$): δ 6.04 (s, 2H, 10-C*H*), 3.37 (s, 4H, 11-C*H₂*), 2.88 (t, *J* = 7.3 Hz, 4H, 6-CH_2), 1.73 (p, *J* = 7.5 Hz, 4H, 5-CH_2), 1.43 (p, *J* = 7.6 Hz, 4H, 4-CH_2), 1.32 – 1.27 (m, 8H, 2-CH_2 and 3-CH_2), 0.89 – 0.84 (m, 12H, 1-CH_3 and 13-CH_3); ^{13}C NMR (151 MHz, $CDCl_3$): δ 170.2 (*C*-8/9), 168.7 (*C*-8/9), 151.8 (*C*-7), 117.4 (*C*-10), 47.3 (*C*-11), 39.0 (*C*-12), 32.0 (*C*-6), 31.3 (*C*-3), 28.7 (*C*-4), 27.8 (*C*-5), 24.2 (*C*-13), 22.6 (*C*-2), 14.1 (*C*-1); IR (wax, cm^{-1}): 2928 (m), 1702 (s), 1557 (m); LRMS (ES+) (m/z, %): 495.2 ([M+H]$^+$, 100); HRMS: exact mass calculated for [M+H]$^+$, $[C_{25}H_{39}N_2O_4S_2]^+$ 495.2346, found 495.2346; UV (MeCN): ε_{354} = 5100 cm^{-1} M^{-1}.

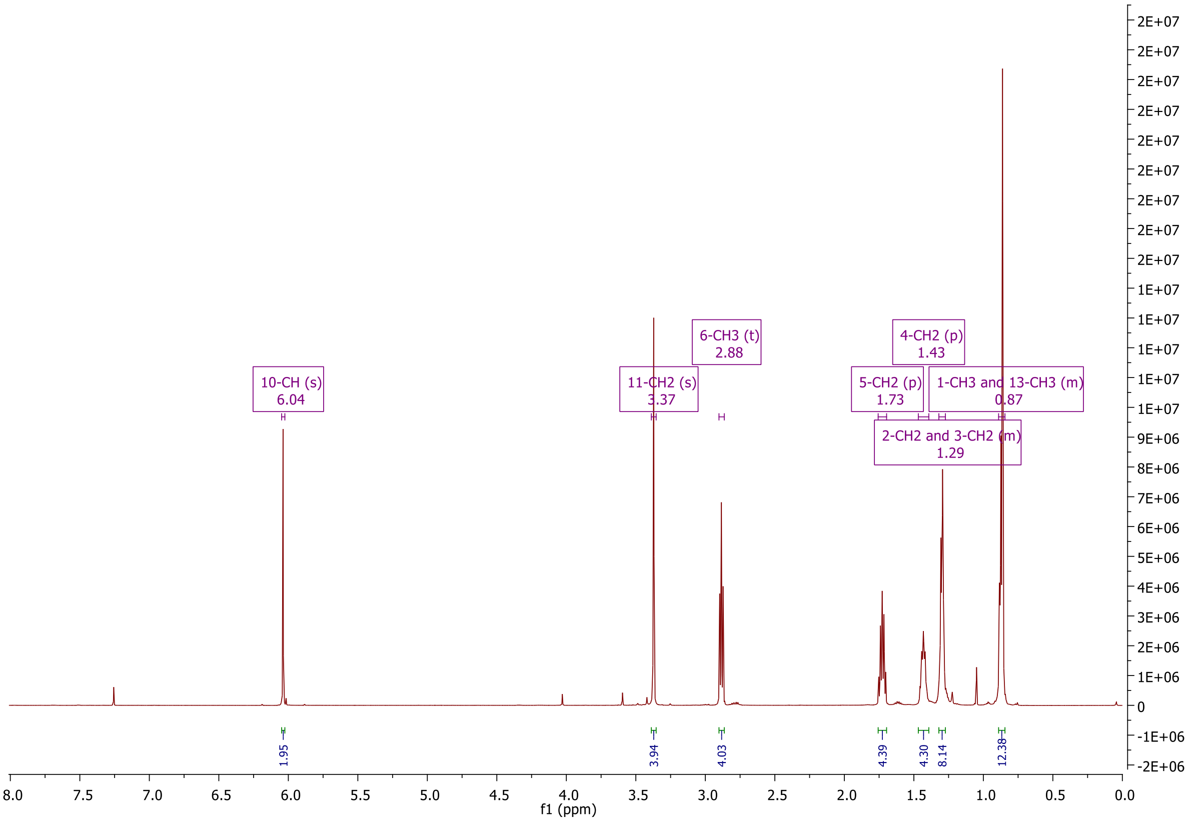
10-CH (s)
6.04
11-CH2 (s)
3.37
6-CH3 (t)
2.88
5-CH2 (p)
1.73
4-CH2 (p)
1.43
1-CH3 and 13-CH3 (m)
0.87
2-CH2 and 3-CH2 (m)
1.29
1.95
3.94
4.03
4.39
4.30
8.14
12.38
f1 (ppm)

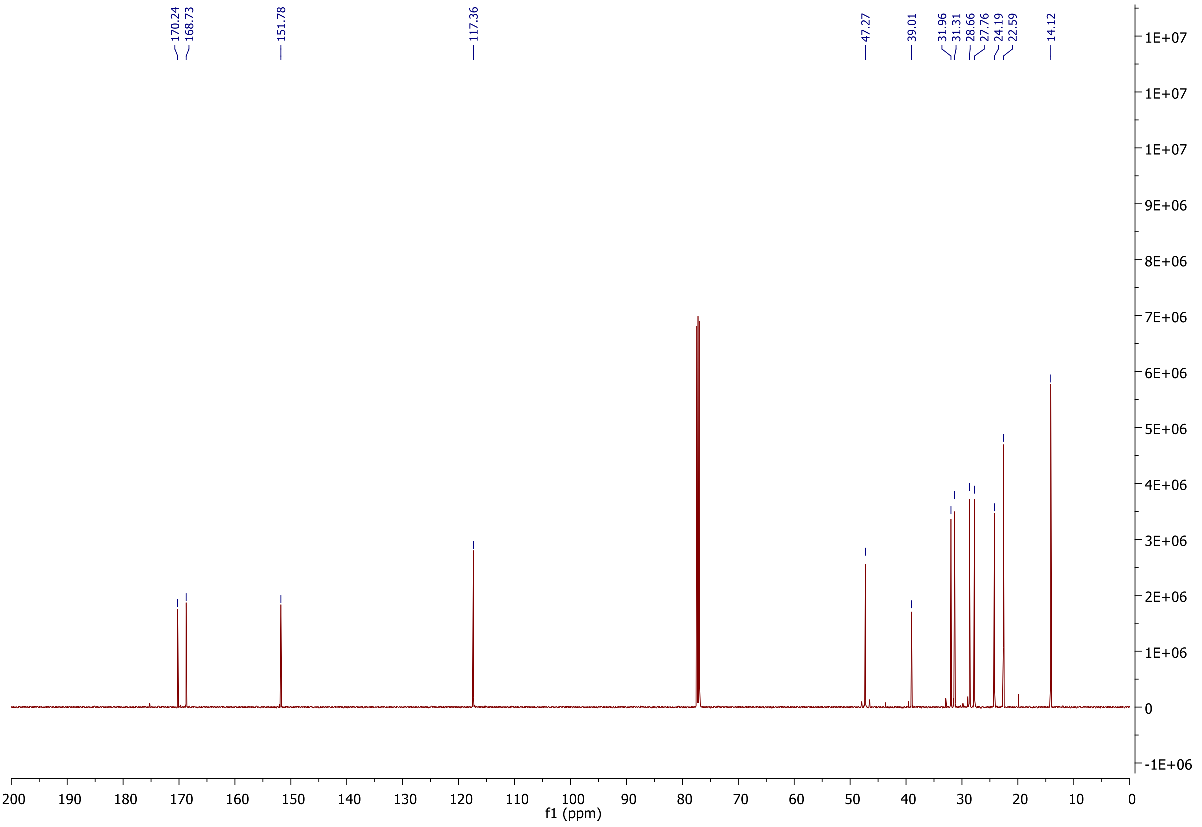
170.24
168.73
151.78
117.36
47.27
39.01
31.96
31.31
28.66
27.76
24.19
22.59
14.12
f1 (ppm)

(3a*R*,3b*S*,6a*R*,6b*S*)-3a,3b-*Bis*(hexylthio)-8,8-dimethyltetrahydro-2,5-propanocyclobuta[1,2-c:3,4-c']dipyrrole-1,3,4,6-tetraone, 5c

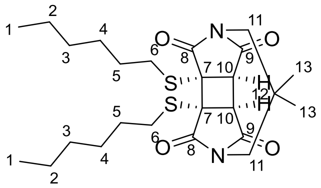

A 2.5 mM solution of **4c** (37.1 mg, 0.0750 mmol) in MeCN (30 mL) was degassed with argon for 30 min. The solution was then irradiated in pyrex glassware for 10 min whilst stirring at room temperature. The solvent was removed *in vacuo* to afford the title compound as a yellow solid (37.1 mg, 0.0750 mmol) in a quantitative yield.

m.p. 162-164 °C; ^{1}H NMR (700 MHz, $CDCl_3$): δ 3.89 (d, *J* = 14.8 Hz, 2H, 11-C*H*H), 3.82 (d, *J* = 14.8 Hz, 2H, 11-C*H*H), 3.65 (s, 2H, 10-C*H*), 2.91 (ddd, *J* = 11.5, 8.2, 6.7 Hz, 2H, 6-C*H*H), 2.76 (ddd, *J* = 11.5, 8.3, 6.7 Hz, 2H, 6-CH*H*), 1.61 – 1.54 (m, 6H, 5-CH_2 (superimposed with water peak)), 1.40 – 1.36 (m, 4H, 4-CH_2), 1.34 (s, 3H, 13-CH_3), 1.29 (s, 3H, 13-CH_3), 1.29 – 1.24 (m, 8H, 2-CH_2 and 3-CH_2), 0.88 (t, *J* = 7.1 Hz, 6H, 1-CH_3); ^{13}C NMR (176 MHz, $CDCl_3$): δ 172.6 (*C*-8/9), 171.8 (*C*-8/9), 58.0 (*C*-7), 51.0 (*C*-11), 45.1 (*C*-10), 41.3 (*C*-12), 32.4 (*C*-13), 32.1 (*C*-13), 31.4 (*C*-3), 30.8 (*C*-6), 29.4 (*C*-5), 28.6 (*C*-4), 22.6 (*C*-2), 14.1 (*C*-1); IR (solid, cm^{-1}): 2925 (m), 1713 (s); LRMS (ES+) (m/z, %): 495.2 ($[M+H]^+$, 67), 512.3 ($[M+NH_4]^+$, 100); HRMS: exact mass calculated for $[M+H]^+$, $[C_{25}H_{39}N_2O_4S_2]^+$ 495.2346, found 495.2343.

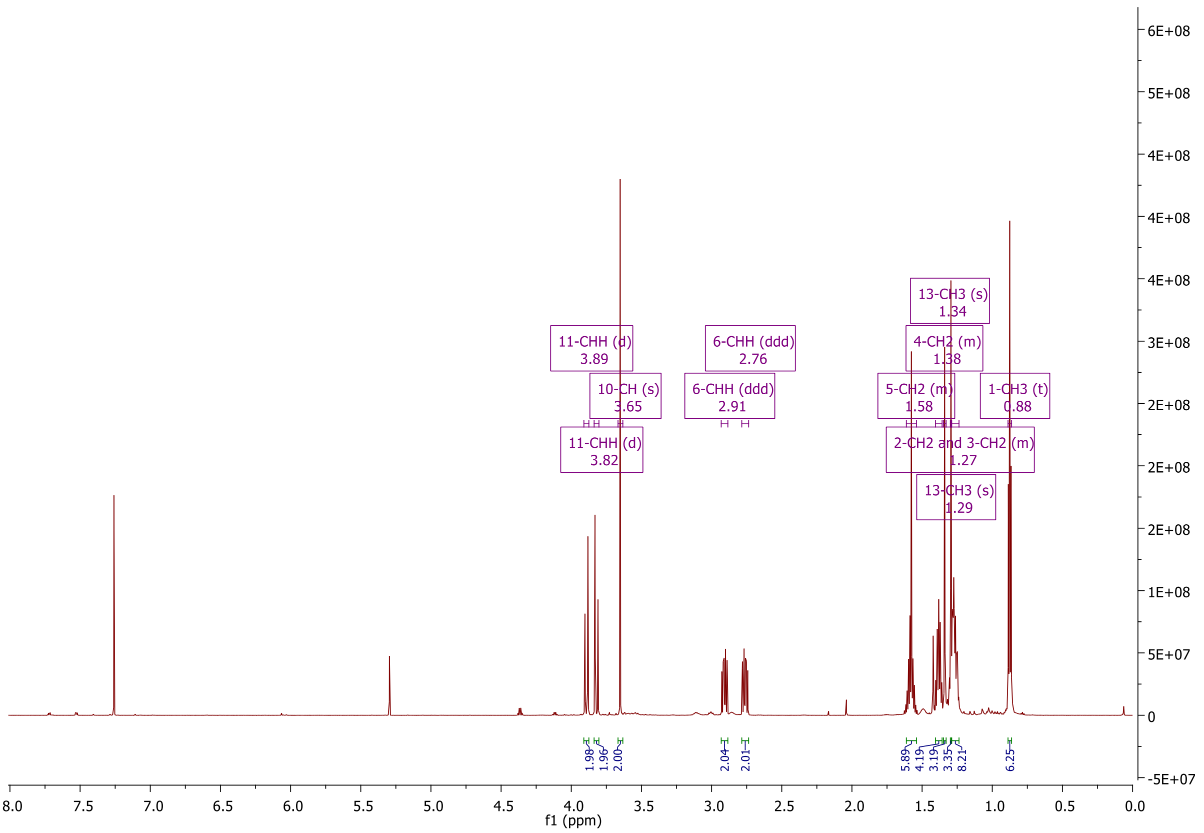
11-CHH (d)
3.89
10-CH (s)
3.65
11-CHH (d)
3.82
6-CHH (ddd)
2.76
6-CHH (ddd)
2.91
13-CH3 (s)
1.34
4-CH2 (m)
1.38
5-CH2 (m)
1.58
1-CH3 (t)
0.88
2-CH2 and 3-CH2 (m)
1.27
13-CH3 (s)
1.29
1.98
1.96
2.00
2.04
2.01
5.89
4.19
3.19
3.35
8.21
6.25
f1 (ppm)

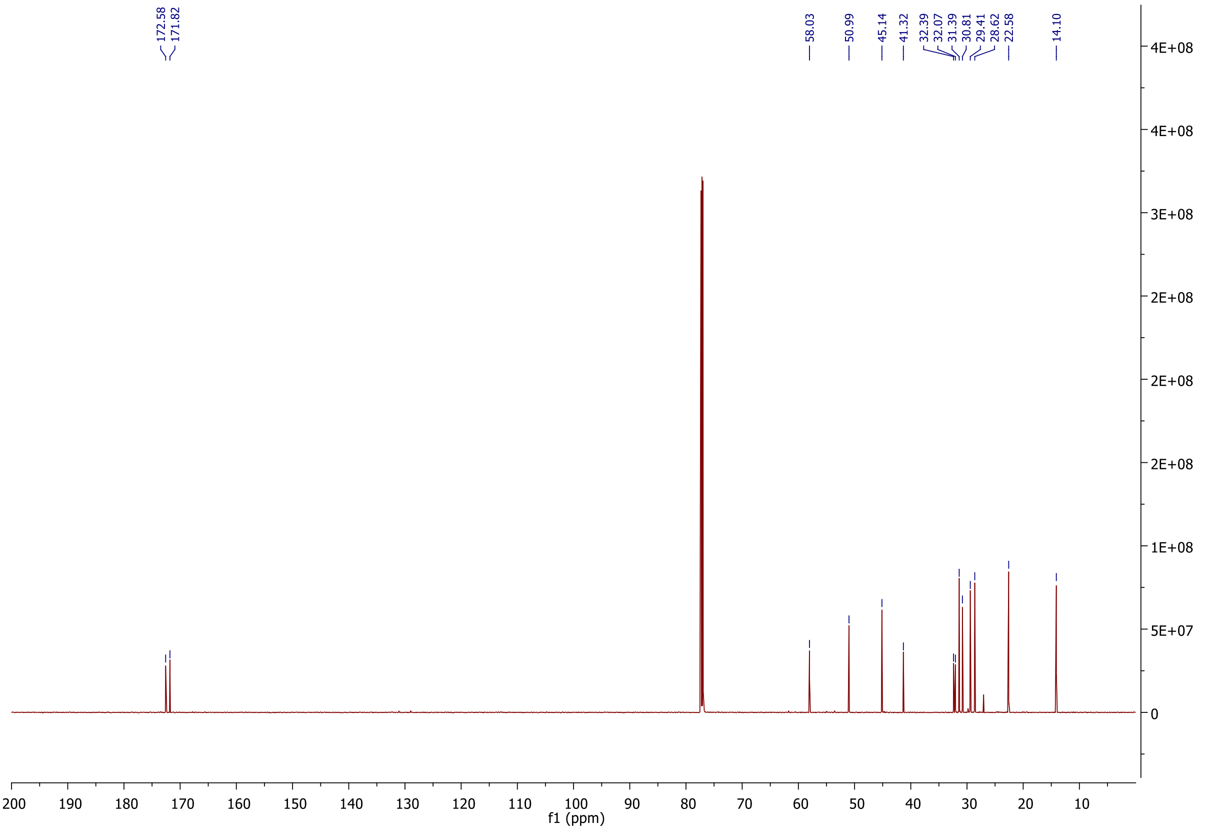
172.58
171.82
58.03
50.99
45.14
41.32
32.39
32.07
31.39
30.81
29.41
28.62
22.58
14.10
f1 (ppm)

1-Butyl-1*H*-pyrrole-2,5-dione, 9[4]

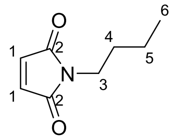

To a solution of maleic anhydride (735 mg, 7.50 mmol) in acetic acid (5 mL), butylamine (0.494 mL, 5.00 mmol) was added and the reaction mixture was heated to reflux for 3 h. The reaction mixture was cooled and the solvent was then removed *in vacuo*, azeotroping with toluene (3 x 10 mL). The product was dissolved in EtOAc (20 mL) and washed with $NaHCO_3$ (20 mL). The product was further washed with brine (20 mL), dried over $MgSO_4$, and the solvent removed *in vacuo*. The crude product was purified by silica flash chromatography (gradient elution of 0-40% EtOAc in cyclohexane) to afford the title compound as a yellow oil (311 mg, 2.03 mmol) in a 41% yield.

^{1}H NMR (700 MHz, $CDCl_3$): δ 6.67 (s, 2H, 1-C*H*), 3.51 (t, *J* = 7.3 Hz, 2H, 3-CH_2), 1.56 (p, *J* = 7.5 Hz, 2H, 4-CH_2), 1.30 (h, *J* = 7.4 Hz, 2H, 5-CH_2), 0.92 (t, *J* = 7.4 Hz, 3H, 6-CH_3); ^{13}C NMR (176 MHz, $CDCl_3$) δ 171.0 (*C*-2), 134.2 (*C*-1), 37.8 (*C*-3), 30.7 (*C*-4), 20.1 (*C*-5), 13.7 (*C*-6); IR (oil, cm^{-1}): 2961 (w), 1701 (s), 1407 (m); LRMS (EI+) (m/z, %): 109.9 ($[M-CH_2CH_2CH_3]^+$, 100), 153.0 ($[M]^+$, 30); UV (DCM): ε_{292} = 920 cm^{-1} M^{-1}; UV (MeCN): ε_{297} = 690 cm^{-1} M^{-1}.

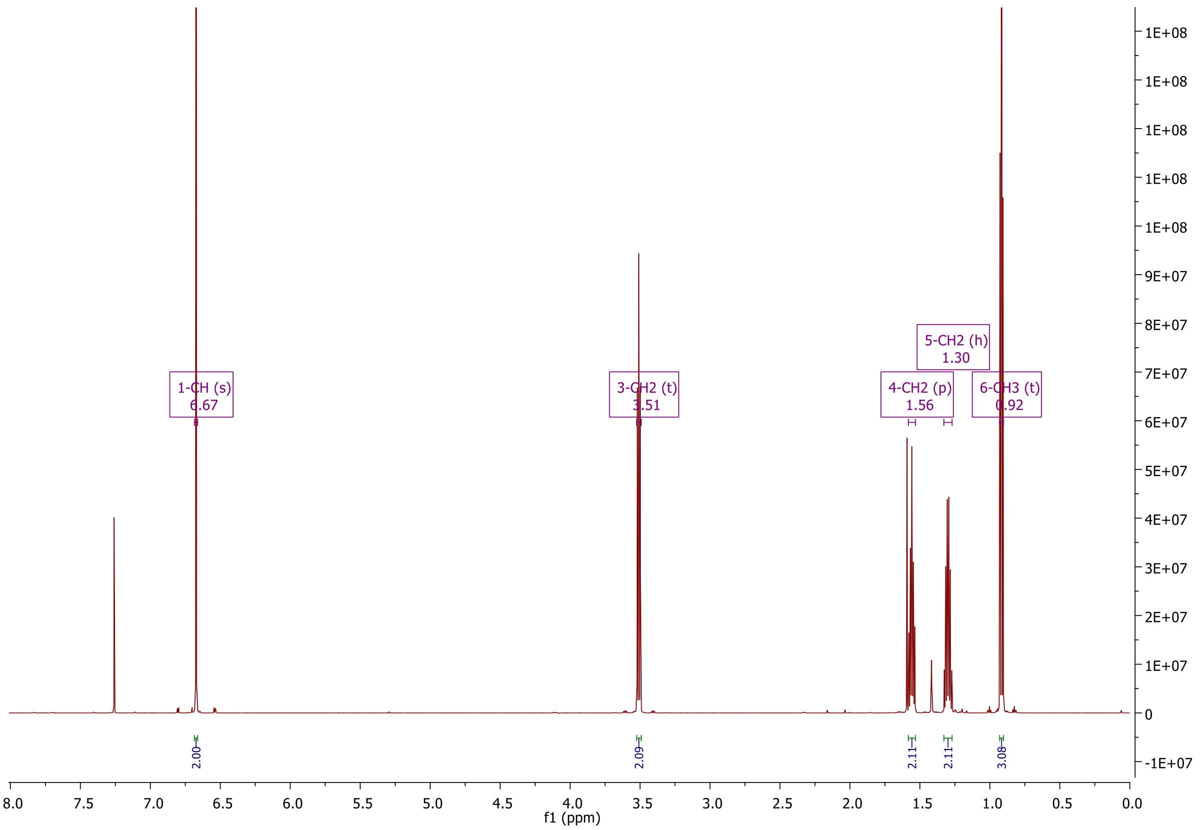
1-CH (s)
6.67
3-CH2 (t)
3.51
5-CH2 (h)
1.30
4-CH2 (p)
1.56
6-CH3 (t)
0.92
2.00
2.09
2.11
2.11
3.08
f1 (ppm)

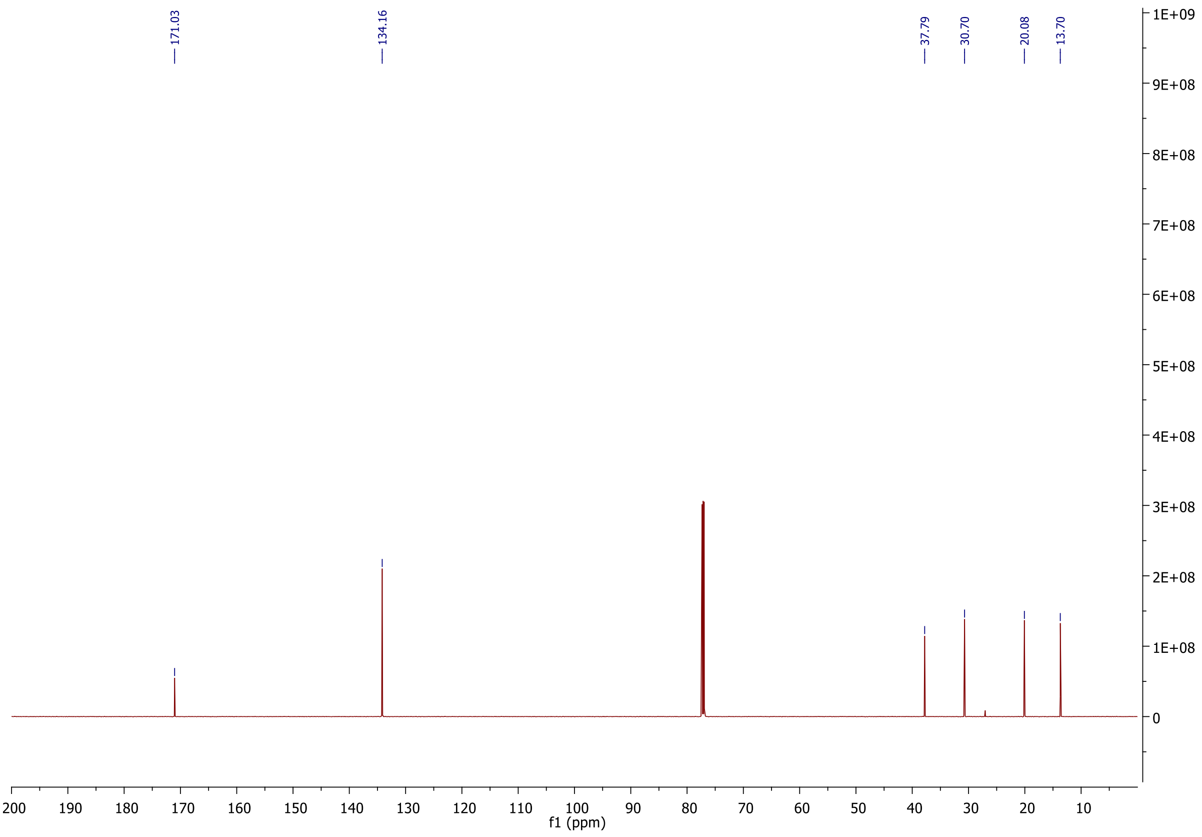
171.03
134.16
37.79
30.70
20.08
13.70
f1 (ppm)

1,1'-(Propane-1,3-diyl)*bis*(1*H*-pyrrole-2,5-dione), 10[5]

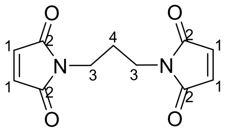

To a solution of maleic anhydride (981 mg, 10.0 mmol) in acetic acid (10 mL), 1,3-diaminopropane (0.210 mL, 2.50 mmol) was added and the reaction mixture was heated to reflux for 3 h. The reaction mixture was cooled and the solvent was then removed *in vacuo*, azeotroping with toluene (3 x 10 mL). The product was separated between EtOAc (20 mL) and saturated $NaHCO_3$ solution (20 mL). The organic layer was further washed with brine (20 mL), dried over $MgSO_4$, and the solvent removed *in vacuo*. The crude product was purified by silica flash chromatography (gradient elution of 0-60% EtOAc in cyclohexane) to afford the title compound as a white solid (253 mg, 1.08 mmol) in a 43% yield.

m.p. 168-170 °C (lit. 174 °C)[5]; ^{1}H NMR (700 MHz, $CDCl_3$): δ 6.70 (s, 4H, 1-C*H*), 3.54 (t, *J* = 7.2 Hz, 4H, 3-C*H*$_2$), 1.94 (p, *J* = 7.2 Hz, 2H, 4-C*H*$_2$); ^{13}C NMR (176 MHz, $CDCl_3$): δ 170.7 (*C*-2), 134.3 (*C*-1), 35.5 (*C*-3), 27.5 (*C*-4); IR (solid, cm^{-1}): 3087 (m), 2921 (m), 1687 (s), 1583 (m); LRMS (ES+) (m/z, %): 235.1 ([M]$^+$, 92), 257.1 ([M+Na]$^+$, 100); UV (MeCN): ε_{295} = 1200 cm^{-1} M^{-1}.

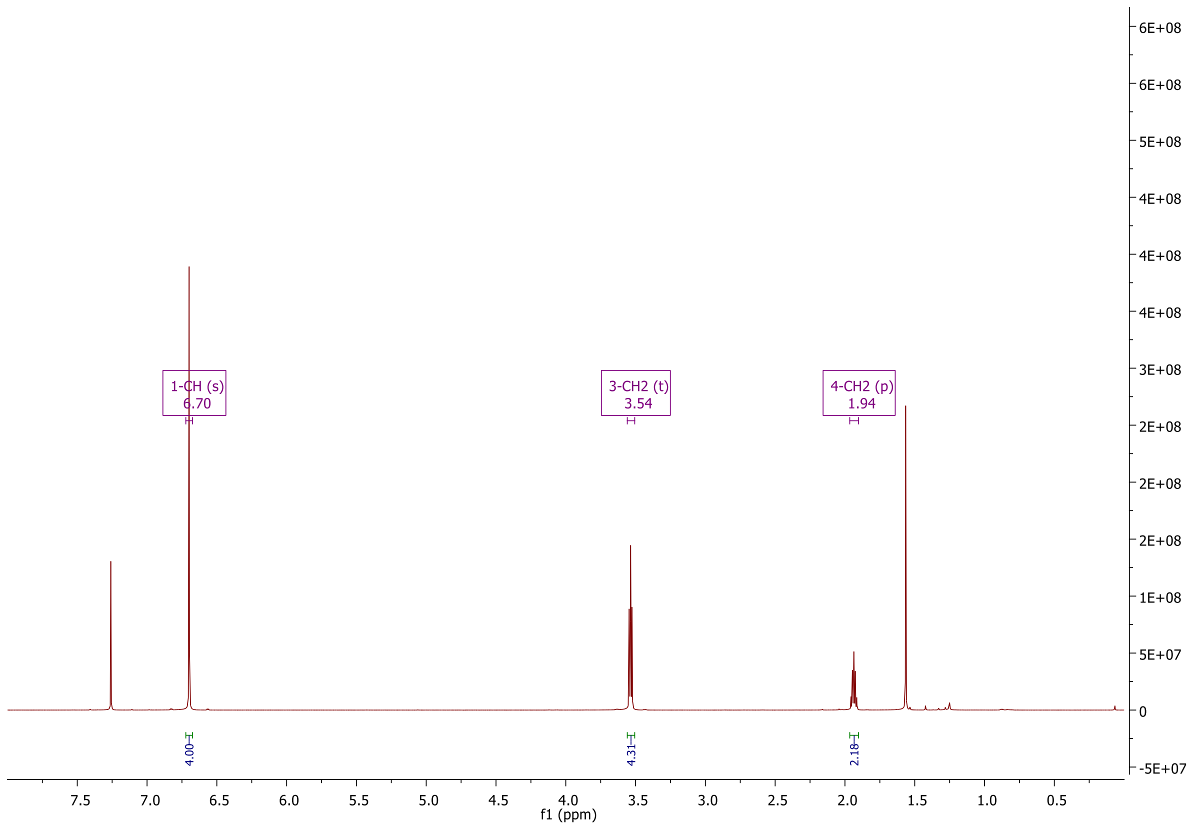
1-CH (s)
6.70
3-CH2 (t)
3.54
4-CH2 (p)
1.94
4.00
4.31
2.18
f1 (ppm)

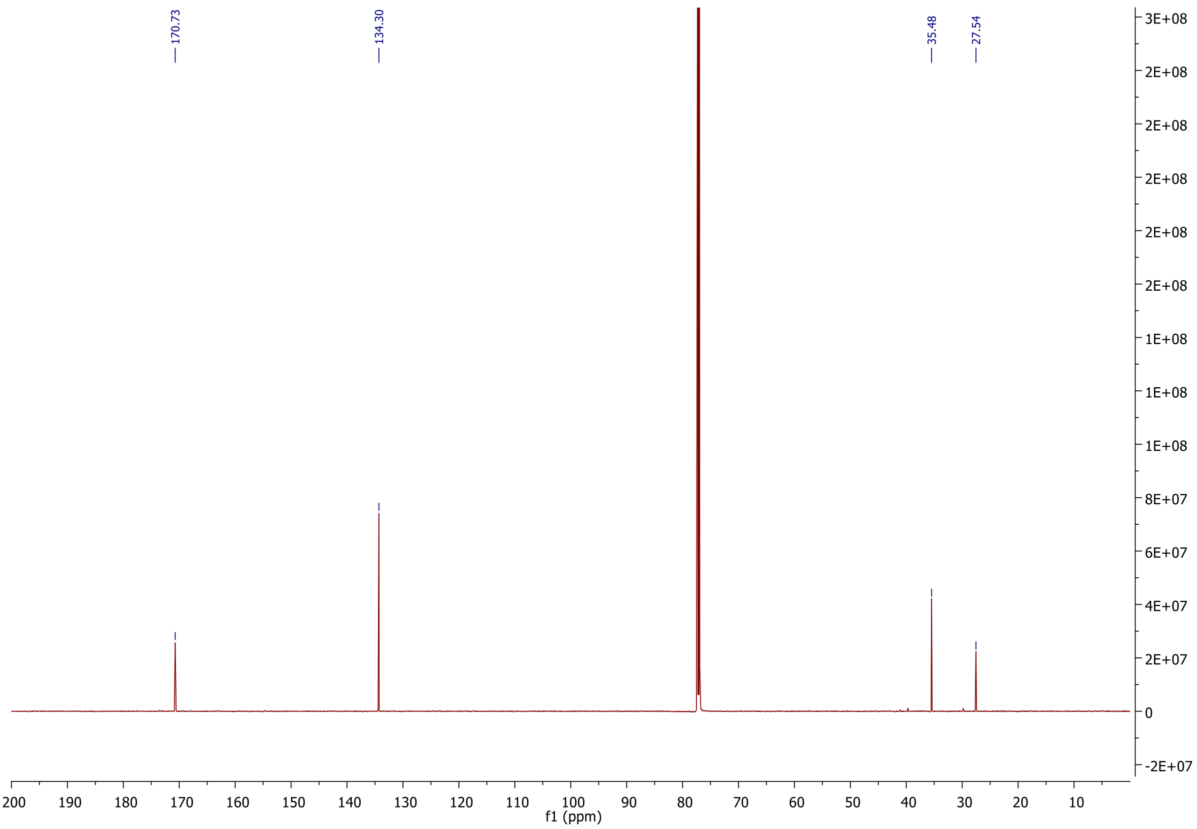
170.73
134.30
35.48
27.54
f1 (ppm)

2. Bioconjugation

2.1. General remarks

All chemical reagents and solvents were purchased from chemical suppliers and used as received, without any further purification. Trastuzumab (Herceptin) was purchased from UCLH Pharmacy in its clinical formulation (lyophilised). This was solubilised in LCMS grade H_2O to a concentration of 68.9 μM (10.0 mg/mL). Aliquots of 0.50 mL trastuzumab were stored at -20 ˚C for up to 6 months. Stock solutions of reagents were made in double-deionised H_2O (dH_2O), MeCN or DMF as specified, unless otherwise stated. Conjugation experiments were conducted in 1.5 mL Eppendorf tubes at atmospheric pressure at the stated temperature. Eppendorf 5415 R and VWR Galaxy 14D microcentrifuges were utilised for centrifugation and temperature was controlled by carrying out the reactions using an Eppendorf thermomixer comfort heating block. For the irradiation of Fab conjugates, the samples were placed in a plastic micro UV- cuvette. A 365 nm UVG2 Labino LED torch held at a distance of 14 cm from the sample was used for the irradiation. Ultrafiltration was carried out with Sartorius Vivaspin 500 centrifugal concentrators with a molecular weight cut-off (MWCO) of 10 kDa. UV/Vis absorbance was measured using a ThermoFisher NanoDrop One/One Microvolume UV-Vis Spectrophotometer. These readings were used to determine the protein concentration using the molecular extinction coefficients of ε_{280} = 215,380 cm^{-1} M^{-1} for full antibody and ε_{280} = 68,590 cm^{-1} M^{-1} for Fab.[6] The following buffers were made using dH_2O; the pH adjusted using 1 M HCl or 1 M NaOH, using a Jenway 3510 pH meter; and filter-sterilised before use:

NaOAc buffer pH 3.1: 20 mM NaOAc

Digest buffer pH 6.8: 50 mM phosphate, 150 mM, NaCl, 1mM EDTA (10 mM DTT as specified)

BBS pH 7.4: 25 mM boric acid, 25 mM NaCl, 1 mM EDTA

PBS pH 6.0: 22 mM NaH_2PO_4, 3 mM Na_2HPO_4, 25 mM NaCl, 1 mM EDTA

<u>SDS-PAGE analysis</u>

Conjugation reactions were monitored by non-reducing 12% glycine SDS-PAGE with a 6% stacking gel, which were prepared in-house using the components listed in **Table 1**. The gels can be stored at 4 °C for up to a month. Gel samples were made with a 1:1 ratio of 3 μg of Fab sample and 2 x SDS non-reducing loading dye (composition of 5 x loading dye: 1.6 mL of 10 % (w/v) SDS, 8 mL of glycerol, 4 mL of dH_2O, 1 mL of 0.5 M Tris buffer pH 6.8, 25 mg of bromophenol blue). The gel samples were heated at 75 °C for 5 min before loading to the gel (6 μL per lane). The ThermoScientific Page Ruler Plus Pre-Stained Protein Ladder was used as a mass reference (4 μL). The gel was run at a constant voltage of 200 V for 60 min in 1 x SDS running buffer (composition for 10 x SDS running buffer: 30 g of Tris base, 144 g of glycine, 10 g of SDS in 1 L of dH_2O, pH 8.3). Gels were stained in Coomassie blue stain (10%

ammonium sulfate (100 g), 0.1% Coomassie G-250 (500 mg), 3% phosphoric acid (30 mL), ethanol (200 mL), and water (1 L). Gels were destained with water.

Components	12 % Separating	6 % Stacking
dH_2O	7.95 mL	5.3 mL
Acrylamide	9.6 mL	2.0 mL
1.5 M Tris pH 8.8	6.0 mL	-
0.5 M Tris pH 6.8	-	2.5 mL
10 % SDS	240 μL	100 μL
10 % APS	240 μL	100 μL
TEMED	24 μL	10 μL

Table 1: *Components of 12% glycine SDS-PAGE required for four gels.*

LCMS preparation and analysis

The trastuzumab Fab samples were buffer exchanged into LCMS grade H_2O using ThermoFisher Zeba Spin desalting columns as per manufacturer's instruction, followed by adjusting the concentration to 5 μM with LCMS grade H_2O.

LCMS of trastuzumab Fab conjugates was performed with an Agilent 6510 QTOF coupled to an Agilent 1200 HPLC. An Agilent PLRPS 1000 Å column (150 mm x 2.1 mm, 8 μM particle size) at 60 °C, 10 μL injection volume and 300 μL/min flow rate was used. Solvent A was LCMS grade H_2O (0.1% formic acid), solvent B was LCMS grade MeCN (0.1% formic acid). The elution gradient in **Table 2** was used.

Time (min)	Solvent A (%)	Solvent B (%)
0.00	85	15
2.00	85	15
3.00	68	32
4.00	68	32
14.00	65	35
18.00	5	95
20.00	5	95
22.00	85	15
25.00	85	15

Table 2: *LCMS gradient for trastuzumab Fab conjugates.*

Agilent 6510 QTOF was operated in a positive polarity mode, coupled with an ESI ion source, set up with the following parameters: VCap of 3500 V, gas temperature of 350 °C, dry gas flow rate at 10 L/min and nebuliser of 30 psig. MS Tof was acquired under the following conditions: fragmentor at 350 V, skimmer at 65 V and acquisition rate of 0.5 spectra/s in a profile mode, within a scan range 700-5000 m/z. The .d data was analysed by deconvolution of the spectra to zero-charge mass spectra using a maximum entropy deconvolution algorithm within the MassHunter software version B.07.00.

Preparation of trastuzumab Fab fragment

A literature protocol involving sequential enzymatic digestion was followed.[7] Trastuzumab (0.50 mL, 68.9 μM, 10.0 mg/mL) was buffer exchanged into NaOAc buffer *via* ultrafiltration (10 kDa MWCO). The concentration was determined and adjusted to 44.0 μM with NaOAc buffer. Immobilised pepsin (0.15 mL) was also washed with NaOAc buffer (3 × 0.40 mL) using a Pierce centrifuge column. Trastuzumab (0.50 mL, 44.0 μM) was then added to the pepsin and the mixture was incubated at 37 °C for 5 h, under constant agitation (1,100 rpm). The pepsin resin was then removed from the digest by washing with digest buffer (4 × 0.40 mL). The digest was combined with the washes and a 5 mL falcon tube Vivaspin was used to concentrate to a volume of ~0.50 mL. The concentration of the F(ab')$_2$ was determined and adjusted to 150.0 μM with digest buffer. Next, immobilised papain (0.65 mL) was activated with digest buffer containing 10 mM DTT, by incubation at 37 °C for 90 min under constant agitation (1,100 rpm). The papain resin was then washed with digest buffer without DTT using a Pierce centrifuge column. F(ab')$_2$ (0.50 mL, 150.0 μM) was added to the washed papain and the mixture incubated at 37 °C for 20 h under constant agitation (1,100 rpm). The papain resin was then removed from the digest by washing with BBS buffer pH 7.4 (4 × 0.40 mL). The digest was combined with the washes and a 15 mL falcon tube Vivaspin was used to concentrate to a volume of ~1.0 mL. The concentration of the Fab was determined and adjusted to 100 μM with BBS pH 7.4. 50 μL aliquots were stored at -20 °C for up to 6 months.

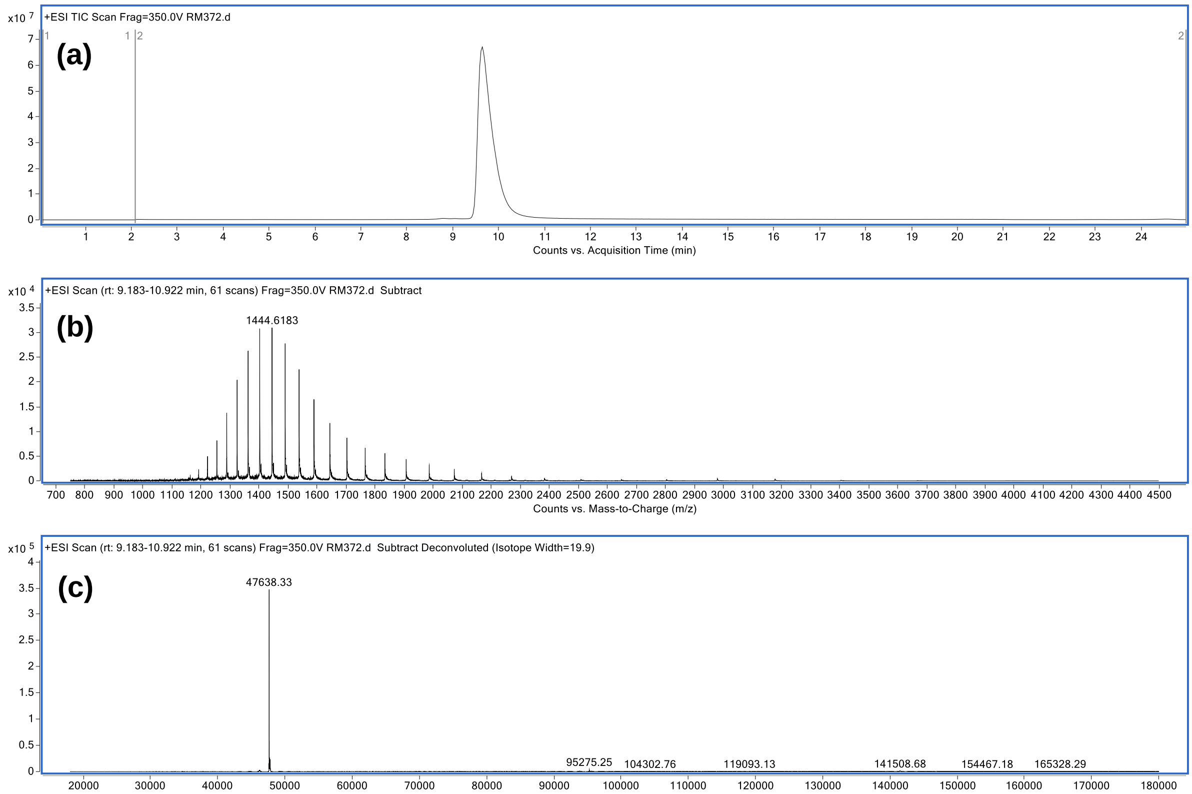

Figure 3: *LCMS of trastuzumab Fab:* ***(a)*** *TIC chromatogram;* ***(b)*** *non-deconvoluted ion-series;* ***(c)*** *deconvoluted mass spectrum, observed 47638 Da.*

2.2. Trastuzumab Fab bioconjugation

Preparation of Fab conjugate 6

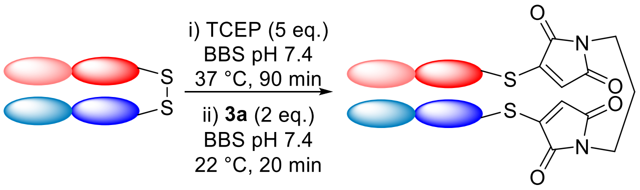

Trastuzumab Fab (100 μM, 50.0 μL) in BBS pH 7.4 was reduced with TCEP (10 mM in dH_2O, 5 eq., 2.50 μL) at 37 °C for 90 min. The excess TCEP was removed *via* ultrafiltration (10 kDa MWCO) into BBS pH 7.4. The concentration was determined and adjusted to 40.0 μM, 106 μL in BBS pH 7.4. To this reduced Fab, bis-bromomaleimide **3a** (4 mM in DMF, 2 eq., 2.12 μL) was added and incubated for 20 min at 22 °C. The excess bis-bromomaleimide **3a** was removed *via* ultrafiltration (10 kDa MWCO) into PBS pH 6.0 to give conjugate **6**, observed by LCMS.

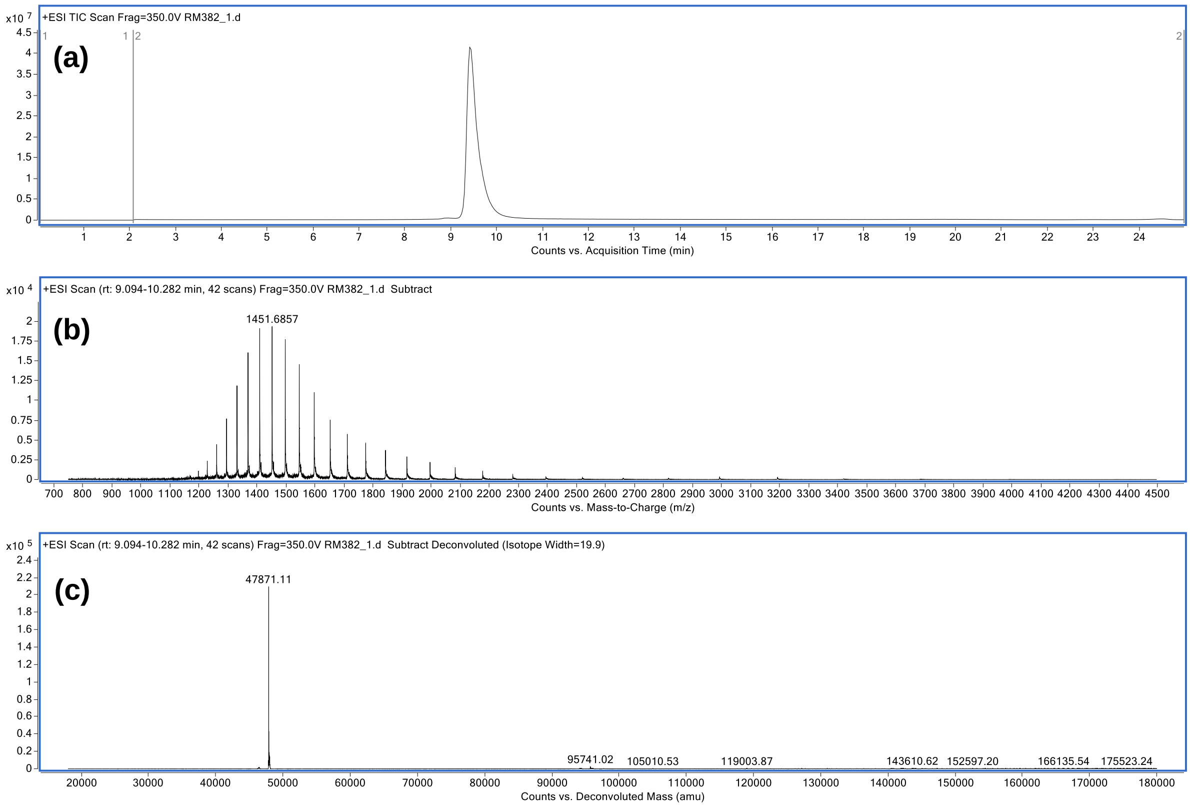

***Figure 4:** LCMS of conjugate **6**: **(a)** TIC chromatogram; **(b)** non-deconvoluted ion-series; **(c)** deconvoluted mass spectrum, expected 47870 Da, observed 47871 Da.*

Irradiation of Fab conjugate 6

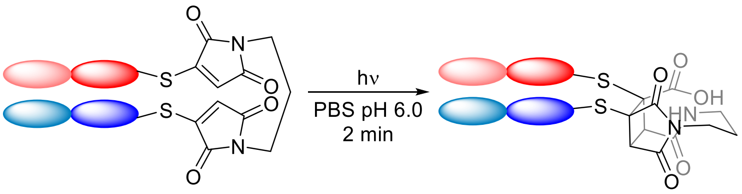

Fab conjugate **6** was adjusted to 40.0 μM, 70.0 μL in PBS pH 6.0. This was then irradiated for 2 min to give hydrolysed conjugate **7**, observed by LCMS.

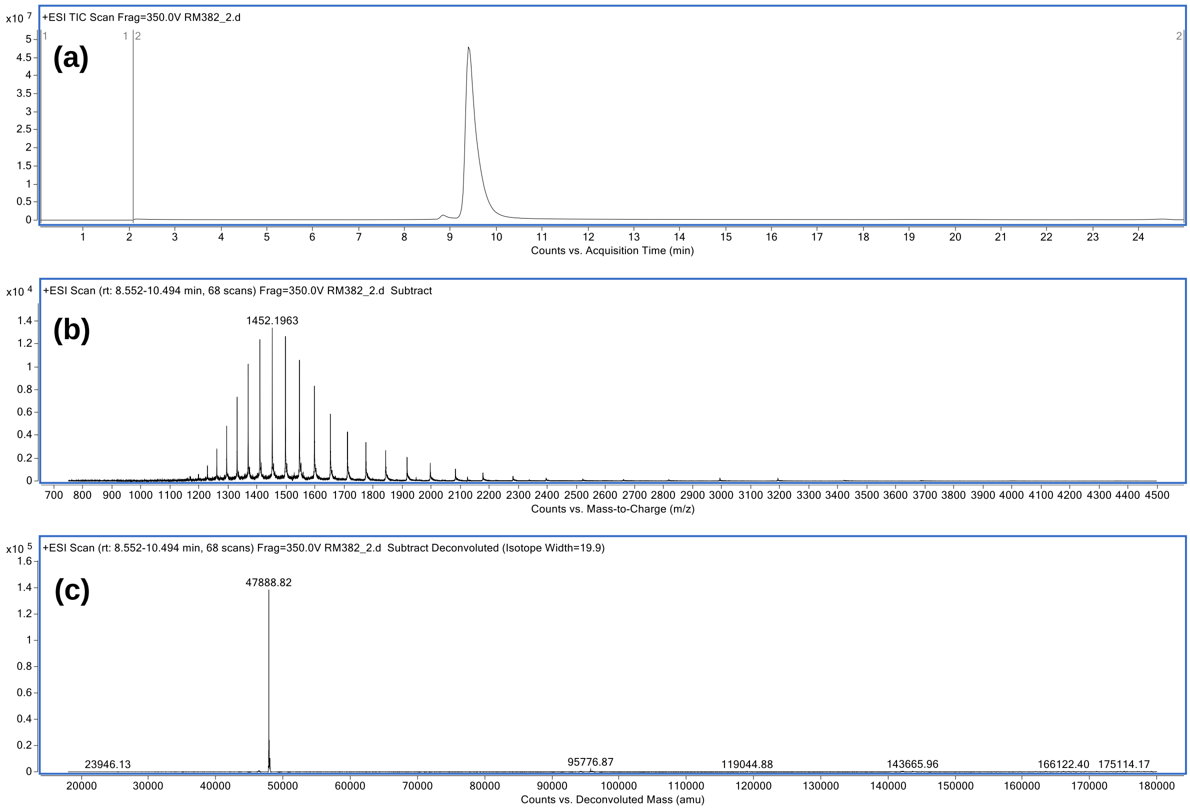

Figure 5: *LCMS of conjugate **7**: **(a)** TIC chromatogram; **(b)** non-deconvoluted ion-series; **(c)** deconvoluted mass spectrum, expected 47888 Da, observed 47889 Da.*

Testing the thiol stability of conjugate 6 with BME

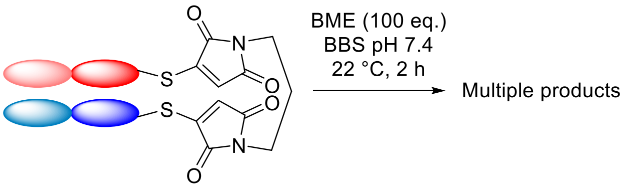

Conjugate **6** was buffer swapped into BBS pH 7.4 *via* ultrafiltration (10 kDa, MWCO). The concentration was determined and adjusted to 34.0 µM, 50.0 µL in BBS pH 7.4. To this, BME (10 mM in dH_2O, 100 eq., 17.0 µL) was added and incubated for 2 h at 22 °C. LCMS observed multiple products.

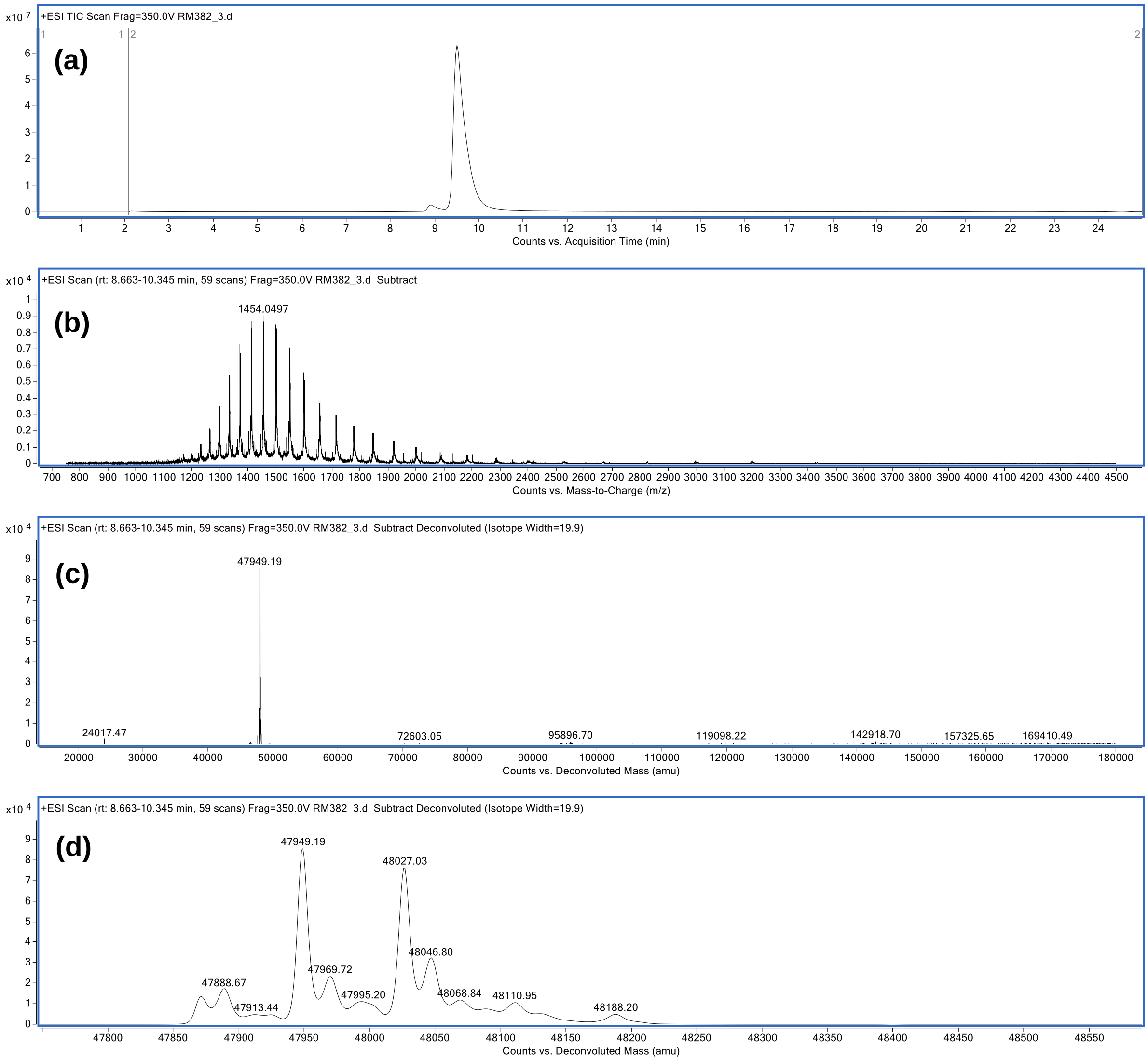

Figure 6: *LCMS upon treatment of conjugate **6** with BME: **(a)** TIC chromatogram; **(b)** non-deconvoluted ion-series; **(c)** deconvoluted mass spectrum; **(d)** zoomed in deconvoluted mass spectrum. Single addition of BME, expected 47948 Da, observed 47949 Da. Double addition of BME, expected 48026 Da, observed 48027 Da.*

Testing the thiol stability of conjugate 6 with EDT

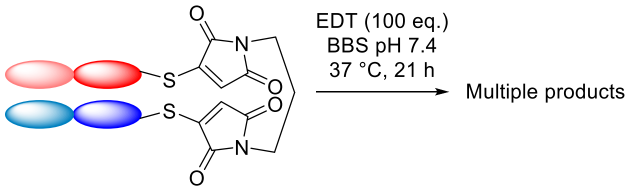

Conjugate **6** was buffer swapped into BBS pH 7.4 *via* ultrafiltration (10 kDa, MWCO). The concentration was determined and adjusted to 50.0 μM, 62.6 μL in BBS pH 7.4. To this, EDT (50 mM in DMF, 100 eq., 6.26 μL) was added and incubated for 21 h at 37 °C. LCMS observed multiple products.

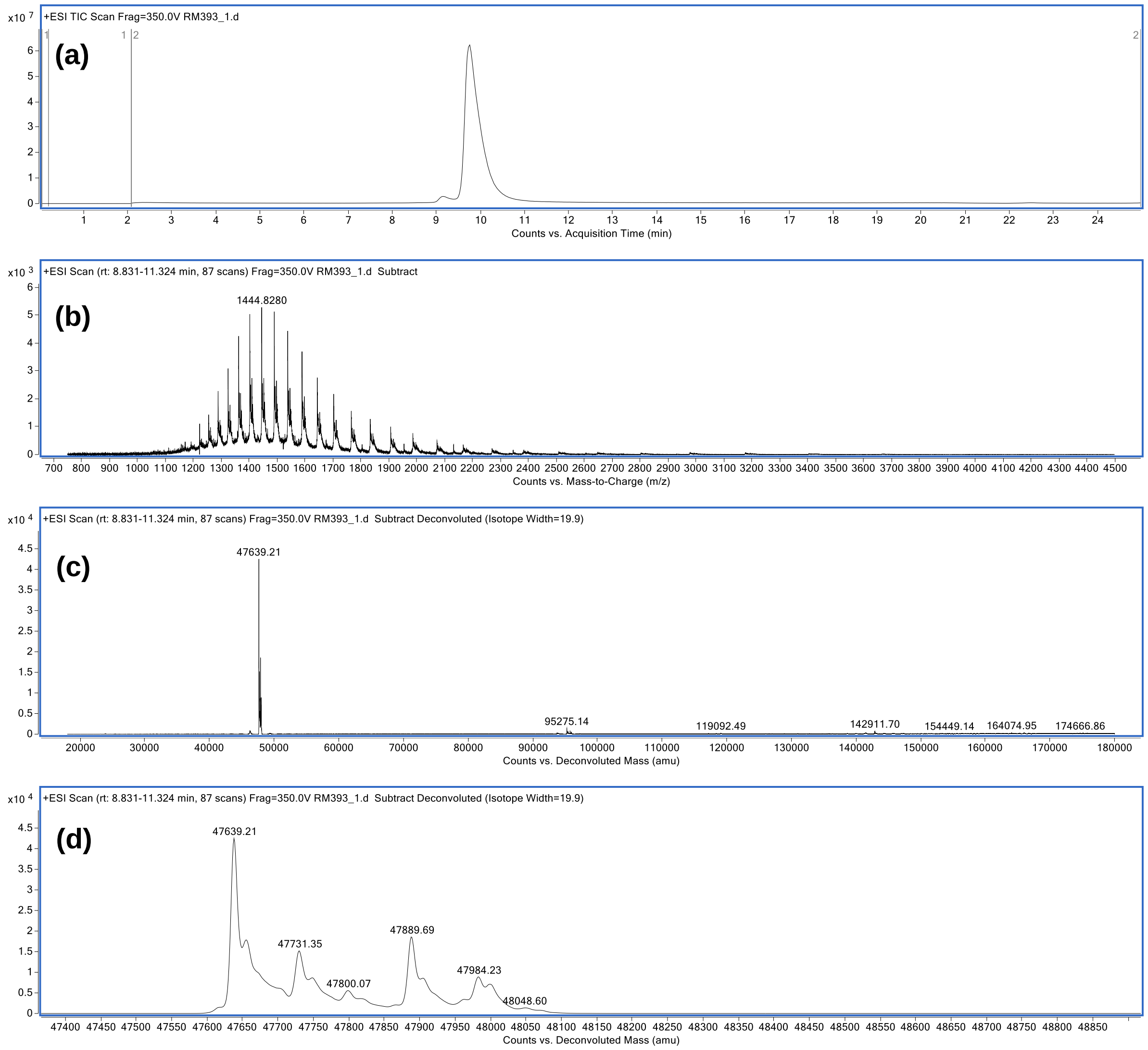

***Figure 7:** LCMS upon treatment of conjugate **6** with EDT: **(a)** TIC chromatogram; **(b)** non-deconvoluted ion-series; **(c)** deconvoluted mass spectrum; **(d)** zoomed in deconvoluted mass spectrum. Cleaved and re-oxidised Fab, expected 47638 Da, observed 47639 Da. EDT scrambled rebridged Fab, expected 47730 Da, observed 47731 Da. Single hydrolysis of conjugate **6**, expected 47889 Da, observed 47890 Da.*

Testing the thiol stability of conjugate 7 with BME

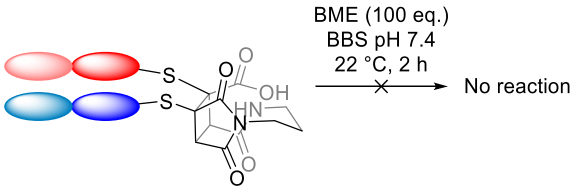

Conjugate **7** was buffer swapped into BBS pH 7.4 *via* ultrafiltration (10 kDa, MWCO). The concentration was determined and adjusted to 34.0 μM, 50.0 μL in BBS pH 7.4. To this, BME (10 mM in dH_2O, 100 eq., 17.1 μL) was added and incubated for 2 h at 22 °C. No reaction was observed by LCMS.

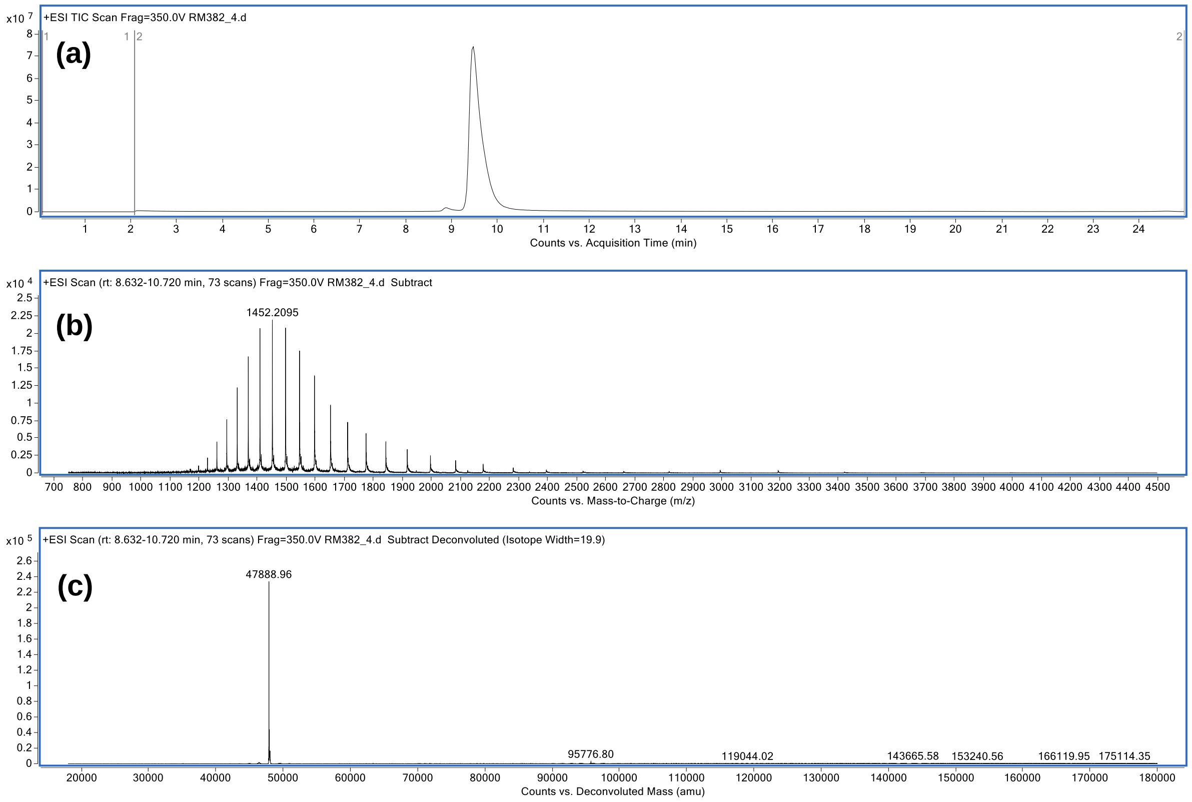

Figure 8: *LCMS upon treatment of conjugate* ***7*** *with BME:* ***(a)*** *TIC chromatogram;* ***(b)*** *non-deconvoluted ion-series;* ***(c)*** *deconvoluted mass spectrum, expected 47888 Da, observed 47889 Da.*

Testing the thiol stability of conjugate 7 with EDT

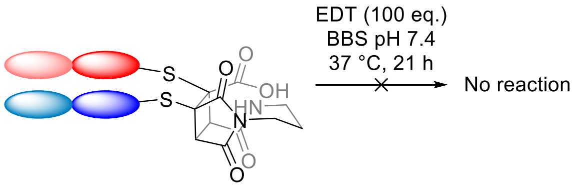

Conjugate **7** was buffer swapped into BBS pH 7.4 *via* ultrafiltration (10 kDa, MWCO). The concentration was determined and adjusted to 50.0 µM, 62.6 µL in BBS pH 7.4. To this, EDT (50 mM in DMF, 100 eq., 6.26 µL) was added and incubated for 21 h at 37 °C. No reaction was observed by LCMS.

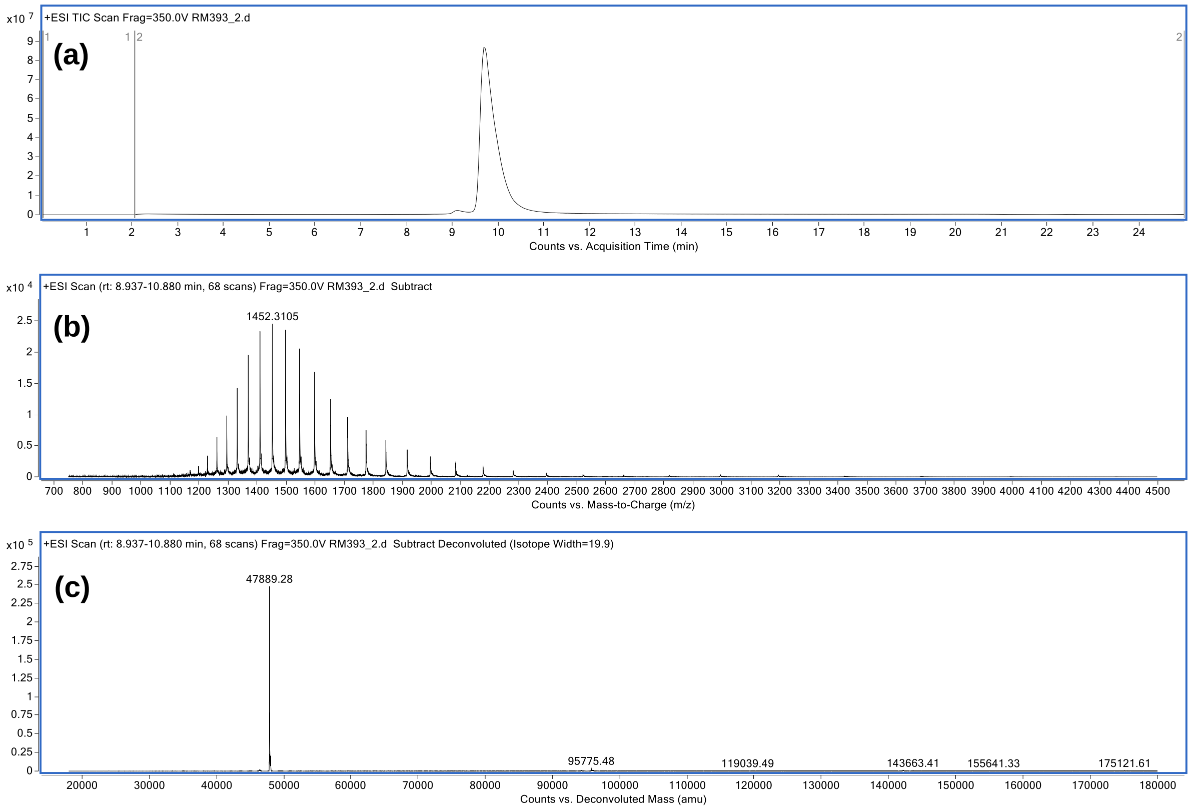

Figure 9: *LCMS upon treatment of conjugate **7** with EDT: **(a)** TIC chromatogram; **(b)** non-deconvoluted ion-series; **(c)** deconvoluted mass spectrum, expected 47888 Da, observed 47889 Da.*

3. *Ab initio* calculations

The model thiomaleimide shown in **Figure 10** was chosen for *ab initio* calculations to reduce computational cost while still capturing the main features of the molecule.

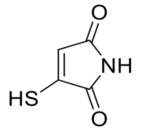

Figure 10: *Model chromophore used for ab initio calculations.*

The structure of the ground state molecule was optimised using DFT with the CAM-B3LYP functional and the 6-311++G basis set in Gaussian 09.[8] Frequency calculations were performed to confirm that a minimum had been achieved. The optimised atomic positions are given in **Table 3**. This optimised structure was then used for calculations of the excited states with the EOM-CCSD method and the 6-311++G** basis set in Q-Chem 5.2.[9] CAS-PT2 calculations were also performed on the model chromophore with the 6-311++G** basis set and an active space with 12 electrons and 8 orbitals in MolPro.[10,11] The active space included six π-orbitals and two n-orbitals with O lone pair character and these MOs are shown in **Figure 11**. Vertical singlet and triplet excitation energies *E* and oscillator strengths *f* for the model thiomaleimide in C_s symmetry calculated using both the EOM-CCSD and CAS-PT2 methods are listed in **Table 4**.

Atom	X / Å	Y / Å	Z / Å
C	-0.05463	-1.04675	0
C	-0.56913	0.38213	0
C	0.48982	1.215472	0
C	1.740237	0.400549	0
H	0.511214	2.299886	0
S	-2.27908	0.718209	0
H	-2.62586	-0.58583	0
O	-0.72272	-2.06316	0
O	2.895254	0.776805	0
N	1.323059	-0.94148	0
H	1.955964	-1.73338	0

Table 3: *Optimised structure for the thiomaleimide model chromophore generated using DFT with the CAM-B3LYP functional and the 6-311++G basis set in Gaussian 09.*[8]

		EOM-CCSD[a]		CAS-PT2[b]	
**State**	**Symmetry**	***E* / eV (*f*)**	***λ* / nm**	***E* / eV (*f*)**	***λ* / nm**
S_1 ($n_1\pi_1$*)	1A''	4.18 (0.00)	297	3.47 (0.00)	358
S_2 ($\pi_1\pi_1$*)	1A'	4.44 (0.09)	280	3.82 (0.09)	325
S_3 ($n_2\pi_1$*)	2A''	4.99 (0.00)	249	3.92 (0.00)	317
S_4 ($\pi_2\pi_1$*)	2A'	5.76 (0.43)	216	5.13 (0.30)	242
T_1 ($\pi_1\pi_1$*)	1A'	3.05	408	2.95	421
T_2 ($n_1\pi_1$*)	1A''	3.89	319	3.50	355

Table 4: *Calculated energies E, oscillator strengths f in parentheses and corresponding wavelengths for electronic transitions from S_0 for the model chromophore in C_s symmetry using [(a)]the EOM-CCSD method with a 6-311++G** basis set and [(b)]the CAS-PT2 method with a 6-311++G** basis set and an active space with 12 electrons and 8 orbitals. Note that the oscillator strengths reported alongside the CAS-PT2 energies are from the CAS-SCF calculation.*

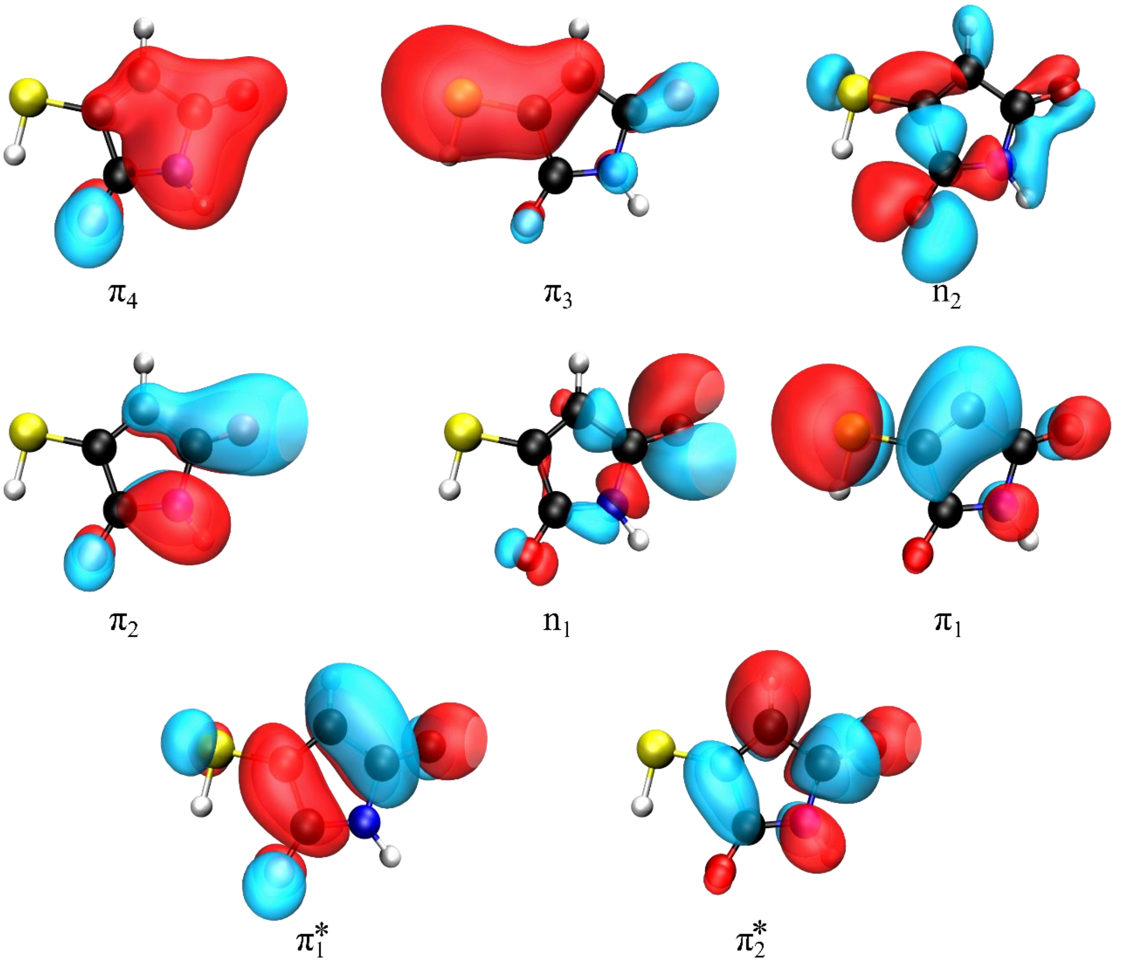

Figure 11: *Molecular orbitals (MOs) included in the active space for the model chromophore. These are generated from ab initio calculations using the CAS-PT2 method, performed using MolPro[10,11] with the 6-311++G** basis set and an active space with 12 electrons and 8 orbitals. Orbitals n_1, π_1 and π_1* are involved in the formation of the S_1 ($n_1\pi_1$*) and S_2 ($\pi_1\pi_1$*) excited states.*

4. Quantum yields

For quantum yield measurements, a fluorescence quartz cuvette placed in a sample holder on a magnetic stirrer was used to provide homogeneous irradiation of the whole sample. The sample was irradiated using a 365 nm UVG2 Labino LED torch held at a distance of 38 cm from the cuvette. The UV absorption was measured simultaneously using an Ocean Optics HR 4000 spectrometer. The spectrometer beam was at a perpendicular pathway to the LED torch beam. The background was measured using a blank sample of MeCN or DCM, as required. The power and photon flux of the LED reaching the cuvette was calculated using *o*-nitrobenzaldehyde as a chemical actinometer, with a known quantum yield Φ of 0.43 ± 0.02.[12] The following equation was used to calculate the LED torch's photon flux, I_0 (M s^{-1}):

$$I_0 = \frac{k_{fit} c_0}{\Phi(1 - 10^{-A_\mathrm{i}})} \quad (1)$$

k_{fit} is the decay constant (s^{-1}) calculated by fitting a mono-exponential growth function to the absorbance vs. time graph at of the reaction of *o*-nitrobenzaldehyde at 283 nm (example in **Figure 12**); c_0 is the initial concentration used (M); Φ = 0.43 ± 0.02 is the quantum yield; and A_i is the initial absorbance at the 365 nm irradiation wavelength.

The power of the LED torch, P was calculated from the following equation:

$$P = \frac{I_0 h c N_\mathrm{A} V}{\lambda} \quad (2)$$

h is Planck's constant (6.63×10^{-34} J s); c is the speed of light (2.998×10^{8} m s^{-1}); N_A is Avogadro's constant (6.02×10^{23} mol^{-1}); V is the reaction volume (0.004 L); and λ is the wavelength of the LED (3.65×10^{-7} m).

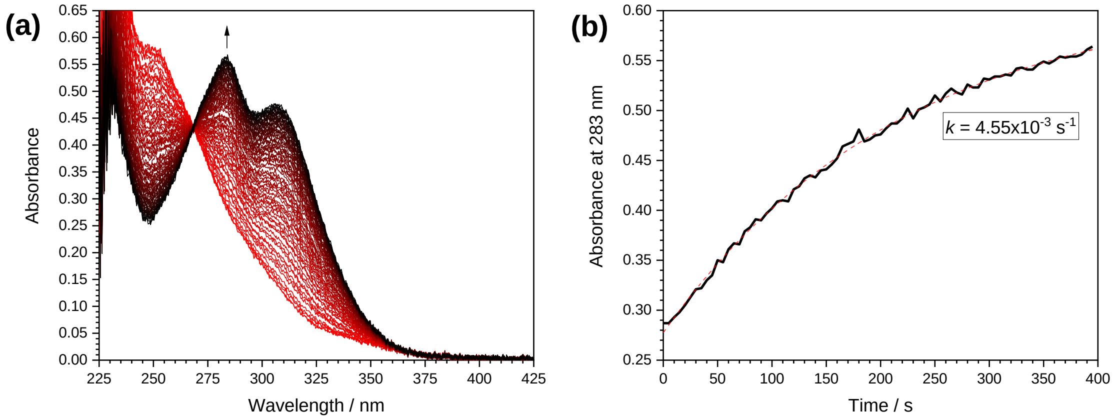

The value of k_{fit} was calculated as 4.55×10^{-3} s^{-1}. It then follows that I_0 = 2.79×10^{-5} M s^{-1} and P = 36.6 mW. These values were consistent for all experiments with an average P = 36.1 mW.

***Figure 12:* (a)** *Change in UV spectrum upon irradiation of o-nitrobenzaldehyde and* **(b)** *absorbance vs. time graph at 283 nm for the conversion of o-nitrobenzaldehyde to o-nitrosobenzoic acid.*

The quantum yield of the maleimide consumption, $\Phi_{reactant}$, was then calculated with the following equation:

$$\Phi_{\mathrm{reactant}} = \frac{r}{I_{\mathrm{abs}}} \quad (3)$$

r is the initial rate of change in concentration (M s^{-1}) measured by the gradient of the concentration vs. time graph up to ~10% conversion (to keep the absorbance at the irradiation wavelength constant) (example in **Figure 13**);[13] and I_{abs} (M s^{-1}) is the amount of photons absorbed by the sample. This is calculated from the equation:

$$I_{\mathrm{abs}} = I_0(1 - 10^{-A_{\mathrm{i}}}) \quad (4)$$

The value of $\Phi_{cycloaddition}$ = $\Phi_{reactant}$ for the intramolecular reactions, but $\Phi_{cycloaddition} = \frac{1}{2}\Phi_{reactant}$ for the intermolecular reactions.

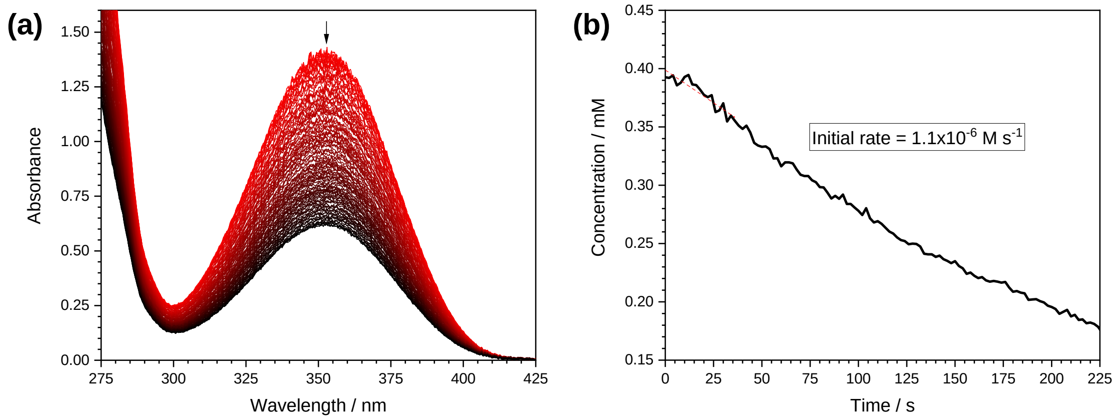

The initial rate was calculated as 1.1×10^{-6} M s^{-1}. It then follows that $\Phi_{reactant}$ = 0.043 and thus $\Phi_{cycloaddition}$ = 0.022.

Figure 13:* (a)** *Change in UV spectrum upon irradiation of ~0.5 mM thiomaleimide* ***1 *and* ***(b)*** *concentration vs. time graph for the conversion of thiomaleimide* ***1*** *to cyclobutane* ***2****.*

The errors associated with the quantum yield, $\Phi_{cycloaddition}$, have been determined by considering the error associated with quantum yield of *o*-nitrobenzaldehyde (0.43 ± 0.02), the error in the stock concentration, the error in the decay constant, k_{fit}, and error in the fitting of the initial rate, r.

5. TEAS and TVAS experiments

Transient absorption experiments were performed at the Warwick Centre for Ultrafast Spectroscopy. The solutions were made up in spectroscopic grade MeCN to concentrations of 20 mM for thiomaleimide **1** and 5 mM for bis-thiomaleimide **4a** and circulated through a Harrick cell using a peristaltic pump from a 50 mL reservoir, allowing fresh sample to always be present. For both TEAS and TVAS, a Ti:sapphire oscillator and amplifier (Spectra-Physics) produced laser pulses of ~40 fs duration centred around 800 nm with a repetition rate of 1 kHz. A Light Conversion TOPAS-Prime optical parametric amplifier with UV extension was used to generate an excitation pulse centred at 354 nm at 500 μW with a pulse duration of <80 fs. For TEAS, a portion of the 800 nm light from the amplifier was focused onto a CaF_2 window to produce a white light continuum probe pulse (~325-680 nm). For TVAS, tuneable infrared light was generated by a second optical parametric amplifier (Light Conversion TOPAS-C), operating in the 1-15 μm range and with a bandwidth of hundreds of nm (~1750-1490 cm^{-1}). For both TEAS and TVAS, the linear polarisation vectors of the pump and probe laser pulses were aligned at 54.7° (the magic angle). The changes in optical density (ΔOD) were recorded using a spectrometer (Avantes, AvaSpec-ULS1650F) for TEAS and using a Horiba iHR320 imaging spectrometer with a mercury cadmium telluride detector array for TVAS. The delay between the pump and probe pulses was controlled using a 500 mm delay stage, giving a maximum delay of 2.5 ns. The TEAS data was chirp-corrected using the KOALA package.[14] Regions of the TEAS and TVAS spectra where residual pump or ground state bleach (GSB) obscured the data were removed prior to analysis and these false colour plots are presented in the main text. The original false colour plots showing signal masking are shown in **Figure 14**.

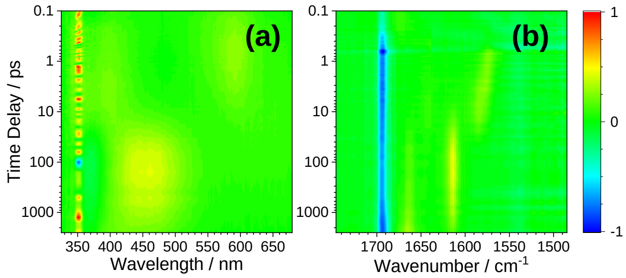

Figure 14: *The original independently normalised false colour plots of the* ***(a)*** *chirp-corrected TEAS experiment for 5 mM bis-thiomaleimide* ***4a*** *(actual OD: -5.7×10^{-3} – 5.7×10^{-3}) showing the major residual pump signal at ~354 nm and* ***(b)*** *TVAS experiment for 5 mM bis-thiomaleimide* ***4a*** *(actual OD: -3.46×10^{-3} – 3.46×10^{-3}) showing a major ground state bleach (GSB) at 1690 cm^{-1}.*

Global analysis using the Glotaran software[15] was employed to determine the different lifetimes involved in the excited state dynamics. An instrument response function (IRF) was

included due to early time features. For the TEAS experiments, wavelengths below 380 nm were not included in the global analysis due to interference from the GSB and residual pump in this region. For the TVAS experiments, the global analysis was only conducted between 1560 and 1630 cm^{-1} as this region contained the key bands. It was not feasible to include the band at 1665 cm^{-1} due to very few data points at longer timescales. The IRF values are summarised in **Table 5** and the Decay Associated Spectra (DAS) are shown in **Figure 15**.

	TEAS		TVAS	
**IRF**	**Thiomaleimide 1**	**Bis-thiomaleimide 4a**	**Thiomaleimide 1**	**Bis-thiomaleimide 4a**
t_0	-0.0325 ± 0.0003	0.0232 ± 0.0005	0.717 ± 0.003	0.620 ± 0.004
σ	0.1743 ± 0.0005	0.1693 ± 0.0006	0.115 ± 0.004	0.043 ± 0.005

Table 5: *Summary of IRF values in ps generated from the global fittings using the Glotaran software.*[15] *The values of σ generated from the Glotaran fitting of the TEAS data are in agreement with those obtained from a later solvent-only scan (0.167 ps).*

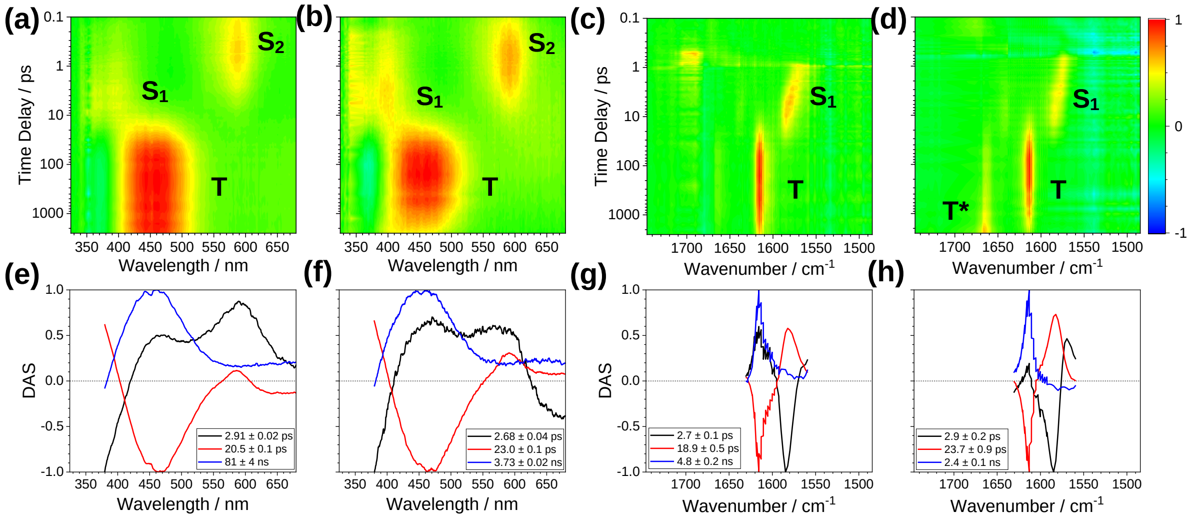

Figure 15: *Independently normalised chirp corrected TEAS spectra for* ***(a)*** *20 mM thiomaleimide* ***1*** *(OD range: −7.2×10^{-3} – 7.2×10^{-3}) and* ***(b)*** *5 mM bis-thiomaleimide* ***4a*** *(OD range: −2.45×10^{-3} – 2.45×10^{-3}) taken following photoexcitation at 354 nm pump in MeCN. Independently normalised TVAS spectra for* ***(c)*** *20 mM thiomaleimide* ***1*** *(OD range: −8.8×10^{-4} – 8.8×10^{-4}) and* ***(d)*** *5 mM bis-thiomaleimide* ***4a*** *(OD range: −1.81×10^{-3} – 1.81×10^{-3}) taken following photoexcitation at 354 nm pump in MeCN. Decay Associated Spectra (DAS) generated from global fitting analysis undertaken using the Glotaran software15 for the* ***(e)*** *TEAS of thiomaleimide* ***1****;* ***(f)*** *TEAS of bis-thiomaleimide* ***4a****;* ***(g)*** *TVAS of thiomaleimide* ***1****; and* ***(h)*** *TVAS of bis-thiomaleimide* ***4a****.*

The spectral profile at different timepoints for the TEAS data is shown in **Figure 16**. Bis-thiomaleimide **4a** shows longer time delay features for the bands at 585 and 385 nm, which is not observed for thiomaleimide **1**.

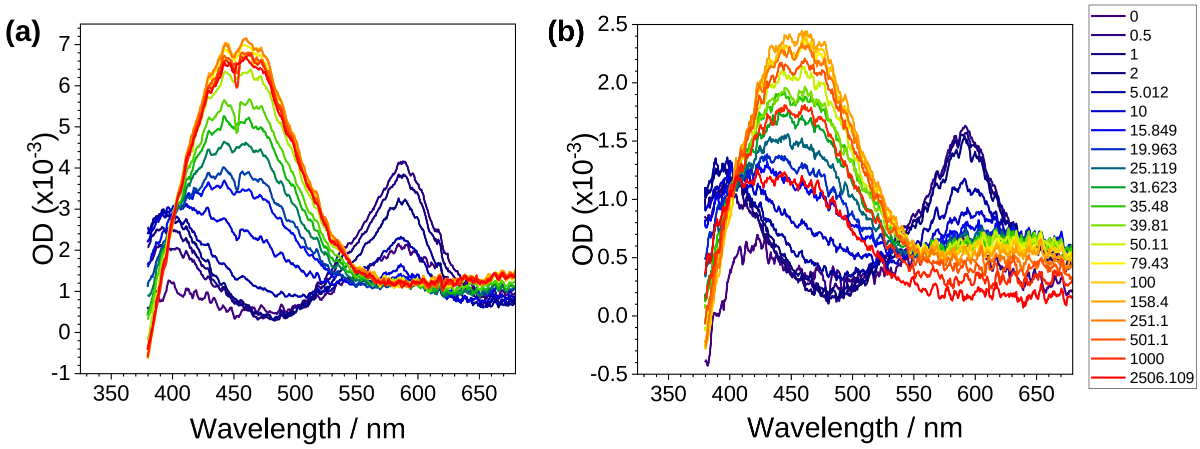

Figure 16: *The spectral profile at different time delays for the TEAS of* ***(a)*** *thiomaleimide* ***1*** *and* ***(b)*** *bis-thiomaleimide* ***4a****.*

6. References

1 P. Boule and J. Lemaire, *J. Chim. Phys. physico-chimie Biol.*, 1980, **77**, 161–165.

2 L. M. Tedaldi, M. E. B. Smith, R. I. Nathani and J. R. Baker, *Chem. Commun.*, 2009, 6583–6585.

3 L. M. Tedaldi, A. E. Aliev and J. R. Baker, *Chem. Commun.*, 2012, **48**, 4725–4727.

4 R. C. Clevenger and K. D. Turnbull, *Synth. Commun.*, 2000, **30**, 1379–1388.

5 J. Put and F. C. De Schryver, *J. Am. Chem. Soc.*, 1973, **95**, 137–145.

6 A. Maruani, M. E. B. Smith, E. Miranda, K. A. Chester, V. Chudasama and S. Caddick, *Nat. Commun.*, 2015, **6**, 6645.

7 L. Castañeda, A. Maruani, F. F. Schumacher, E. Miranda, V. Chudasama, K. A. Chester, J. R. Baker, M. E. B. Smith and S. Caddick, *Chem. Commun.*, 2013, **49**, 8187.

8 M. J. Frisch, G. W. Trucks, H. B. Schlegel, G. E. Scuseria, M. A. Robb, J. R. Cheeseman, G. Scalmani, V. Barone, G. A. Petersson, H. Nakatsuji, X. Li, M. Caricato, A. Marenich, J. Bloino, B. G. Janesko, R. Gomperts, B. Mennucci, H. P. Hratchian, J. V. Ortiz, A. F. Izmaylov, J. L. Sonnenberg, D. Williams-Young, F. Ding, F. Lipparini, F. Egidi, J. Goings, B. Peng, A. Petrone, T. Henderson, D. Ranasinghe, V. G. Zakrzewski, J. Gao, N. Rega, G. Zheng, W. Liang, M. Hada, M. Ehara, K. Toyota, R. Fukuda, J. Hasegawa, M. Ishida, T. Nakajima, Y. Honda, O. Kitao, H. Nakai, T. Vreven, K. Throssell, J. A. Montgomery, Jr., J. E. Peralta, F. Ogliaro, M. Bearpark, J. J. Heyd, E. Brothers, K. N. Kudin, V. N. Staroverov, T. Keith, R. Kobayashi, J. Normand, K. Raghavachari, A. Rendell, J. C. Burant, S. S. Iyengar, J. Tomasi, M. Cossi, J. M. Millam, M. Klene, C. Adamo, R. Cammi, J. W. Ochterski, R. L. Martin, K. Morokuma, O. Farkas, J. B. Foresman and D. J. Fox, *Gaussian 09, Revis. C.01*.

9 Y. Shao, Z. Gan, E. Epifanovsky, A. T. B. Gilbert, M. Wormit, J. Kussmann, A. W. Lange, A. Behn, J. Deng, X. Feng, D. Ghosh, M. Goldey, P. R. Horn, L. D. Jacobson, I. Kaliman, R. Z. Khaliullin, T. Kuś, A. Landau, J. Liu, E. I. Proynov, Y. M. Rhee, R. M. Richard, M. A. Rohrdanz, R. P. Steele, E. J. Sundstrom, H. L. Woodcock, P. M. Zimmerman, D. Zuev, B. Albrecht, E. Alguire, B. Austin, G. J. O. Beran, Y. A. Bernard, E. Berquist, K. Brandhorst, K. B. Bravaya, S. T. Brown, D. Casanova, C.-M. Chang, Y. Chen, S. H. Chien, K. D. Closser, D. L. Crittenden, M. Diedenhofen, R. A. DiStasio, H. Do, A. D. Dutoi, R. G. Edgar, S. Fatehi, L. Fusti-Molnar, A. Ghysels, A. Golubeva-Zadorozhnaya, J. Gomes, M. W. D. Hanson-Heine, P. H. P. Harbach, A. W. Hauser, E. G. Hohenstein, Z. C. Holden, T.-C. Jagau, H. Ji, B. Kaduk, K. Khistyaev, J. Kim, J. Kim, R. A. King, P. Klunzinger, D. Kosenkov, T. Kowalczyk, C. M. Krauter, K. U. Lao, A. D. Laurent, K. V. Lawler, S. V. Levchenko, C. Y. Lin, F. Liu, E. Livshits, R. C. Lochan, A.

Luenser, P. Manohar, S. F. Manzer, S.-P. Mao, N. Mardirossian, A. V. Marenich, S. A. Maurer, N. J. Mayhall, E. Neuscamman, C. M. Oana, R. Olivares-Amaya, D. P. O'Neill, J. A. Parkhill, T. M. Perrine, R. Peverati, A. Prociuk, D. R. Rehn, E. Rosta, N. J. Russ, S. M. Sharada, S. Sharma, D. W. Small, A. Sodt, T. Stein, D. Stück, Y.-C. Su, A. J. W. Thom, T. Tsuchimochi, V. Vanovschi, L. Vogt, O. Vydrov, T. Wang, M. A. Watson, J. Wenzel, A. White, C. F. Williams, J. Yang, S. Yeganeh, S. R. Yost, Z.-Q. You, I. Y. Zhang, X. Zhang, Y. Zhao, B. R. Brooks, G. K. L. Chan, D. M. Chipman, C. J. Cramer, W. A. Goddard, M. S. Gordon, W. J. Hehre, A. Klamt, H. F. Schaefer, M. W. Schmidt, C. D. Sherrill, D. G. Truhlar, A. Warshel, X. Xu, A. Aspuru-Guzik, R. Baer, A. T. Bell, N. A. Besley, J.-D. Chai, A. Dreuw, B. D. Dunietz, T. R. Furlani, S. R. Gwaltney, C.-P. Hsu, Y. Jung, J. Kong, D. S. Lambrecht, W. Liang, C. Ochsenfeld, V. A. Rassolov, L. V. Slipchenko, J. E. Subotnik, T. Van Voorhis, J. M. Herbert, A. I. Krylov, P. M. W. Gill and M. Head-Gordon, *Mol. Phys.*, 2015, **113**, 184–215.

10 H.-J. Werner, P. J. Knowles, G. Knizia, F. R. Manby and M. Schütz, *Wiley Interdiscip. Rev. Comput. Mol. Sci.*, 2012, **2**, 242–253.

11 H.-J. Werner, P. J. Knowles, F. R. Manby, J. A. Black, K. Doll, A. Heßelmann, D. Kats, A. Köhn, T. Korona, D. A. Kreplin, Q. Ma, T. F. Miller, A. Mitrushchenkov, K. A. Peterson, I. Polyak, G. Rauhut and M. Sibaev, *J. Chem. Phys.*, 2020, **152**, 144107.

12 E. Stadler, A. Eibel, D. Fast, H. Freißmuth, C. Holly, M. Wiech, N. Moszner and G. Gescheidt, *Photochem. Photobiol. Sci.*, 2018, **17**, 660–669.

13 D. M. E. Davies, C. Murray, M. Berry, A. J. Orr-Ewing and K. I. Booker-Milburn, *J. Org. Chem.*, 2007, **72**, 1449–1457.

14 M. P. Grubb, A. J. Orr-Ewing and M. N. R. Ashfold, *Rev. Sci. Instrum.*, 2014, **85**, 064104.

15 J. J. Snellenburg, S. P. Laptenok, R. Seger, K. M. Mullen and I. H. M. van Stokkum, *J. Stat. Softw.*, 2012, **49**, 1–22.
